# Chitosan and Chitin Deacetylase Activity Are Necessary for Development and Virulence of Ustilago maydis

**DOI:** 10.1128/mBio.03419-20

**Published:** 2021-03-02

**Authors:** Yanina S. Rizzi, Petra Happel, Sandra Lenz, Mounashree J. Urs, Martin Bonin, Stefan Cord-Landwehr, Ratna Singh, Bruno M. Moerschbacher, Regine Kahmann

**Affiliations:** a Max Planck Institute for Terrestrial Microbiology, Department of Organismic Interactions, Marburg, Germany; b Westfälische Wilhelms-Universität Münster, Institut für Biologie und Biotechnologie der Pflanzen, Münster, Germany; Universidad de Córdoba

**Keywords:** chitin deacetylase, *Ustilago maydis*, viability, virulence, *Zea mays*, chitin, chitosan

## Abstract

The biotrophic fungus Ustilago maydis harbors a chitin deacetylase (CDA) family of six active genes as well as one pseudogene which are differentially expressed during colonization. This includes one secreted soluble CDA (Cda4) and five putatively glycosylphosphatidylinositol (GPI)-anchored CDAs, of which Cda7 belongs to a new class of fungal CDAs. Here, we provide a comprehensive functional study of the entire family. While budding cells of *U. maydis* showed a discrete pattern of chitosan staining, biotrophic hyphae appeared surrounded by a chitosan layer. We purified all six active CDAs and show their activity on different chitin substrates. Single as well as multiple *cda* mutants were generated and revealed a virulence defect for mutants lacking *cda7*. We implicated *cda4* in production of the chitosan layer surrounding biotrophic hyphae and demonstrated that the loss of this layer does not reduce virulence. By combining different *cda* mutations, we detected redundancy as well as specific functions for certain CDAs. Specifically, certain combinations of mutations significantly affected virulence concomitantly with reduced adherence, appressorium formation, penetration, and activation of plant defenses. Attempts to inactivate all seven *cda* genes simultaneously were unsuccessful, and induced depletion of *cda2* in a background lacking the other six *cda* genes illustrated an essential role of chitosan for cell wall integrity.

## Abstract (precis)

The basidiomycete Ustilago maydis causes smut disease in maize, causing substantial losses in world corn production. This nonobligate pathogen penetrates the plant cell wall with the help of appressoria and then establishes an extensive biotrophic interaction, where the hyphae are tightly encased by the plant plasma membrane.

## INTRODUCTION

When fungi infect plant hosts, conserved microbe-associated molecular patterns (MAMPs) induce pattern-triggered immunity (PTI). One of the most efficient fungal MAMPs is chitin, a polymer of β-1,4-linked *N*-acetylglucosamine (GlcNAc) that forms rigid microfibrils and is an essential structural component of fungal cell walls. To trigger PTI, chitin oligomers bind to LysM receptor-like kinases or receptor-like proteins residing in the plant plasma membrane. Critical for receptor binding are the acetyl groups in GlcNAc (1–4). Fungal plant pathogens have developed several strategies to avoid chitin recognition. These include shielding the chitin layer from attack by host chitinases by cell wall modifications such as the production of α-1,3-glucan (5, 6) or by fungal effectors which bind cell wall chitin (7–9). Another strategy relies on the production of LysM domain effectors that bind apoplastic chitin oligosaccharides (10–13). A less explored strategy involves the transformation of chitin to chitosan by chitin deacetylases (CDAs). Chitosan is a poor substrate for chitinases and does not activate chitin receptors if fully deacetylated (2, 4, 14–17). CDAs (EC 3.5.1.41) are the enzymes that remove the acetyl group from chitin and convert it to chitosans, polymers of β-1,4-glucosamine and β-1,4-*N*-acetylglucosamine (18, 19). CDAs belong to the carbohydrate esterase family 4 (CE4), whose members contain a prototypical NodB domain that houses the catalytic core. The majority of fungal CDAs are attached to the fungal membrane/cell wall via glycosylphosphatidylinositol (GPI) anchors, while other members are secreted (20).

Chitosan has been shown to decorate invasive hyphae of rust fungi, where it is considered to protect hyphae from attack by plant chitinases (21). CDA genes in plant-pathogenic fungi and fungi associated with plants usually exist in gene families (22–24), which has slowed down comprehensive analyses. Members with a GPI anchor are likely to act in concert with chitin synthases on nascent chitin chains. CDAs without a GPI anchor such as PDA1 from the cotton pathogen Verticillium dahliae (*Vd*PDA1) are involved in deacetylating and inactivating elicitor-active chitin oligomers (14) or deacetylating surface-exposed chitin on fungal hyphae, like Pst_13661 from the wheat pathogen Puccinia striiformis f. sp. *tritici* (25). Through this, chitin-triggered immunity is prevented and virulence is promoted. In the rice pathogen Magnaporthe oryzae, chitosan was detected in germ tubes and appressoria, and it was shown that three genes are responsible for this distribution. A triple mutant lacking these genes was severely attenuated in adhesion and appressorium development on artificial surfaces but was unaffected in virulence (22, 23). As there are nine putative *cda* genes in *M. oryzae*, an involvement of the other members in virulence is still an option. In the human pathogen Cryptococcus neoformans, chitosan is uniformly distributed in the cell wall. Mutants lacking all three *cda* genes showed increased chitin staining, had cell separation and cell wall integrity defects, and were completely avirulent in a mouse model (26). C. neoformans harbors a fourth *cda* gene recently described as the first chitosan deacetylase (27). Collectively, these studies reveal a plethora of functions conferred by chitosan and the responsible CDAs but leave many questions unanswered, in particular, with respect to redundancy, specificity, primary and secondary effects, localization of chitosan, and host immune responses.

In this communication, we study CDAs in the plant-pathogenic basidiomycete Ustilago maydis. This fungus causes corn smut, a disease associated with prominent tumor development on all aboveground parts of the plant, leading to substantial crop losses (28). *U. maydis* infects corn as filamentous dikaryon, which is generated after the mating of two compatible yeast-like strains. On the leaf surface, dikaryotic filaments develop unmelanized appressoria from which infectious hyphae emerge. *U. maydis* establishes a biotrophic interaction, in which invasive hyphae become encased by the host plasma membrane. At later stages after nuclear fusion, there is massive hyphal proliferation culminating in the formation of diploid spores (29). Until now, surface-associated proteins which could sequester chitin have not been identified in *U. maydis*. To analyze how this pathogen avoids chitin-triggered immunity, we have investigated the function of the seven putative CDAs in *U. maydis* during development and host colonization.

## RESULTS

### Chitin and chitosan in the cell wall of *U. maydis* during development.

**FIG 1:**
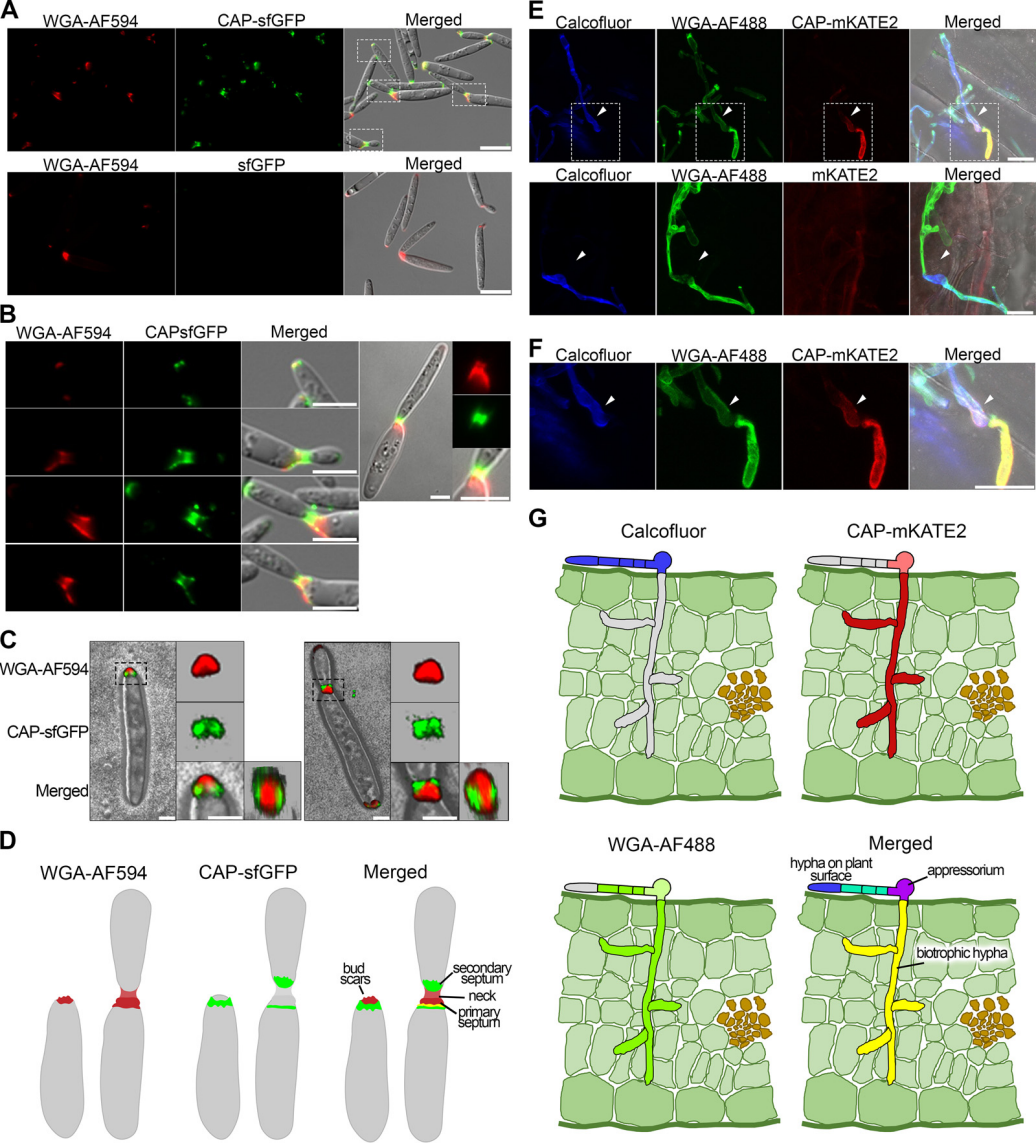
Chitosan and chitin accessibility during different phases of development of *U. maydis* strain SG200. (A) Budding cells were stained with CAP-sfGFP and WGA-AF594 (top) or with sfGFP and WGA-AF594 as control (bottom). Cells were observed by fluorescence microscopy (left, chitin in red; middle, chitosan in green; right, merge of bright field, chitin, and chitosan staining). Scale bars, 10 μm. Representative pictures are from at least three independent experiments. (B) Enlargements of the stippled boxes marked in panel A, except for the rightmost picture showing the division zone of a budding cell where the daughter cell is almost mature. Scale bars, 5 μm. (C) Three-dimensional (3D) reconstruction of the bud scar region (left) as well as the division zone in budding cell (right) stained with CAP-sfGFP and WGA-AF594 by confocal microscopy. The stippled zones in the left panels are enlarged on the right and shown as top view in addition (far right). Scale bars, 5 μm. (D) Scheme showing the distribution of chitin (WGA-AF594, red; left), chitosan (CAP-sfGFP, green; middle), and merged in budding cells with and without a bud. (E) Staining of hyphae during infection at 2 dpi: calcofluor staining (blue) of hyphae on the surface of the plant leaves, WGA-AF488 staining of chitin (green), CAP-mKATE2 staining of chitosan (red), and merged with bright field are shown at the top. Staining in the bottom images was as for the top except that CAP-mKATE2 was replaced with mKATE2 as a nonbinding control. White arrowheads indicate appressoria. The samples were observed by confocal microscopy and all images are projections of multiple z-stacks. Scale bars, 10 μm. Representative pictures from at least three independent experiments are shown. (F) Enlargements of the stippled boxes marked in panel E. White arrowheads indicate appressoria. Scale bar, 10 μm. (G) Scheme showing the staining of hyphae on the leaf surface with calcofluor (blue, top left), distribution of chitin (WGA-AF488, green; bottom left), chitosan (CAP-mKATE2, red; top right), and merged (bottom right).

All following studies were done in *U. maydis* strain SG200, a solopathogenic haploid strain, which can complete the life cycle without a mating partner (30). To detect chitin, fungal cells were stained with wheat germ agglutinin conjugated either to Alexa Fluor 594 (WGA-AF594) or to Alexa Fluor 488 (WGA-AF488). In SG200 cells grown in culture, chitin was detected in the majority of cells at one pole, in cell division zones, and at growing tips of daughter cells (Fig. 1A to C, and shown schematically in Fig. 1D; see also Fig. S1A in the supplemental material). As staining was not always detectable in these locations (Fig. S1A), it is likely that the accessibility of chitin is developmentally regulated. The staining patterns with WGA-AF488 and WGA-AF594 in budding cells were comparable (Fig. S1B and C).

10.1128/mBio.03419-20.1 FIG S1 Staining of chitin and chitosan in budding cells and biotrophic hyphae with WGA and CAP. (A) Quantification of presence of chitin and chitosan staining in budding cells of SG200. Mean values ± SD from 6 independent biological replicates. (B) Budding cells of SG200 were stained with WGA-AF488 (green) to detect chitin and CAP-mKATE2 (red) to detect chitosan (top) or with mKATE2 as a control (bottom). Cells were observed by confocal microscopy. The images are projections of multiple z-stacks. Scale bars, 10 μm. (C) Enlargements of the stippled boxes marked in A. Scale bars, 10 μm. (D and E) Chitosan and chitin accessibility in the cell wall of *U. maydis* during plant infection. Leaf samples infected with the solopathogenic strain SG200 (D) or with FB1 × FB2 (E) were collected at 2, 4, 6, 8, 10, and 12 dpi and stained with calcofluor (blue) for hyphae on the surface of the plant leaves. After digestion of the plant tissue, biotrophic hyphae were stained with WGA-AF488 for chitin (green) and with CAP-mKATE2 for chitosan (red). In addition, bright-field (BF) and merging of the four channels are shown. The samples were observed by confocal microscopy, and all images are projections of multiple z-stacks. Scale bars, 10 μm. Download FIG S1, PDF file, 1.8 MB. Copyright © 2021 Rizzi et al. 2021 Rizzi et al. https://creativecommons.org/licenses/by/4.0/ This content is distributed under the terms of the Creative Commons Attribution 4.0 International license.

To stain chitin in biotrophic hyphae and to discriminate between filaments on the leaf surface and inside leaf tissue, we first stained filaments on the leaf surface at 2 days postinfection (dpi) with calcofluor, which is unable to penetrate the leaf cuticle (Fig. 1E). To visualize chitin in biotrophic hyphae, calcofluor-stained infected leaf tissue was treated with macerozyme and cellulase to loosen the tissue and increase the accessibility of hyphae to WGA-AF488. Filamentous cells on the leaf surface as well as appressoria and filamentous cells *in planta* were uniformly stained by WGA-AF488 (Fig. 1E and F, and shown schematically in Fig. 1G). This chitin staining pattern was also observed during later stages of biotrophic development (Fig. S1D). Since SG200 is affected in proliferation during late stages of biotrophic development (F. Fukada, personal communication), chitin distribution was also visualized after infection with a mixture of compatible haploid cells (Fig. S1E).

To specifically visualize chitosan, we used a chitosan affinity protein (CAP) fused to superfolder green fluorescent protein (sfGFP) for staining, which relies on chitosan binding of an inactive chitosanase (31). This stain was used successfully for detecting chitosan in germinated hyphae and endophytic infection structures of the wheat stem rust fungus (31). Chitosan was detected in the majority of cells at one tip, at the growing pole of emerging buds, and in the cell division zone (Fig. 1 and Fig. S1A). Superimposition of the chitin and chitosan stains revealed that chitosan occurred in patches flanking the chitin layer that covers the bud scars (Fig. 1C, and schematically shown in Fig. 1D). In the division zone, chitin and chitosan occurred mostly in nonoverlapping patches, with chitin accumulating at the neck and at the region closest to the mother cell, most likely the primary septum, while the secondary septum in daughter cells usually stained for chitosan (Fig. 1A to C, and shown schematically in Fig. 1D).

To visualize chitosan in hyphae after infection, we used again an initial staining with calcofluor, followed by tissue permeabilization as described above for chitin staining, and then stained with CAP-mKATE2. Costaining with WGA-AF488 revealed that hyphae and appressoria on the leaf surface stained only weakly for chitosan, but biotrophic hyphae at 2 dpi stained very strongly for chitosan all around the hyphae (Fig. 1E and F, and schematically shown in Fig. 1G). As control for the specificity of CAP-mKATE2 staining, CAP-mKATE2 was replaced by the fluorescent mKATE2 protein lacking CAP; in this case, hyphae were not stained (Fig. 1E, bottom). The pattern of chitosan staining remained essentially unchanged throughout the life cycle (Fig. S1D and E), and chitosan was also detected in spores after FB1 × FB2 infections (Fig. S1E).

### *U. maydis* harbors seven putative chitin deacetylases.

**FIG 2:**
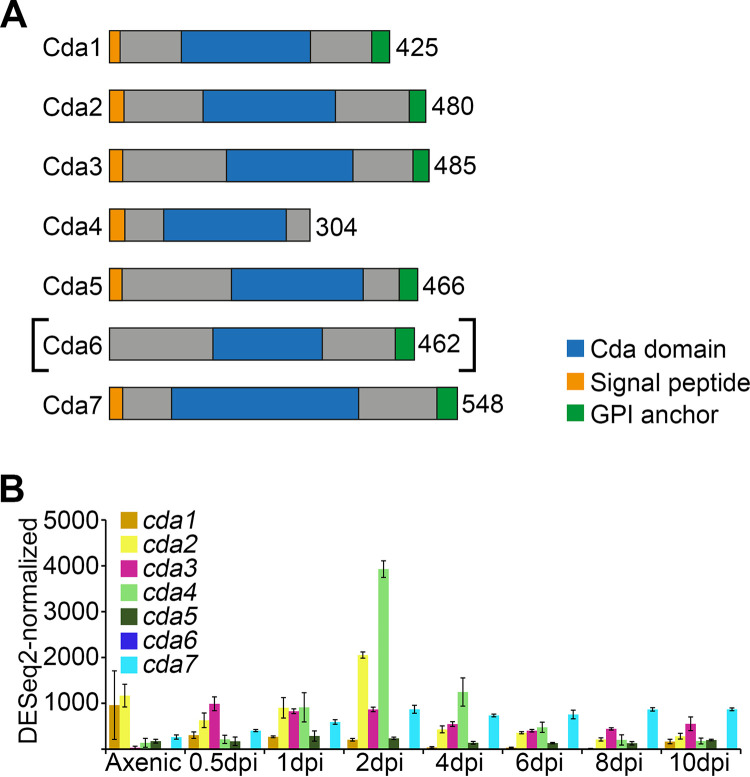
The CDA gene family in *U. maydis*. (A) Schematic of the seven putative CDA proteins indicating a predicted signal peptide (orange), a predicted GPI anchor (green), and the NodB homology domain (blue). The numbers on the right refer to amino acids. Brackets indicate that *cda6* is likely a pseudogene, and the protein annotated in NCBI under accession number 23565582 might be incorrect. (B) Expression pattern of *cda* genes during growth in axenic culture and at different time points (0.5, 1, 2, 4, 6, 8, and 12 days) during plant infection with FB1 × FB2. Data were retrieved from an RNA-seq analysis (29). Error bars indicate ± standard deviation (SD).

An InterPro search for proteins with a NodB homology domain (IPR002509) initially identified eight proteins in *U. maydis* (see Fig. S2A and B). Of these, seven proteins display the five motifs characteristic for the catalytic site of CDAs (32, 33) (Fig. S2A), and these were designated *cda1* to *cda7*. The remaining protein designated PuuE1 carries substitutions in the metal binding triad characteristic for PuuE allantoinases (34) (Fig. S2B) and was not analyzed here. The amino acid identity between the seven putative CDAs is low and ranges from 15.9% to 46.6% (Fig. S2C). Cda7, with the lowest identity to the others, displays an insert of 61 amino acids between motifs 3 and 4 (Fig. S2A). Such a loop region was also detected in Cda1 and Cda8 of the silk worm Bombyx mori (Fig. S2A). Except for Cda4, all putative *U. maydis* CDA proteins have a predicted GPI anchor at the C terminus (predicted with PredGPI) (Fig. 2A). SignalP predicted N-terminal signal peptides in Cda1, -2, -3, -4, -5, and -7 but not in Cda6. A comparison with Cda6 orthologs from Ustilaginaceae revealed that the corresponding genes were not predicted to contain introns, and the predicted proteins contained signal peptides (Fig. S2D). When the open reading frame (ORF) encoding the central part of Cda6 was extended manually toward the 5′ and 3′ end without assuming introns, several stop codons were predicted, making it likely that *cda6* is a pseudogene (Fig. S2E).

10.1128/mBio.03419-20.2 FIG S2 Analysis of sequences of proteins containing a NodB domain from *U. maydis*. (A) Alignment of the NodB domains (defined by InterPro) of CDAs from *U. maydis* (*Um*Cda1, *Um*Cda2, *Um*Cda3, *Um*Cda4, *Um*Cda5, *Um*Cda6, and *Um*Cda7) and of the CDA protein from *C. lindemuthianum* (*C.l*Cda), Cda1 from S. cerevisiae (*Sc*Cda1), and *B. mori* Cda1 and Cda8 (*B.m*Cda1 and *B.m*Cda8) using Clustal Omega 12.1 (F. Madeira, Y. M. Park, J. Lee, N. Buso, T. Gur, N. Madhusoodanan, P. Basutkar, A. R. N. Tivey, S. C. Potter, R. D. Finn, R. Lopez. Nucleic Acids Res 47:W636–W641, 2019, https://doi.org/10.1093/nar/gkz268.). Accession numbers of the respective genes are listed in Table S1E. The conserved motifs for CDA catalytic activity are highlighted in green, and conserved active site residues within these motifs are marked with red arrowheads. Conserved residues for zinc binding are marked with blue arrowheads. *Um*Cda7, *B.m*Cda1, and *B.m*Cda8 contain an insertion between motif 3 and motif 4, and this was removed to improve the alignment. The insert in *Um*Cda7 is STASVPVTDPNTDAFWPYTLDNGMANDCNSVANICGGQPKLPGFWEIPMYAIFDERGAAGA. In *B.m*Cda1, the insert is ITAPLSNPRLCPYTMYFRMPHRCHGNLQSCPTRSHAVWEMVMNELDRREDPSNDEYLPGC, and in *B.m*Cda8, the insert is DCTWPTTALTNPGLWPYTLHHESIQDCIIPPCPTASIPGPWVLPMISWRDLNNFPC. (B) Alignment of the amino acid sequences of the full length of putative PuuE protein of *U. maydis* (*Um*PuuE), CDA protein from *C. lindemuthianum*, and PuuE protein from *Pseudomonas fluorescence* (*P.f*PuuE) using Clustal Omega 12.1. The conserved residues for CDA activity in *C.l*Cda, marked as in panel A. Characteristic modification of the zinc-binding triad of CDA DHH to EHW in PuuE proteins are marked with yellow arrowheads. (C) Percentage of identity between Cdas from *U. maydis*. Determined with Clustal Omega12.1. (D) Alignment of the nucleotide sequence of *cda6* from *U. maydis* (*umcda6*) and the ORFs of the orthologous genes (identified by Ortho DB v10 and by manual checking of synteny) from Sporisorium scitamineum (*SSCI30930.1*, *sscda6*), Pseudozyma hubeiensis (*PHSY_002331*, *phcda6*), and Pseudozyma brasiliensis (*PSEUBRA_SCAF18g04655*, *pbcda6*) performed with Clustal Omega 12.1. The predicted ATGs and stop codons are highlighted in red, the sequences of the predicted signal peptides are marked in green, and the nucleotides that encode the putative omega site of the GPI anchor are marked in orange. The regions annotated as introns in *umcda6* are highlighted in blue. (E) Nucleotide sequence and translated amino acid sequence of the central part of the ORF of *cda6* from *U. maydis* by ExPASy Translate (http://web.expasy.org/translate/) containing four of the five conserved domains and extended manually toward the 5′ and 3′ end sof the respective gene without assuming introns. As an in-frame ATG would be located 3′ to the first motif, this would reduce the size of Cda6 to 178 amino acids, eliminate the first conserved motif critical for CDA activity, and eliminate the putative GPI anchor. Conserved motifs for CDA activity are highlighted in green, regions annotated as introns are highlighted in blue, an in-frame ATG is marked in magenta, and stop codons are given in red. (F) Phylogenetic tree of fungal CDAs. Amino acid sequences of putative or known fungal chitin deacetylases were retrieved from the NCBI, Ensembl, UniProt, and JGI genome database portals and aligned with the online program MAFFT version 7 (Katoh K, Rozewicki J, Yamada KD. Brief Bioinform 20:1160–1166, 2019). The evolutionary analysis was carried out in MEGA7.0 (S. Kumar, G. Stecher, K. Tamura. Mol Biol Evol 33:1870–1874, 2016, https://doi.org/10.1093/molbev/msw054). The phylogenetic tree was constructed using the neighbor joining method and by implying Poisson correction substitution model with uniform rates among the sites. In the tree, each enzyme is labeled with its unique identifier (ID) followed by the name of the organism; enzymes highlighted with a green circle are from the *U. maydis* strain 521, and proteins highlighted with a purple square are CDAs that were either biochemically characterized or studied by knock-out mutants, as mentioned in the text. Download FIG S2, PDF file, 2.5 MB. Copyright © 2021 Rizzi et al. 2021 Rizzi et al. https://creativecommons.org/licenses/by/4.0/ This content is distributed under the terms of the Creative Commons Attribution 4.0 International license.

In a phylogenetic tree of fungal CDAs (Fig. S2F), *U. maydis* Cda1, -2, -3, -5, and -6 belong to the “zygo/basidio-cluster.” Cda4 belongs to the “asco/basidio-cluster,” which all lack a GPI anchor (Fig. S2F). Cda7 is the first fungal CDA found outside these two clusters. However, homologous sequences exist in other fungi (Fig. S2F), establishing a new cluster of fungal CDAs whose members have not yet been functionally characterized.

To determine when the *cda* genes are expressed, we relied on published transcriptome sequencing (RNA-seq) data (29) (GEO database accession number GSE103876). This analysis revealed that *cda1* is highly expressed in axenic culture, *cda2* is expressed in axenic culture as well as during colonization, while *cda3*, *cda4*, and *cda7* were all induced during colonization (Fig. 2B and see Fig. S3A). *cda5* levels remained low throughout the life cycle, while transcripts of the presumed pseudogene *cda6* were negligible (Fig. 2B and Fig. S3A). In spores, *cda5* and *cda7* were expressed, while *cda3* was induced during spore germination (Fig. S3B).

10.1128/mBio.03419-20.3 FIG S3 Expression of *cda* genes during development of *U maydis*. (A) DESeq2-normalized read counts of *cda* genes at different time points of *Z. mays* infection with *U. maydis* FB1 × FB2. Original data from Fig. 2B. Data were obtained from reference 29. Means ± SDs are from three independent replicates. (B) Expression of *cda* genes from *U. maydis* in spores and during spore germination. Quantitative reverse transcription-PCR (qRT-PCR) was used to determine which *cda* genes are expressed in spores and during spore germination, because these stages were not represented in Lanver et al. (29) data set. qRT-PCR was performed with RNA extracted from leaves after 1 dpi with FB1 × FB2 as the reference and from spores collected from cobs infected with FB1 × FB2 as well as from spores after 9 and 18 h of germination. Expression of the *cda* genes was determined relative to the constitutively expressed *ppi* gene. Averages from three biological replicates are shown. Error bars indicate ± SDs. Significant differences determined by two-side unpaired Student’s *t* test. *, *P *≤ 0.05; **, *P *≤ 0.01; ***, *P *≤ 0.001. Download FIG S3, PDF file, 0.5 MB. Copyright © 2021 Rizzi et al. 2021 Rizzi et al. https://creativecommons.org/licenses/by/4.0/ This content is distributed under the terms of the Creative Commons Attribution 4.0 International license.

### Enzymatic activity of the seven putative Cda proteins.

To determine the enzymatic activity of the *U. maydis* CDA proteins, dicodon-optimized versions for all seven genes were generated for expression in Escherichia coli in such a way that the predicted signal peptide at the N-terminus was removed and replaced by an N-terminal thioredoxin domain followed by a sequence encoding a StrepII tag. At the C-terminus, the sequence encoding the signal for the GPI anchor was removed and replaced with a StrepII tag sequence. All seven *U. maydis* CDAs were successfully expressed (see Fig. S4A). Proteins were purified from the soluble fraction through affinity chromatography, quantified, and subsequently used for enzymatic analysis using pentaacetyl-chitopentaose (GlcNAc5 [A5]) as a substrate followed by ultrahigh-performance liquid chromatography electrospray ionization mass spectrometry (UHPLC-ESI-MS) to analyze the products. While Cda1, Cda2, Cda4, and Cda5 showed activity, we failed to detect enzymatic activity for Cda3, Cda6, and Cda7 (not shown). As this could indicate that the respective enzymes might need posttranslational modifications, we expressed the *cda3*, *cda6*, and *cda7* genes in *U. maydis* from the constitutive actin promoter. Western blot analysis revealed that Cda3 and Cda7 were expressed, while Cda6 was not detected (Fig. S4B). To enrich Cda3 and Cda7, culture supernatants were concentrated and tagged proteins were subsequently affinity purified with Strep-Tag beads and visualized by silver staining (Fig. S4C).

10.1128/mBio.03419-20.4 FIG S4 Purification and activity of *U maydis* CDA proteins. (A) Western blot analysis of purified codon-optimized StrepII-tagged *U. maydis* CDAs after heterologous expression in E. coli BL21(DE3). StrepII-tagged proteins were visualized after immunoblotting using horseradish peroxide (HRP)-conjugated Strep-Tactin and the chemiluminescent substrate luminol. Expected molecular weights for StrepII-tagged CDAs are 56 kDa for Cda1, 60 kDa for Cda2, 60 kDa for Cda3, 46 kDa for Cda4, 59 kDa for Cda5, 63 kDa for Cda6, and 69 kDa for Cda7. (B) Cda3, Cda6, and Cda7 were expressed constitutively in SG200 from the P_actin_ promoter after replacing the GPI anchor sequence with a StrepII tag. Cells containing single (SI) or multiple (MI) insertions of P_act_*cda3-strep*, P_act_*cda6-strep* or P_act_*cda7-strep* were grown in YEPSL. Total cell lysates were analyzed by Western blotting with anti-StrepII (αStrepII) antibody (top) and with anti-tubulin (αTub) antibody as a gel loading control (bottom). Expected molecular weights of StrepII-tagged proteins with signal peptide attached are 51 kDa for Cda3, 49 kDa for Cda6, and 57 kDa for Cda7. Unspecific bands are marked with an asterisk. (C) Silver staining of purified Cda3-StrepII (expected molecular weight, 49 kDa) and Cda7-StrepII (expected molecular weight, 55 kDa) after enrichment from the supernatants of strains analyzed in panel B. Proteins representing the purified secreted proteins are labeled with arrowheads. (D) Reaction products of *U. maydis* CDAs. UHPLC-ESI-MS base peak chromatograms of the products of *U. maydis* CDAs (Cda1, Cda2, Cda3, Cda4, Cda5, and Cda7) and *C. lindemuthianum* CDA (*Cl*CDA) on the pentaacetyl-chitopentaose substrate, A5 (AAAAA). BC marks peaks from buffer components carried along during the purification. A5-derived reaction products are marked with asterisks. UK1, A1D2, and A3 with monoisotopic values (*m/z*) of 578.3, 544.2, and 628.2, respectively, likely correspond to degradation products. Inserts represent enlargements of relevant peak areas. In the *Um*CDA4 products, trace amounts of A4D1 and A2D3 (ca. 5% and 7%, respectively) were also detected. (E and F) Dot activity gel of purified *U. maydis* CDA proteins. The in-gel assay was performed by incorporating glycol-chitin into an acrylamide gel and adding 5-μl drops of recombinant *Um*CDAs or *Cl*Cda as a positive control (Cda1, Cda2, Cda4, Cda5, and *Cl*Cda, 500 ng; Cda3, 615 ng; Cda7, 545 ng) in buffer (50 mM TEA, pH 7), followed by incubation overnight at 37°C and then washing the gel with water. The absence of dark spots upon chitin staining using calcofluor indicated the absence of chitinase activity (E), while the occurrence of dark spots after HNO_2_ depolymerization of chitosan indicated the presence of CDA activity (F). Download FIG S4, PDF file, 0.4 MB. Copyright © 2021 Rizzi et al. 2021 Rizzi et al. https://creativecommons.org/licenses/by/4.0/ This content is distributed under the terms of the Creative Commons Attribution 4.0 International license.

UHPLC-ESI-MS analysis of the enzymatic reaction products revealed that all six enzymes, Cda1, -2, -4, and -5 purified from E. coli as well as Cda3 and -7 purified from *U. maydis*, exhibited enzymatic activity with GlcNAc5 as the substrate, confirming their identity as CDA enzymes (Table 1). Under the conditions used, Cda4 completely converted the GlcNAc5 to the partially deacetylated chitosan pentamer GlcNAc3GlcN2 (A3D2) as the major reaction product, with traces of GlcNAc4GlcN1 (A4D1) and GlcNAc2GlcN3 (A2D3) (Table 1 and Fig. S4D). Cda2 converted approximately two-thirds of the substrate into the monodeacetylated product and one-third into the double-deacetylated product, while Cda1, Cda3, and Cda5 mainly produced monodeacetylated pentamers (Table 1). Cda7 was less active, leading to a single deacetylation in less than 10% of the substrate (Table 1). Like other fungal deacetylases (18, 20), recombinant Cda1, -2, -3, -4, and -5 were also active on chitin tetramer and hexamer as the substrates, while Cda7 was inactive on the tetramer but showed increased activity on the hexamer (Table 1). None of the six active CDAs of *U. maydis* displayed chitinase activity (Fig. S4E), while all showed CDA activity toward the polymeric soluble chitin derivative glycol-chitin (Fig. S4F).

**TABLE 1:** Activity of *U. maydis* CDAs on tetraacetyl-chitotetraose, pentaacetyl-chitopentaose, or hexaacetyl-chitohexaose as the substrates

CDA	Activity (%) on substrate:^*a*^														
	Tetraacetyl-chitotetraose				Pentaacetyl-chitopentaose					Hexaacetyl-chitohexaose					
	Substrate	Product			Substrate	Product				Substrate	Product				
	A4	A3D1	A2D2	A1D3	A5	A4D1	A3D2	A2D3	A1D4	A6	A5D1	A4D2	A3D3	A2D4	A1D5
Substrate	100 ± 0	ND^*b*^	ND	ND	100 ± 0	ND	ND	ND	ND	100 ± 0	ND	ND	ND	ND	ND
*Cl*Cda^*c*^^,^^*d*^	ND	ND	45 ± 5	55 ± 5	ND	ND	ND	28 ± 2	72 ± 2	ND	ND	3 ± 5	9 ± 8	82 ± 8	7 ± 11
*Um*Cda1^*d*^	90 ± 1	10 ± 1	ND	ND	55 ± 4	39 ± 4	6 ± 0	ND	ND	40 ± 1	30 ± 1	26 ± 2	3 ± 0	ND	ND
*Um*Cda2^*d*^	52 ± 2	48 ± 2	ND	ND	6 ± 1	60 ± 1	34 ± 1	ND	ND	2 ± 1	11 ± 0	76 ± 1	12 ± 0	ND	ND
*Um*Cda3^*e*^	88 ± 1	12 ± 1	ND	ND	57 ± 6	33 ± 3	10 ± 9	ND	ND	20 ± 2	35 ± 4	38 ± 1	7 ± 6	ND	ND
*Um*Cda4^*d*^	ND	78 ± 1	22 ± 1	ND	ND	7 ± 0	88 ± 0	5 ± 1	ND	ND	3 ± 2	14 ± 0	73 ± 2	10 ± 0	ND
*Um*Cda5^*d*^	88 ± 0	12 ± 0	ND	ND	56 ± 4	42 ± 3	1 ± 3	ND	ND	11 ± 1	56 ± 2	33 ± 1	ND	ND	ND
*Um*Cda7^*e*^	100 ± 0	ND	ND	ND	93 ± 0	7 ± 0	ND	ND	ND	85 ± 1	15 ± 1	ND	ND	ND	ND

a Mean value ± SD (*n* = 3 independent enzymatic reactions). Substrates are as follows: A4, tetraacetyl-chitotetraose; A5, pentaacetyl-chitopentaose; A6, hexaacetyl-chitohexaose.

b ND, not detectable.

c *Colletotrichum lindemuthianum* CDA, purified from E. coli used as positive control.

d Recombinant protein purified from E. coli.

e Protein purified from *U. maydis* culture supernatant.

### Phenotype of strains carrying single *cda* gene mutations.

**FIG 3:**
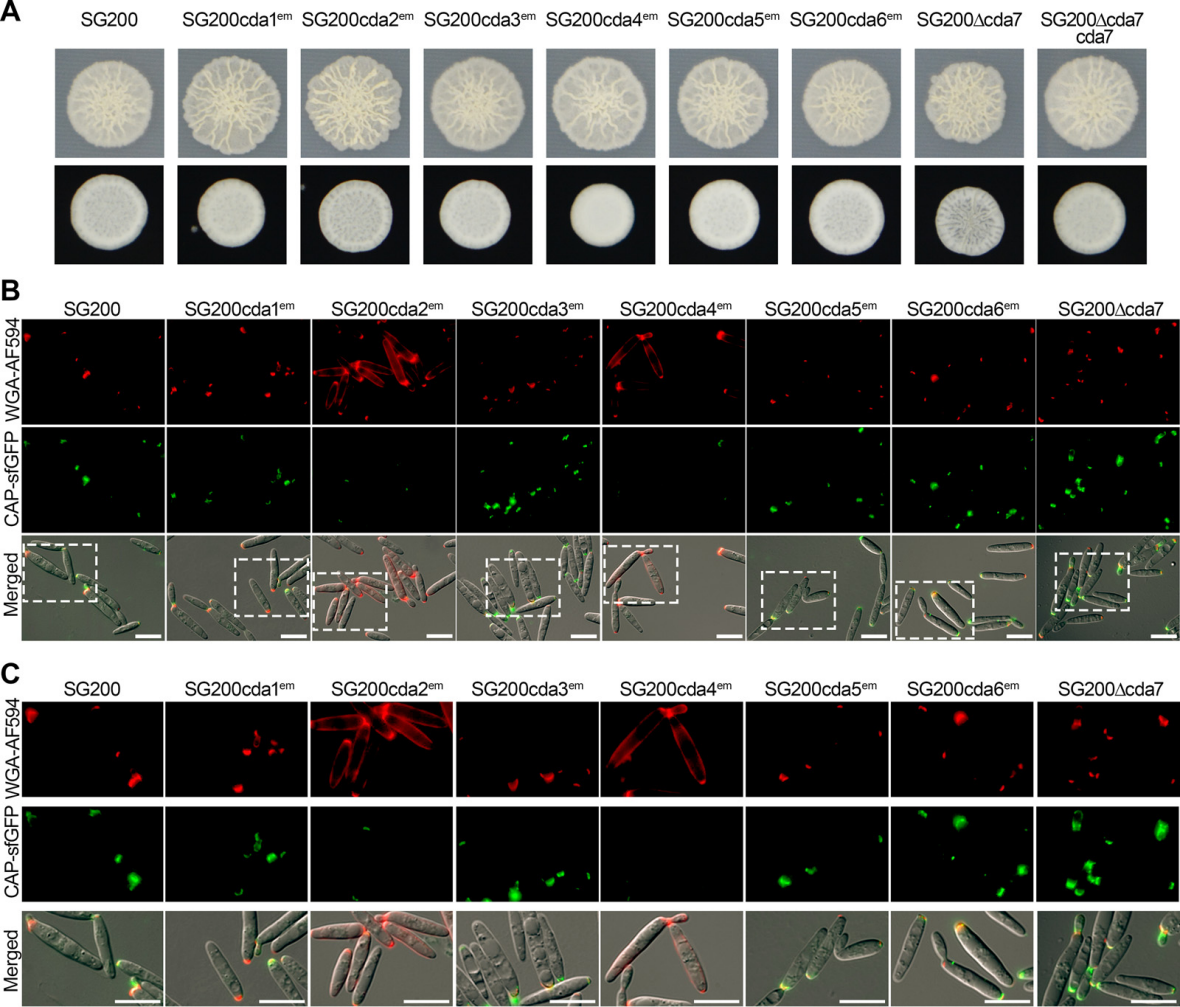
Colony morphology and chitin and chitosan staining of strains lacking single *cda* genes. (A) Cultures of the indicated *U. maydis* strains were spotted onto PD-agar for growth as budding cells (top) and on PD-charcoal for growth as cells producing aerial filaments (bottom). Pictures were taken after 2 days of incubation (top) and 1 day of incubation (bottom). Representative pictures from three independent experiments are shown. (B) Budding cells in exponential phase were stained with WGA-AF594 to detect chitin (red; top) or CAP-sfGFP to detect chitosan (green; middle); at the bottom, channels are merged with the bright-field channel. Scale bars, 10 μm. Representative pictures from three experiments are shown. (C) Enlargements of the stippled boxes marked in panel B. Scale bars, 10 μm.

**FIG 4:**
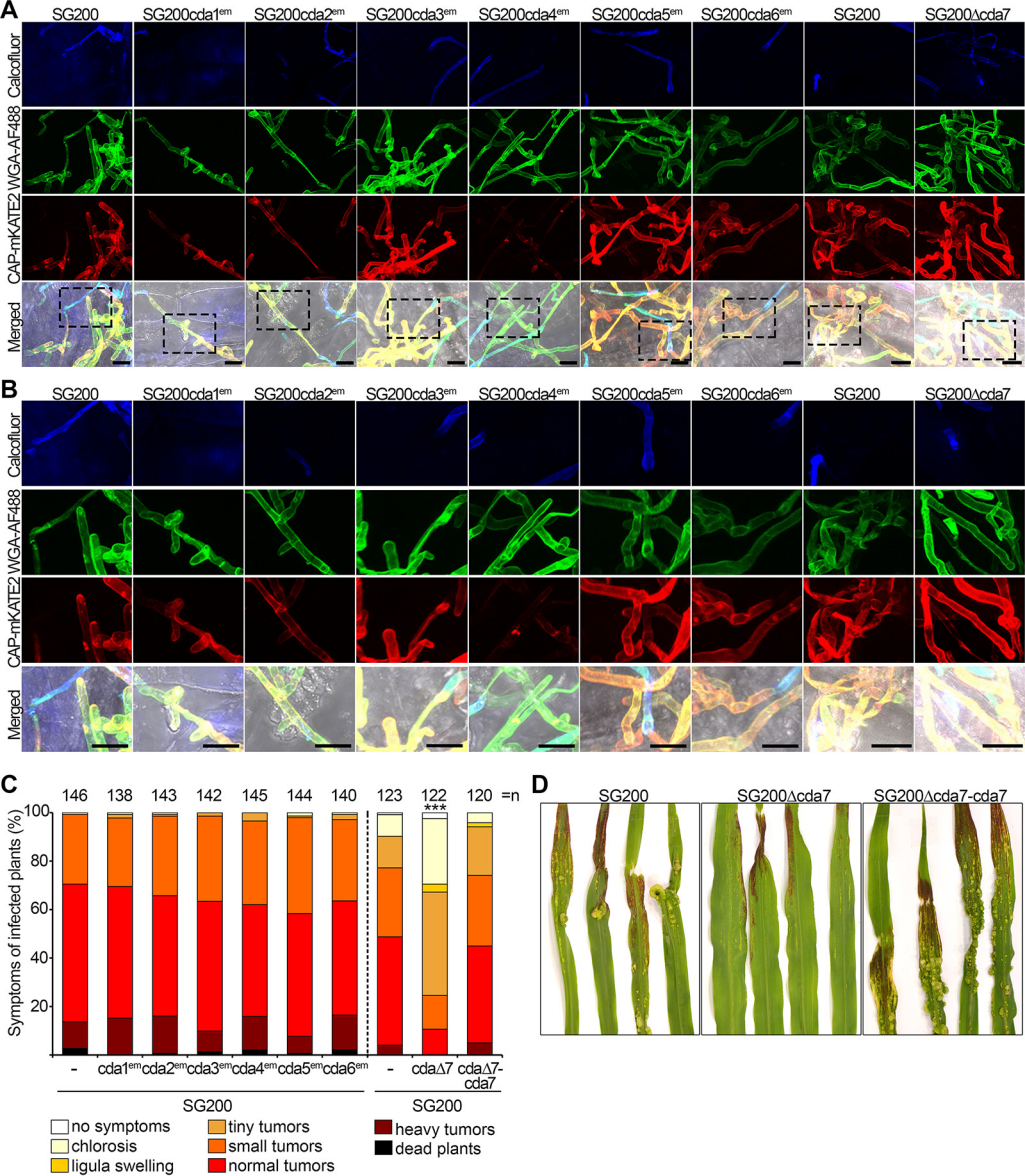
Chitin and chitosan staining of biotrophic hyphae and virulence of single *cda* mutant strains. (A) Staining of hyphae during colonization at 2 dpi. Hyphae of the indicated strains on the surface of the plant leaf are stained with calcofluor (blue; top), chitin is visualized by WGA-AF488 staining (green; second row), and chitosan with CAP-mKATE2 staining (red; third row). In the bottom row, channels are merged with the bright-field channel. Samples were observed by confocal microscopy, and all images are projections of multiples z-stacks. Scale bars, 10 μm. Representative pictures from three independent experiments are shown. (B) Enlargements of the stippled boxes marked in panel A. Scale bars, 10 μm. (C) Virulence assay for the strains shown in panel A as well as the complementation strain for Δ*cda7*, SG200 Δ*cda7*-*cda7*. Disease symptoms were scored at 12 dpi according to severity using the color code at the bottom. Three independent experiments were performed, and average values are expressed as a percentage of the total number of infected plants (*n*), which is given at the top of each column. Significant differences (Games-Howell test) in virulence compared with SG200 are indicated. ***, *P* < 0.001. (D) Macroscopic symptoms of plant leaves infected with the indicated strains at 12 dpi.

To study the function of individual *cda* genes, we used CRISPR-Cas9 technology (35) to disrupt *cda1*, -*2*, -*3*, -*4*, -*5*, and -*6* in strain SG200. *cda7* was deleted by conventional gene replacement technology (36). Initially, in order to minimize possible off-targets effects, at least three independently derived mutants were generated, compared for growth, colony morphology, and filamentation on charcoal plates, and tested for virulence. Without exception, mutant phenotypes for the same gene(s) were comparable, and consequently, one of each of these mutants was chosen for subsequent analyses. When individual mutants in the seven genes were compared, we did not observe significant differences in growth and in colony morphology (see Fig. S5A and B and Fig. 3A). With respect to filamentation on potato dextrose (PD)-charcoal plates, only the *cda7* mutant showed consistently weaker filamentation, and this phenotype was complemented by introducing *cda7* in single copy in the mutant strain (Fig. 3A). As *cda3* is induced during spore germination, we tested whether spore viability and germination were affected in the *cda3* mutant compared to that in SG200. While spore viability was low in general, presumably because SG200 is a mononuclear haploid strain, significant differences in spore viability (0.08% ± 0.05% for SG200 and 0.83% ± 0.79% for the *cda3* mutant) and cell morphology during spore germination (Fig. S5C) were not detected. Budding cells of the *cda2* mutant showed a significant reduction in length, while the other single mutants were not significantly altered in cell morphology (Fig. S5D). Mutant sensitivity toward stressors was largely unaffected, except that the *cda2* mutant appeared more susceptible to cell wall stressors (Fig. S5A). The chitin and chitosan staining patterns of *cda1*, -*3*, -*5*, -*6*, and -*7* mutants in culture were comparable to that of SG200 (Fig. 3B and C). In *cda2* and *cda4* mutants, chitosan staining of budding cells was decreased and chitin staining of the cell body was increased (Fig. 3B and C). In biotrophic hyphae of *cda4* mutants, chitosan staining was strongly reduced, and *cda2* mutants also showed somewhat reduced chitosan accumulation; the staining patterns of hyphae of the other mutants were comparable to that of SG200 (Fig. 4A and B and Fig. S5E). Virulence was only reduced in the *cda7* mutant, and this defect could be fully complemented (Fig. 4C and D).

10.1128/mBio.03419-20.5 FIG S5 Characterization of mutant strains lacking single *cda* genes. (A) Stress sensitivity of SG200 strains lacking single *cda* genes and the complemented strain SG200 Δ*cda7*-*cda7*. Serial 10-fold dilutions of cultures adjusted to an OD_600_ of 1.0 were spotted onto complete medium supplemented with 1% glucose in the absence of stressors (CM) or in the presence of stressors calcofluor, Congo red, NaCl, sorbitol, or H_2_O_2_ or in PD agar in the absence (PD) or presence of the stressor caffeine or SDS. The plates were incubated at 28°C and pictures were taken after 2 days for CM and PD, caffeine, and SDS, 3 days for the plates containing calcofluor, Congo red, or 1.5 mM H_2_O_2_, and 4 days for the plates containing NaCl, sorbitol, or 3 mM H_2_O_2_. (B) Morphology of colonies of single *cda* mutant strains. Microscopic pictures of single colonies of the indicated strains all grown on the same CM plate. Scale bars, 1 mm. (C) Spore germination of SG200 and SG200 *cda3*^em^. Microscopy was performed 48 h after spotting the spores onto a PD agar layer on microscopy slides and incubating at 28°C. Representative pictures are shown. Germinated spores are marked with arrowheads. Scale bars, 10 μm. (D) Width (left) and length (right) of budding cells of mutant strains lacking single *cda* genes. Three independent replicates each comprising 100 cell measurements were performed and are displayed by box plot representation; the mean value is represented with an “x” inside the box. Significant differences were determined with respect to SG200 by one-way analysis of variance (ANOVA) and Duncan multiple-range test. ***, *P ≤ *0.001. (E) Quantification of relative fluorescence intensity of CAP-mKATE2 chitosan staining of biotrophic hyphae of SG200 and strains lacking single *cda* genes at 2 dpi. The average fluorescence intensity in a cross section 5 μm from the hypha tip was measured and normalized to the average fluorescence intensity of SG200 in the same experiment, which was set to 100. The averages from four biological replicates are shown. Numbers indicate the total number of hyphae analyzed per strain. Error bars represent ± SDs. Significant differences were determined by two-side unpaired Student’s *t* test compared to SG200. *, *P ≤ *0.05; ***, *P ≤ *0.001). (F) Quantification of appressorium formation and successful penetration in the indicated strains with the help of promoters induced in appressoria (AM1 marker) and during penetration (PM marker) (S. Krombach. Philipps University, Marburg, Hesse, Germany. 2016, https://doi.org/10.17192/z2017.0051). For the quantification, infected maize seedlings were analyzed at 16 h postinfection by confocal microscopy. Appressorium formation was determined as the number of filaments expressing the AM1 marker relative to the total number of filaments stained with calcofluor (left), and penetration efficiency was determined as number of filaments expressing the PM marker relative to the number of filaments expressing the AM1 marker (middle). The percentage of appressoria impaired in penetration associated with a plant defense response was determined as the number of filaments expressing the AM1 marker but failing in expression of the PM marker that were associated with calcofluor staining underneath the appressoria relative to the total number of filaments expressing the AM1 marker but not the PM marker (right). In each biological replicate, 15 leaf areas from three leaves were evaluated per strain. Averages from six biological replicates are presented. Error bars indicate ± SDs. Significant differences were determined by two-side unpaired Student’s *t* test compared to SG200. *, *P ≤ *0.05; **, *P ≤ *0.01. Download FIG S5, PDF file, 0.9 MB. Copyright © 2021 Rizzi et al. 2021 Rizzi et al. https://creativecommons.org/licenses/by/4.0/ This content is distributed under the terms of the Creative Commons Attribution 4.0 International license.

To elucidate at which stage of development the *cda7* mutant is affected, we determined appressorium formation as well as penetration efficiency. While appressorium formation was not altered relative to that for SG200, penetration was significantly reduced in the *cda7* mutant and was restored in the complemented strain (Fig. S5F). When cells that expressed the appressorial marker but failed to penetrate were assayed for defense responses, no significant differences between the *cda7* mutant, SG200, and complementation strain were detected (Fig. S5F). A compilation of all single mutant phenotypes is given in Fig. S6.

10.1128/mBio.03419-20.6 FIG S6 Scheme listing the phenotypes of the different *U. maydis cda* mutants generated for this study. Blue color intensity indicates the degree of reduction in mutants compared to that in the wild type, intensity of orange indicates the degree by which a certain phenotype is increased in certain mutants, and n.a. are conditions not analyzed. Download FIG S6, PDF file, 0.5 MB. Copyright © 2021 Rizzi et al. 2021 Rizzi et al. https://creativecommons.org/licenses/by/4.0/ This content is distributed under the terms of the Creative Commons Attribution 4.0 International license.

### Phenotype of mutants lacking up to six *cda* genes.

**FIG 5:**
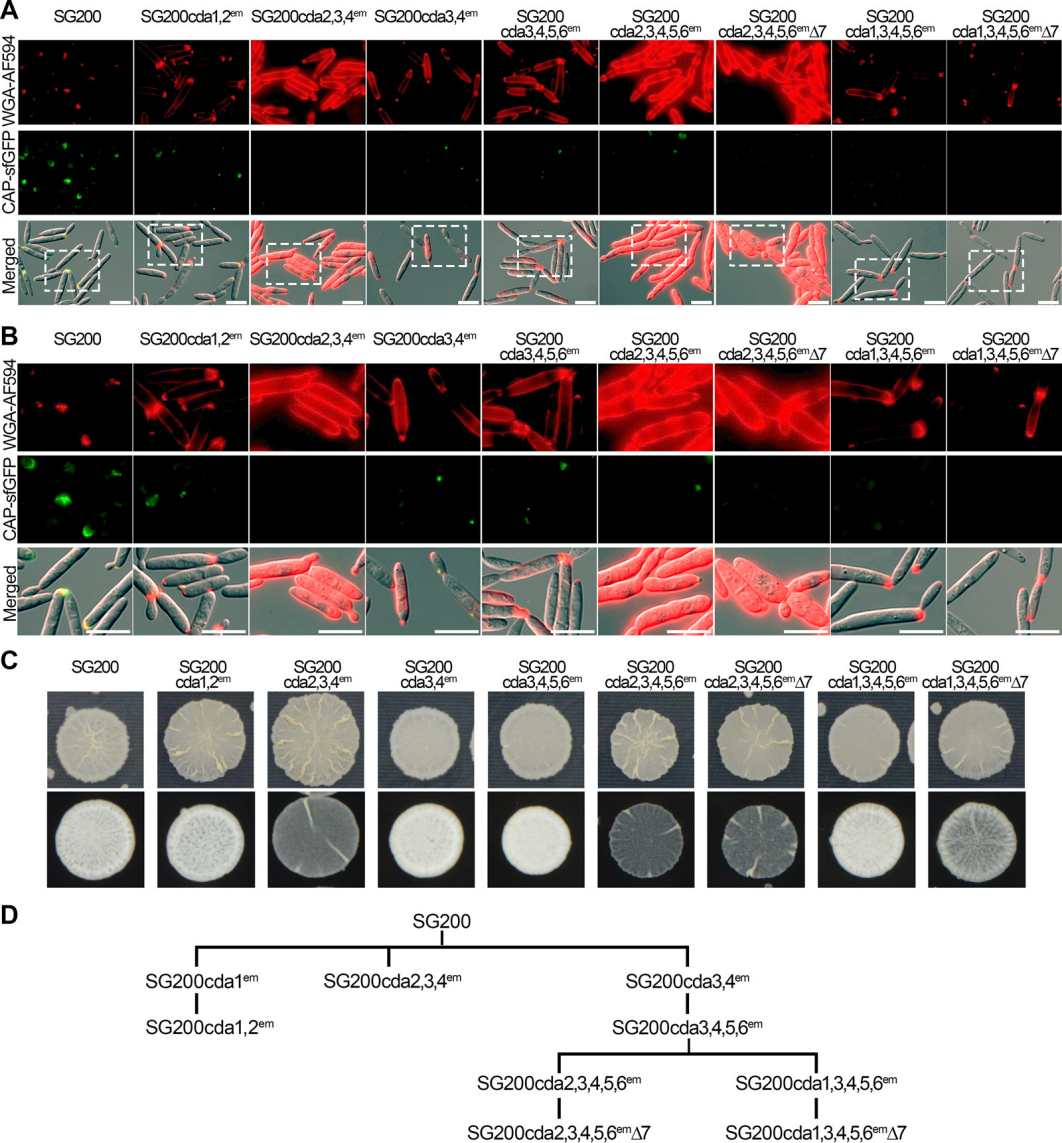
Chitin and chitosan staining and colony morphology of strains lacking multiple *cda* genes. (A) Budding cells in exponential phase of the indicated strains were stained with WGA-AF594 to detect chitin (red; top) and with CAP-sfGFP to detect chitosan (green; middle); at the bottom, channels are merged with the bright-field channel. Scale bars, 10 μm. Representative pictures from two or more experiments are shown. (B) Enlargements of the stippled boxes marked in panel A. Scale bars, 10 μm. (C) Cultures of the indicated *U. maydis* strains were spotted onto PD-agar for growth as budding cells (top) and on PD-charcoal for growth as cells producing aerial filaments (bottom). Pictures were taken after 2 days of incubation (top) and 1 day of incubation (bottom). (D) Pedigree of strains containing multiple inactivated *cda* genes.

Next, we considered functional redundancy between the CDAs and decided to inactivate several *cda* genes simultaneously based on expression profiles (Fig. 2B). When *cda1* and *cda2*, the genes most highly expressed in culture, were simultaneously inactivated, sensitivity to the cell wall stressor calcofluor and the cell diameter increased relative to that in the *cda2* mutant, and cells became shorter (see Fig. S7A and B). With respect to chitin and chitosan staining, the double mutant was comparable to the *cda2* mutant (Fig. 5A and B). In a *cda3*,*4* mutant, where the genes most highly expressed during colonization were coinactivated, the chitin and chitosan staining were comparable to that for the *cda4* mutant (Fig. 5A and B). However, in the triple *cda2*,*3*,*4* mutant, where the three most highly expressed genes during colonization were inactivated, filamentation was affected, stress sensitivity was altered, and chitosan levels were decreased further, while chitin levels were increased relative to that in the double mutant (Fig. 5A to C and Fig. S7A). SG200 *cda3,4*^em^ served as the parent for the inactivation of additional *cda* genes (Fig. 5D). Neither a quadruple mutant lacking *cda3*,*4*,*5*,*6* nor a quintuple mutant lacking *cda1*,*3*,*4*,*5*,*6* showed morphological alterations, but both showed slightly increased staining for chitin (Fig. 5 and Fig. S7A to C). However, when *cda2* was inactivated in combination with *cda3*,*4*,*5*,*6*, the formation of aerial hyphae was severely affected (Fig. 5C and Fig. S7D), the mutant showed higher sensitivity to cell wall stress and displayed higher resistance to H_2_O_2_, and the colony diameter was decreased (Fig. S7A and C), suggesting a slight growth defect. In addition, the *cda2*,*3*,*4*,*5*,*6* mutant displayed a cell separation defect, an increase in cell diameter, a strong reduction of cell length, and strongly increased chitin staining without an additional reduction in chitosan staining compared to that in the *cda3*,*4*,*5*,*6* mutant (Fig. 5A and B and Fig. S7B). When *cda7* was additionally deleted in the quintuple mutant, the phenotypes were intensified, and most prominently, the cell diameter increased further and cells appeared rounder (Fig. 5A and B and Fig. S7A to D). Reduced aerial hypha formation was also seen in the *cda1*,*3*,*4*,*5*,*6*,*7* mutant, but in this case, the filamentation defect was less severe than what was seen in the *cda2*,*3*,*4*,*5*,*6* mutant (Fig. 5C). Attempts to inactivate *cda1* and *cda2* simultaneously in a *cda3*,*4*,*5*,*6* mutant or to inactivate *cda1* in the strain lacking *cda2*,*3*,*4*,*5*,*6*,*7* or *cda2* in the strain lacking *cda1*,*3*,*4*,*5*,*6*,*7* were unsuccessful. In the latter two cases, we obtained deletion mutants lacking between 3 and 60 nucleotides (Fig. S7E), but we failed to obtain frameshift mutations.

10.1128/mBio.03419-20.7 FIG S7 Characterization of mutant strains lacking multiple *cda* genes. (A) Stress sensitivity of the indicated strains. Serial 10-fold dilutions of cultures adjusted to an OD_600_ of 1.0 were spotted onto complete medium supplemented with 1% glucose in the absence of stressors (CM) or in the presence of stressors calcofluor, Congo red, NaCl, sorbitol, or H_2_O_2_ or in PD agar in the absence (PD) or presence of the stressor caffeine or SDS. The plates were incubated at 28°C, and pictures were taken after 2 days for CM, PD, caffeine, and SDS, 3 days for the plate containing calcofluor, Congo red, or 1.5 mM H_2_O_2_, and 4 days for the plates containing NaCl, sorbitol, or 3 mM H_2_O_2_. (B) Width (top) and length (bottom) of strains lacking multiple *cda* genes. Three independent replicates each comprising 100 cell measurements were performed and are displayed by box plot representation; the mean value is represented with an “x” inside the box. Significant differences were determined with respect to SG200 by one-way ANOVA and Duncan multiple-range test. *, *P ≤ *0.001. (C) Morphology of colonies of multiple *cda* mutant strains. Microscopic pictures of single colonies of the indicated strains all grown on the same CM plate. Scale bars, 1 mm. (D) Microscopic pictures of edges of colonies of SG200, SG200 *cda2,3,4,5,6*^em^, and SG200 *cda2,3,4,5,6*^em^Δ*7*; 10 μl of the cultures adjusted to an OD_600_ of 1.0 was spotted onto a PD-charcoal plate and photographed after 2 days of incubation. (E) Inactivation of *cda2* in SG200 *cda1,3,4,5,6*^em^Δ*7* leads to in-frame mutations. The top row shows the nucleotide sequence of *cda2* between nucleotides 61 and 180. The target for the sgRNA is indicated in green, protospacer-adjacent motif (PAM) sequence is indicated in blue, and the expected cleavage site is indicated by an arrowhead. The 3′ end of the sequence encoding the signal peptide is indicated in pink. In total, 96 mutants were analyzed, and 15 representative sequences are shown. The deleted parts are given in orange. Numbers on the right indicate how many nucleotides were deleted. (F) Adherence of the hyphae to the leaf surface. Adherence was analyzed in leaves infected with the indicated strains 12 h postinfection. The leaf samples were stained with calcofluor and observed by confocal microscopy either before washing or after washing in water containing 0.1% Tween 20. Cells which have disappeared after the washing step are marked with white arrowheads. Scale bars, 50 μm. (G) Quantification of appressorium formation and successful penetration in the indicated strains expressing the appressorial marker AM1 and the penetration marker PM. For the quantification, infected maize seedlings were analyzed at 16 h postinfection by confocal microscopy. Appressorium formation was determined as the number of filaments expressing the AM1 marker relative to the total number of filaments stained with calcofluor (left) and penetration efficiency as number of filaments expressing the PM marker relative to the number of filaments expressing the AM1 marker (middle). Defense responses associated with calcofluor staining underneath the appressoria were determined by identifying appressoria impaired in penetration (filaments expressing the AM1 marker but not expressing the PM marker) and relating this in percentage to the total number of appressoria impaired in penetration (right). In each biological replicate, 15 leaf areas from three leaves were evaluated per strain. Averages from four biological replicates are presented. Error bars indicate ± SDs. Significant differences were determined by two-side unpaired Student’s *t* test compared to SG200. **, *P ≤ *0.01, ***, *P ≤ *0.001. (H) Relative fungal biomass was determined by qPCR. For this, genomic DNA was prepared at 0.5, 1, 2, 4, 6, and 8 dpi from maize leaves infected with the indicated strains. The fungal gene *ppi* and the plant gene *gapdh* were used for estimating relative fungal biomass. SG200 biomass at 0.5 dpi was set to 1. Average values from three biological replicates are shown. Error bars indicate ± SDs. Significance of differences between mutant strains and SG200 at each time point was calculated by Student’s *t* test. *, *P ≤ *0.05; **, *P ≤ *0.01. (I) Percentage of appressoria eliciting callose deposition. At 2 dpi, the appressoria on the leaf surface of the indicated strains were identified after calcofluor staining, and callose deposition was visualized by costaining with aniline blue. The percentage of appressoria that induced callose accumulation was determined. Five leaf areas from two leaves per strain were analyzed, and between 92 and 283 appressoria for SG200 and between 20 and 58 appressoria for SG200 *cda2,3,4,5,6*^em^ were studied. Average values from five biological replicates are shown. Error bars indicate ± SDs. Significance of difference was calculated by Student’s *t* test. **, *P ≤ *0.01. (J) Chitinase treatment on filaments of selected *cda* mutants. The strains indicated on the left were grown for 24 h on PD-charcoal plates to induce filamentation and subsequently removed, treated for 1 h with chitinase, and observed by microscopy. Representative pictures are shown, and the experiment was repeated three times with similar results. Scale bars, 50 μm. Download FIG S7, PDF file, 1.2 MB. Copyright © 2021 Rizzi et al. 2021 Rizzi et al. https://creativecommons.org/licenses/by/4.0/ This content is distributed under the terms of the Creative Commons Attribution 4.0 International license.

**FIG 6:**
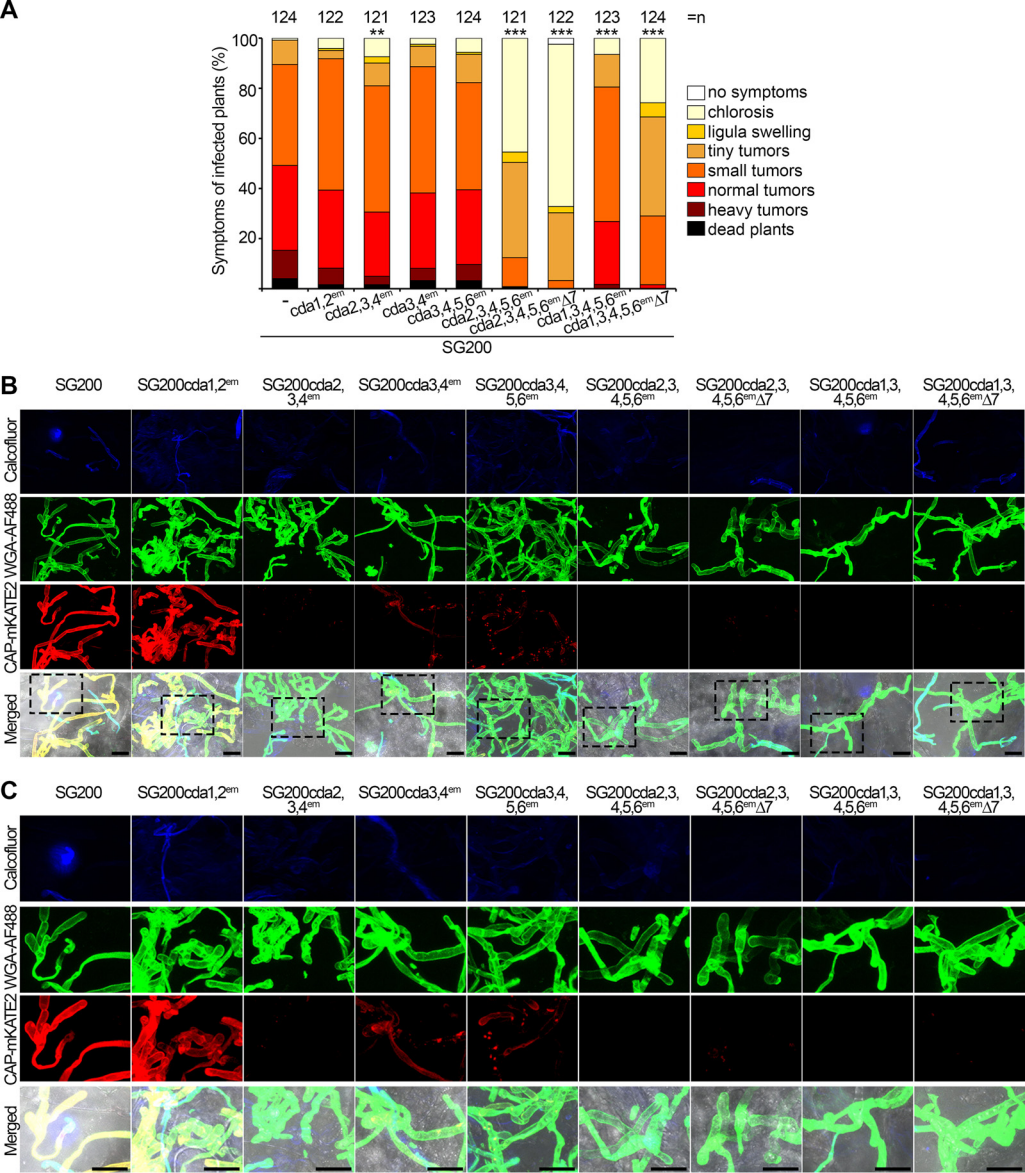
Virulence and chitin and chitosan staining of biotrophic hyphae of multiple *cda* gene mutants. (A) Virulence assay for the indicated strains. Symptoms were scored at 12 dpi according to severity using the color code on the right. Three independent experiments were performed, and average values are expressed as a percentage of the total number of infected plants (*n*), which is given at the top of each column. Significant differences (Games-Howell test) in virulence compared with SG200 are indicated. **, *P* < 0.005; ***, *P* < 0.001. (B) Staining of hyphae of the indicated mutants during colonization at 2 dpi. Hyphae on the surface of the plant leaf are stained with calcofluor (blue; top), chitin is visualized by WGA-AF488 staining (green; second row), and chitosan with CAP-mKATE2 staining (red; third row). In the bottom row, all channels are merged with the bright-field channel. Samples were observed by confocal microscopy, and all images are projections of multiple z-stacks. Scale bars, 10 μm. Representative pictures from at least two independent experiments are shown. (C) Enlargements of the stippled boxes marked in panel B. Scale bars, 10 μm.

With respect to virulence, neither the *cda1*,*2*, the *cda3*,*4*, nor the *cda3*,*4*,*5*,*6* mutant was significantly altered (Fig. 6A). The *cda2*,*3*,*4* mutant, which was severely affected in aerial hypha formation on PD-charcoal plates, was unexpectedly only slightly affected in virulence (Fig. 6A). The strain in which *cda2*,*3*,*4*,*5*,*6* were inactivated showed a strong reduction in virulence which became more severe when *cda7* was also inactivated (Fig. 6A). Compared to that for SG200, the *cda2*,*3*,*4*,*5*,*6* mutant showed reduced adherence to the leaf surface and reduced appressorium formation as well as reduced penetration efficiency (Fig. S7F and G). Compared to that for the *cda3*,*4*,*5*,*6* mutant, which is not affected in virulence, the *cda1*,*3*,*4*,*5*,*6* mutant showed reduced virulence, which became more severe when *cda7* was additionally deleted (Fig. 6A). All multiple mutants which were strongly reduced in virulence (Fig. 6A) showed significantly less chitosan staining in biotrophic hyphae at 2 dpi (Fig. 6B and C). However, strains lacking *cda3*,*4* or lacking *cda3*,*4*,*5*,*6* which were not impaired in virulence also showed reduced chitosan staining, though slightly less severe than in the strains with reduced virulence. The strains with strong virulence defects (SG200 *cda2,3,4,5,6*^em^ and SG200 *cda2,3,4,5,6*^em^Δ*7*) showed significant reductions in fungal biomass already at 2 dpi, while SG200 *cda1,3,4,5,6*^em^Δ*7* was reduced in biomass starting at 6 dpi; in infections with SG200 Δ*cda7*, biomass reduction became apparent only after 8 dpi (Fig. S7H).

To elucidate the basis for the strong virulence reduction of SG200 *cda2,3,4,5,6*^em^, we quantified cellular defense responses. While there were no significant differences between the mutant and SG200 with respect to calcofluor staining underneath appressoria, aniline blue staining for callose deposition revealed a significantly higher percentage of mutant appressoria which elicited callose deposition (Fig. S7G and I). To ascertain whether chitinase sensitivity and virulence are linked, we treated mutant filaments with chitinase in the presence of sorbitol as an osmotic stabilizer. While we observed slightly increased protoplastation in the single *cda7* mutant compared to that in SG200, the *cda1*,*3*,*4*,*5*,*6*,*7* mutant showed much stronger protoplastation; *cda2*,*3*,*4*,*5*,*6* and *cda2*,*3*,*4*,*5*,*6*,*7* mutants did not show this (Fig. S7J). A compilation of phenotypes detected in multiple *cda* mutants is given in Fig. S6.

### Phenotype of mutants lacking seven *cda* genes.

**FIG 7:**
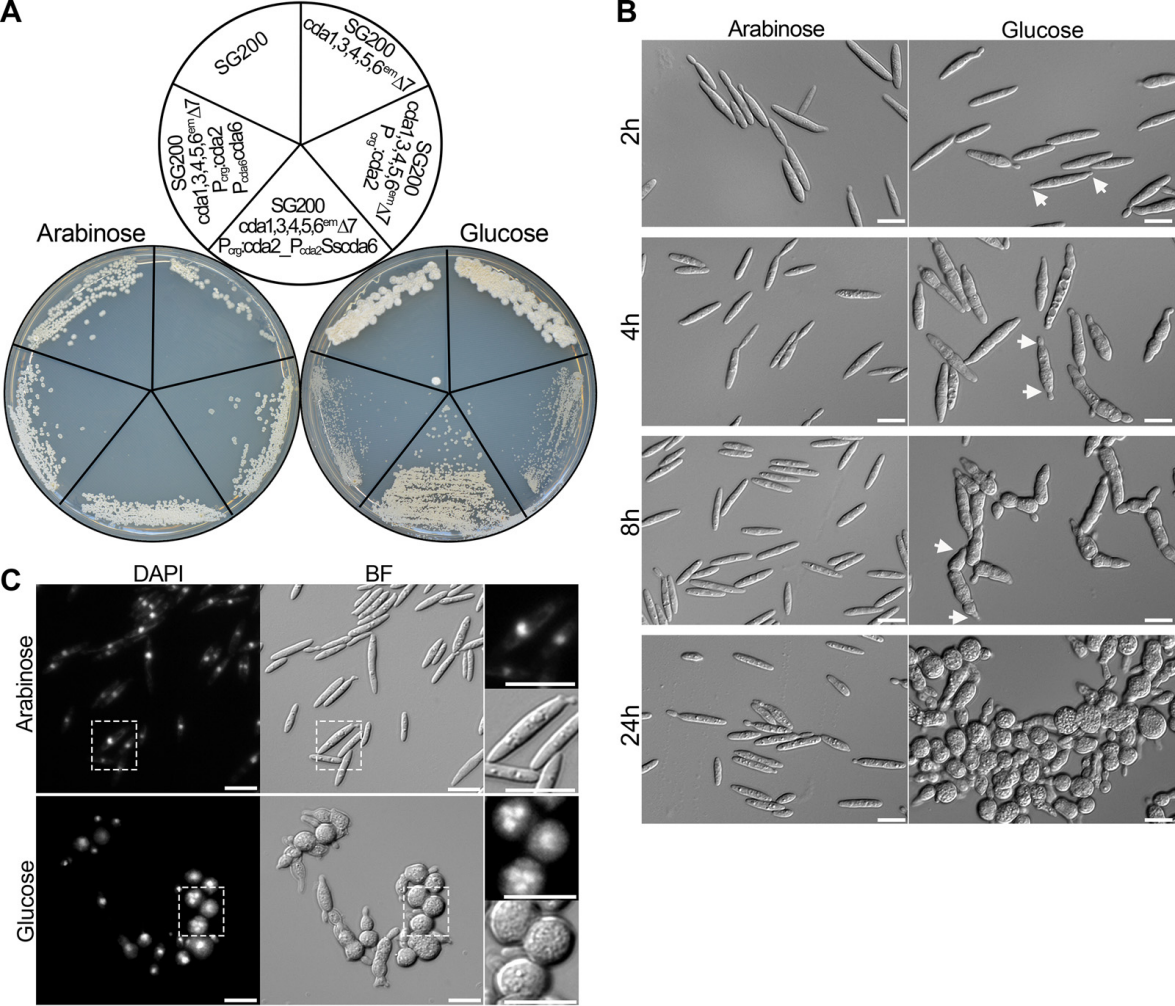
Viability of *U. maydis* strains lacking all *cda* genes. (A) Growth of strains indicated on top was assessed on CM-arabinose (left) and on CM-glucose (right). Plates were incubated for 4 days at 28°C. (B) Morphological changes in cell shape after the depletion of *cda2* in SG200 *cda1,3,4,5,6*^em^Δ*7*,P_crg_:*cda2*. Indicated strains were initially grown in liquid CM containing 1% arabinose and, after adjusting the OD_600_ to 0.2, shifted to liquid CM containing 1% arabinose as control (left) or to liquid CM containing 1% glucose, where *cda2* is successively depleted (right). The cultures were observed microscopically at 2, 4, 8, and 24 h postshift. White arrows show bipolar growth. (C) DAPI staining of SG200 *cda1,3,4,5,6*^em^Δ*7*,P_crg_:*cda2*, after 24 h of shift to liquid CM containing 1% arabinose as a control or liquid CM containing 1% glucose. Consecutive pictures were taken in one stack, and maximal projections are shown. DAPI and bright-field (BF) pictures are shown. On the right, enlarged pictures of the stippled areas are displayed.

To investigate whether our failure to generate a strain in which all seven *cda* genes are inactivated is due to lethality, we used strain SG200 *cda1,3,4,5,6*^em^Δ*7* to conditionally inactivate *cda2* by fusing the gene in locus to the *crg1* promoter (37). This promoter is active in arabinose-containing medium and is off in glucose-containing medium. On an arabinose-containing complete medium (CM) plate, SG200 *cda1,3,4,5,6*^em^Δ*7*,P_crg_:*cda2* showed normal growth (Fig. 7A), but on a glucose-containing CM plate, growth was strongly impaired (Fig. 7A). When the minute colonies which developed after 4 days of incubation were restreaked on a glucose-containing CM plate, we observed colonies heterogeneous in size (see Fig. S8A), presumably due to an accumulation of suppressor mutations.

10.1128/mBio.03419-20.8 FIG S8 Viability of *U. maydis* strains lacking all *cda* genes. (A) Six single colonies labeled a to f of SG200 *cda1,3,4,5,6*^em^Δ*7*,P_crg_:*cda2* from the CM plate containing 1% glucose shown in Fig. 7A were restreaked on CM containing 1% glucose and incubated for 4 days. Colonies of SG200 and SG200 *cda1,3,4,5,6*^em^Δ*7* were streaked as controls. The enlargement for area c shows colony heterogeneity indicative of the emergence of suppressor mutations. (B) Cell morphology of SG200 and SG200 *cda1,3,4,5,6*^em^Δ*7*. Indicated strains were initially grown in liquid CM containing 1% arabinose and after, adjusting the OD_600_ to 0.2, shifted liquid CM containing 1% arabinose as control (left). The cultures were observed microscopically at 2, 4, 8, and 24 h postshift. This assay serves as control for Fig. 7B. (B) Cell disintegration in SG200 *cda1,3,4,5,6*^em^Δ*7*,P_crg_:*cda2*. After 24 h in glucose-containing medium, disintegrating cells can be observed (white arrow). (D) Nuclei in SG200 *cda1,3,4,5,6*^em^Δ*7* which served as precursor for the depletion of Cda2. DAPI staining of the indicated strains 24 h after the shift to liquid CM medium containing 1% arabinose or CM containing 1% glucose. This figure serves as control for Fig. 7C. (E) Cell morphology of SG200 *cda1,3,4,5,6*^em^Δ*7*,P_crg_:*cda2* containing P_cda2_*Sscda6* in single or multiple copies after 24 h of growth in liquid CM containing 1% glucose. Emerging cigar-shaped cells whose length increased with copy number of P_cda2_*Sscda6* are indicated (white arrows). SG200 *cda1,3,4,5,6*^em^Δ*7*,P_crg_:*cda2* grown under the same conditions is shown as control. Download FIG S8, PDF file, 0.7 MB. Copyright © 2021 Rizzi et al. 2021 Rizzi et al. https://creativecommons.org/licenses/by/4.0/ This content is distributed under the terms of the Creative Commons Attribution 4.0 International license.

To study in detail what impairs growth, SG200 *cda1,3,4,5,6*^em^Δ*7*,P_crg_:*cda2* was shifted to glucose-containing liquid CM and cell fate was followed microscopically. The same strain grown in arabinose-containing CM served as a control. Already 2 h after the shift, the diameter of cells grown in glucose-containing medium increased, and cells showed stronger segmentation and often displayed bipolar growth (Fig. 7B). These phenotypes were enhanced at 4 h. After 8 h, the majority of cells had started to round up. After 24 h, rounded cells increased in diameter, often appeared in clusters, and some burst (Fig. 7B and Fig. S8B), illustrating that the depletion of *cda2* in the absence of the other CDAs is lethal. The same strain grown in arabinose-containing medium and the parental strain grown in glucose-containing medium did not show any of these alterations (Fig. 7B and Fig. S8C). DAPI (4′,6-diamidino-2-phenylindole) staining revealed that enlarged cells frequently contained several nuclei (Fig. 7C), while cells of the parental strain contained only a single nucleus (Fig. S8D).

The fact that an *U. maydis* strain with all seven *cda* genes inactivated is nonviable allowed us to test functionality of the presumed pseudogene *cda6*. To this end, the native *cda6* gene, including the upstream and downstream regions extending up to the neighboring genes, was introduced in SG200 *cda1,3,4,5,6*^em^Δ*7*,P_crg_:*cda2* in two copies. However, viability of the strain was not restored after growth on glucose-containing medium (Fig. 7A). We also inserted the gene orthologous to *cda6* from Sporisorium scitamineum (*Sscda6*) under the control of the *cda2* promoter in the *ip* locus of SG200 *cda1,3,4,5,6*^em^Δ*7*,P_crg_:*cda2*. When SG200 *cda1,3,4,5,6*^em^Δ*7*,P_crg_:*cda2*,P_cda2_:*Sscda6* was analyzed in glucose-containing CM, partial growth complementation was observed (Fig. 7A and Fig. S8E). This illustrates that *cda6* from *S. scitamineum* must be active and can at least partially rescue the lethality of the strain in which all seven *cda* genes are inactivated.

## DISCUSSION

In this study, we have functionally analyzed the full set of CDAs in a fungal plant pathogen. The *U. maydis cda* gene family consists of six genes encoding active enzymes and one pseudogene. All of these enzymes exhibited activity on the soluble polymeric substrate glycol-chitin and on soluble chitin oligomers, showing increasing activity with increasing oligomer size, as typically seen with most fungal CDAs (20).

Cda7 is the first member of a new group of fungal CDAs not recognized before. Homologous genes to *cda7* can be found in the genomes of other smut fungi and in rust fungi but also in ectomycorrhizal fungi and in mushroom-forming fungi such as Agaricus bisporus. Of the single mutants, only the strain lacking *cda7* exhibited reduced virulence; thus, it will be interesting to investigate the role of this new CDA family in the other basidiomycetes. *cda7* mutants display an early defect during penetration which is not fully consistent with biomass determinations, where we see a late defect in biomass accumulation in the mutant. We speculate that the expected small differences in early biomass caused by fewer successfully penetrating cells may not be detectable by quantitative PCR (qPCR). Whether *cda7* has a second function late during pathogenic development needs to be assessed in compatible mutants, because SG200 has defects in late proliferation (F. Fukada, personal communication). A possible involvement of *cda3* during spore germination, when the gene is induced, should also be reanalyzed with compatible haploid *cda3* mutant strains, because SG200 is strongly impaired in spore formation (F. Fukada, personal communication) and spore germination, as shown here.

Until now, the full set of *cda* genes has been deleted in Schizosaccharomyces pombe (38), Saccharomyces cerevisiae (39, 40), C. neoformans (41), and Aspergillus fumigatus (42). In all of these species, the mutants were viable and showed defects only under stress conditions. Our finding that, in *U. maydis*, at least one CDA is essential for fungal viability has not been observed previously in any fungus.

Of the *U. maydis* CDAs, Cda4 shows the highest enzymatic activity, the strongest induction after colonization, and resides in the cluster of fungal CDAs to which also *Vd*PDA1 from V. dahliae and Pst_13661 from *P. striiformis* belong, which all lack a GPI anchor and strongly contribute to virulence (14, 25). To our surprise, we were unable to detect a virulence defect in *cda4* mutants in which the continuous layer of chitosan surrounding biotrophic hyphae was strongly reduced. For this reason, we consider that protection from chitinases could be provided by effector proteins, which may either bind to hyphae or downregulate the synthesis of chitinases. This is supported by the observation that *U. maydis* induces several plant chitinases before fungal penetration, but the expression of these genes ceases upon plant colonization (43). Interestingly, several of these chitinase genes are again transcriptionally upregulated late in infection (29), suggesting a possible protective role of chitosan at later time points. The finding that we observe increased chitinase sensitivity in the multiple mutant lacking *cda1*,*3*,*4*,*5*,*6*,*7*, which also has a defect in late biomass accumulation, lends supports to such a scenario.

We observed virulence defects in the mutants lacking *cda2*,*3*,*4*, *cda2*,*3*,*4*,*5*,*6*, and *cda2*,*3*,*4*,*5*,*6*,*7*. These three mutants have defects already during filamentation on charcoal plates and show a strong chitin accumulation in budding cells. As an increased chitin content might enhance the rigidity of the cell wall due to crystallization of chitin fibrils, chitosan could provide for an increased flexibility of the cell wall (44), which might be beneficial for the morphological transition from budding cells to hyphae. We consider it unlikely that the reduced aerial hypha formation is the primary virulence defect, because the *cda2*,*3*,*4* mutant is almost as defective in aerial hypha production as the *cda2*,*3*,*4*,*5*,*6* mutant but shows only a moderate virulence defect. While the *cda2*,*3*,*4*,*5*,*6* mutant switched to filamentous growth on the leaf surface, these hyphae appeared shorter than wild-type hyphae and displayed reduced adherence. In addition, they showed defects in appressorium formation and penetration efficiency, and the mutant induced more callose deposition and showed a reduction in fungal biomass compared to that of SG200. This makes it likely that the deacetylation of chitin is important during different stages of biotrophic development.

In the following, we will discuss whether there is redundancy with respect to some of the functions affected by the different CDA proteins. To simplify this, we refer to Fig. S6 in the supplemental material. The proteins responsible for the accumulation of chitosan in budding cells are mainly Cda2 and Cda4. Related to this, *cda2* and *cda4* single mutants display an increased amount of chitin in the entire cell body, which increases even more when both genes are inactivated and when *cda3* and *cda5* are also inactivated. This additive phenotype likely reflects redundancy between these four CDAs, and this is specific; for example, we did not observe an additional increase when *cda7* was coinactivated. With respect to cell shape, mutants only expressing *cda1* or *cda2* are informative because the mutant expressing *cda1* is altered in cell shape, has a wider diameter, is shorter in length, and shows a cell separation defect, while the mutant still harboring the *cda2* gene appears unaffected in cell shape. This indicates that *cda2* is required for cell shape maintenance while *cda1* is not. Budding cell viability can be supported by Cda1 and Cda2; we cannot comment on whether the other *cda* genes could carry out this function, because respective strains expressing only *cda3*, *cda4*, *cda5*, or *cda7* have not been generated. However, based on our finding that we were unable to inactivate *cda2* and *cda1* simultaneously in a mutant harboring *cda7*, we would like to argue that the essential function cannot be carried out by Cda7. With respect to filamentation, there is a slight reduction in single *cda7* mutants, while this phenotype is strongly intensified when *cda2*,*3*,*4*,*5*,*6* are coinactivated. This is in line with observing a strong filamentation defect already in the *cda2*,*3*,*4* mutant. It is also apparent that the presence of *cda2* alone supports filamentation, which recovers to wild-type levels when *cda7* is also present. This shows on the one hand redundancy, but also specificity. Regarding the chitosan layer in biotrophic hyphae, Cda4 is mainly responsible. With respect to virulence, the *cda7* mutant shows a virulence defect that is obviously not fully complemented by the other *cda* genes. When the *cda7* gene is coinactivated with *cda2*,*3*,*4*,*5*,*6* or *cda1*,*3*,*4*,*5*,*6*, the virulence defect increased either due to redundancy or because the different Cda proteins affect different steps of biotrophic development. The finding that we also observe a virulence phenotype in a *cda2*,*3*,*4*,*5*,*6* mutant which expresses *cda7* might support this. These examples show that the CDAs in *U. maydis* have specific as well as redundant functions either alone or in certain combinations. To substantiate this in the future, one would need to construct strains which express only single *cda* genes under the control of the same promoter to make sure that comparable levels of protein are synthesized. The ideal chassis strain for this would be the strain in which six *cda* genes are inactivated and the seventh *cda* gene product can be depleted. Such genetic analyses should then ideally be complemented with a more detailed biochemical study of all active enzymes with respect to their specific substrates, pattern of deacetylation *in vitro* and during colonization, and how this affects host responses.

## MATERIALS AND METHODS

### Strains and growth conditions.

The Escherichia coli strains DH5α (Bethesda Research Laboratories), Rosetta 2 (DE3) (Merck KGaA), and BL21(DE3) (Merck KGaA) were used for cloning purposes. E. coli strains were grown in double yeast-tryptone (dYT) medium except for BL21(DE3), which was grown in Luria Bertani medium (LB). *U. maydis* strains were grown at 28°C in complete liquid medium (CM) containing either 1% glucose (45) or 1% arabinose as a carbon source, liquid yeast extract-peptone-sucrose light (YEPSL), potato dextrose (PD) agar, or on CM agar. Media and buffers are described in Text S1 in the supplemental material. Growth, filamentation, and stress assays were performed as described previously (46) (see Text S1).

10.1128/mBio.03419-20.10 TEXT S1 Supplemental materials and methods. Download Text S1, PDF file, 0.4 MB. Copyright © 2021 Rizzi et al. 2021 Rizzi et al. https://creativecommons.org/licenses/by/4.0/ This content is distributed under the terms of the Creative Commons Attribution 4.0 International license.

To deplete *cda2*, SG200 *cda1,3,4,5,6*^em^Δ*7*P_crg_:*cda2* was grown in CM containing arabinose, and cells were harvested by centrifugation, washed with H_2_O, and suspended in CM with arabinose or glucose to a final optical density at 600 nm (OD_600_) of 0.2. Growth was followed by microscopy. The same procedure was applied to assay complementation of viability by introducing *cda6* from *U. maydis* (SG200 *cda1,3,4,5,6*^em^Δ*7*P_crg_:*cda2*,P_cda6_:*cda6*) and *cda6* from *S. scitamineum* placed under the control of the *U. maydis cda2* promoter (SG200 *cda1,3,4,5,6*^em^Δ*7*P_crg_:*cda2*,P_cda2_:*Sscda6*). To test viability, the same strains were also streaked out on CM agar containing glucose or arabinose. Single colonies from the CM agar-glucose plates were restreaked for single colonies on the same medium to observe the colony phenotype.

### Plasmid and strain construction.

PCRs were performed using the Phusion high-fidelity DNA polymerase (New England Biolabs). Templates were either SG200 genomic DNA, indicated plasmid DNAs, or double-stranded DNA fragments. Restriction enzymes were all supplied by New England Biolabs. Ligations were performed using a Gibson assembly kit (New England Biolabs). *U. maydis* underwent protoplast-mediated transformation (47). To generate *U. maydis* mutants and in-locus promoter replacements, the established CRISPR-Cas9 multiplex system was used (35). Gene replacement using a PCR-based approach (36) was used for *cda7*. Gene and promoter replacements and integrations into the *ip* locus (48) were verified by Southern blotting, and Cas9-induced point mutations were verified by sequencing. All *U. maydis* strains used in this study are listed in Table S1A. Plasmids and how they were generated is described in Table S1B. Oligonucleotides used for cloning are listed in Table S1C. All target sequences for the guide RNA constructs were designed using the E-CRISP tool (www.e-crisp.org) (49); double-stranded DNAs encoding the single guide RNAs (sgRNAs) for CRISPR-Cas9 are listed in Table S1D.

10.1128/mBio.03419-20.9 TABLE S1 Strains, plasmids, oligonucleotides, double-stranded DNA fragments, and accession numbers. Download Table S1, PDF file, 0.4 MB. Copyright © 2021 Rizzi et al. 2021 Rizzi et al. https://creativecommons.org/licenses/by/4.0/ This content is distributed under the terms of the Creative Commons Attribution 4.0 International license.

### CAP-sfGFP, CAP-mKATE2, sfGFP, and mKATE2 purification.

To obtain probes for chitosan detection, CAP-sfGFP and CAP-mKATE2 as well as the controls sfGFP and mKATE2 were heterologously expressed in E. coli Rosetta 2 (DE) according to reference 31, except for breaking cells by French press and protein quantification, according to M. M. Bradford (50).

### Heterologous expression of CDA proteins.

E. coli BL21(DE3) cells heterologous expressing E. coli dicodon-optimized *U. maydis* chitin deacetylases (http://dicodon-optimization.appspot.com/) were grown in 500 ml LB supplemented for autoinduction with media M, medium 5052, and 100 μg ml^−1^ ampicillin at 26°C and 120 rpm for 48 h (51). Cells were harvested, resuspended in 15 ml fast protein liquid chromatography (FPLC) washing buffer, and stored at −20°C. Cells were thawed on ice and incubated with 100 U of Benzonase nuclease (Merck KGaA) resuspended in 2 M MgCl_2_ and 2 ml of high-salt buffer for 10 min. Cells were lysed by sonication using Branson Digital Sonifier model 250-D (Branson). Sonicated cells were centrifuged (40 min, 40,000 × *g*, 4°C), and proteins in the supernatant were visualized by SDS-PAGE and Western blotting using horseradish peroxidase (HRP)-conjugated Strep-Tactin and the chemiluminescent substrate luminol (IBA). Recombinant enzymes were recovered from the supernatant using Strep-Tactin affinity chromatography (Strep-Tactin XT; IBA) using the ÄKTA pure system (GE Healthcare Europe GmbH) and eluted from the column using FLPC washing buffer containing 50 mM biotin. Affinity-purified proteins were rebuffered in 20 mM triethanolamine (TEA; pH 8.0) and stored at 4°C. Proteins were quantified according to M. M. Bradford (50).

SG200 strains expressing *cda3* or *cda7* from the constitutive actin promoter (SG200 P_act_*cda3*-StrepTag, SG200 P_act_*cda7*-StrepTag) were grown overnight in YEPSL and used as inoculum for 1 liter CM containing glucose adjusted to an OD_600_ of 0.2. At an OD_600_ of 1.4 at 28°C, cells were pelleted, and the supernatants were filtered with Millipore Express membrane, pore size 0.22 μm (Merck KGaA). The supernatants were concentrated using Amicon columns (Merck KGaA, cutoff of 30 kDa). Proteins were purified using Strep-Tactin affinity chromatography (Strep-Tactin Sepharose; IBA), rebuffered in 20 mM TEA (pH 8.0), and quantified according to M. M. Bradford (50).

### CDA activity assay and LC-MS analysis.

Enzyme activity assays were carried out by incubating 1 μM purified protein with 250 μM pentaacetyl-chitopentaose substrate (A5) (Megazyme) in 50 mM TEA (pH 7.0) at 37°C for 20 h. The enzymatic reaction was stopped using equal parts of 1% formic acid. Colletotrichum lindemuthianum CDA (*Cl*CDA) recombinantly produced in E. coli was used as a positive control (32). The resulting products were analyzed through UHPLC-ESI-MS (52) with some modifications (Text S1). Data were processed using Data Analysis v4.1 software (Bruker Daltonics GmbH). Chitosan oligomers were quantified via their peak areas.

### Plant infections.

Strains were grown in YEPSL medium to an OD_600_ of 0.8 to 1.2. Cells were harvested via centrifugation and resuspended in H_2_O to a final OD_600_ of 1.0. The suspension was syringe inoculated into 7-day-old *Zea mays* seedlings variety Early Golden Bantam (Urban Farmer). At least three independent infection experiments were carried out, and disease symptoms were evaluated according to established disease rating criteria (30). To statistically assess strain differences in virulence, virulence scores shown in color in the figures were converted to numbers according to reference 53, from 0 (indicating no symptoms) to 8 (indicating dead plants). Differences between virulence scores were assessed by a Kruskal-Wallis rank sum test followed by a Games-Howell *post hoc* test.

### Sample preparation and microscopic analyses.

To detect chitin and chitosan in budding cells, strains were grown in YEPSL to an OD_600_ of 1.00. Cells were centrifuged and resuspended in FLPC washing buffer containing 3% bovine serum albumin (BSA). After 30 min, cells were centrifuged and resuspended in the same solution containing WGA (AF488 or AF594) at 10 μg ml^−1^ and CAP (sfGFP/mKATE2) at 50 μg ml^−1^. As a control, cells were stained with WGA (AF488 or AF594) at 10 μg ml^−1^ and sfGFP or mKATE2 at 50 μg ml^−1^. Samples were incubated for 1 h at room temperature in the dark with shaking at 120 rpm, followed by three washes with FLPC buffer and observation by microscopy. For microscopy, cells were spotted onto a 2% agar pad on a microscopy slide.

To detect chitin and chitosan in hyphae of infected plant tissue, 2-cm segments from the third and fourth leaf were excised from a region 1 cm below the injection holes at 2 to 12 days postinfection (dpi) and incubated for 20 min in 10 μg ml^−1^ calcofluor white to stain hyphae on the leaf surface. Samples were washed three times with distilled water, cut in thin cross sections with a razor blade, and treated with digestion solution for 90 min at room temperature, followed by three washing steps with phosphate-buffered saline (PBS). Staining with WGA-AF488 and CAP-mKATE2 was according to the procedure applied for budding cells using staining with mKATE2 as control. Samples were analyzed by confocal microscopy.

Nuclei in budding cells of SG200, SG200 *cda1,3,4,5,6*^em^Δ*7*, and SG200 *cda1,3,4,5,6*^em^Δ*7*P_crg_:*cda2* after 24 h of growth in liquid CM containing arabinose or glucose were visualized by 4′,6-diamidino-2-phenylindole (DAPI) staining. Cells were fixed with 4% formaldehyde in PBS after 1 h of incubation and resuspended in 20 μg ml^−1^ DAPI in PBS containing 0.1% Triton X-100. After 1 h of incubation, cells were washed three times with PBS and observed by fluorescence microscopy.

### Microscopy.

Confocal microscopy was performed using a Leica TCS-SP8 confocal microscope (Leica Microsystems). mKATE2 was exited at 588 nm and detected at 618 to 653 nm. GFP was excited at 488 nm and detected at 498 to 524 nm. Calcofluor white was excited at 405 nm and detected at 429 to 499 nm. AF488 was excited at 488 nm and detected at 506 to 535 nm. AF594 was excited at 590 nm and detected at 602 to 640 nm. mCherry was excited at 561 nm and detected at 597 to 635 nm. Aniline blue was excited at 405 nm and detected at 630 to 655 nm. The Leica Application Suite Advanced Fluorescence software was used for image processing. If not indicated otherwise, images are horizontal projections of z-stacks.

Epifluorescence microscopy was performed with a Zeiss Axioplan 2 imaging microscope (Carl Zeiss AG) equipped with a CoolSNAP-HQ charge-coupled-device camera (Photometrics) and controlled by the imaging software MetaMorph (Universal Imaging). GFP was observed using GFP filters (ET470/40BP, ET495LP, and ET525/50BP) (Semrock). AF594 was observed using rhodamine filters (HC562/40BP, HC593LP, and HC624/40BP) (Semrock). DAPI staining was detected using the DAPI filter sets (HC375/11BP, HC409BS, and HC447/60BP) (Semrock). Image processing was performed with ImageJ (https://imagej.nih.gov/ij/).

### Data availability.

Genes and encoding protein sequences are available at NCBI or MaizeSequence.org (http://www.maizesequence.org) under the accession numbers described in Table S1E. Gene expression data were retrieved from RNA-seq data (29) (GEO database accession number GSE103876).

## References

[1] Akamatsu A, Wong HL, Fujiwara M, Okuda J, Nishide K, Uno K, Imai K, Umemura K, Kawasaki T, Kawano Y, Shimamoto K. 2013. An OsCEBiP/OsCERK1-OsRacGEF1-OsRac1 module is an essential early component of chitin-induced rice immunity. Cell Host Microbe 13:465–476. doi:10.1016/j.chom.2013.03.007. 23601108

[2] Hayafune M, Berisio R, Marchetti R, Silipo A, Kayama M, Desaki Y, Arima S, Squeglia F, Ruggiero A, Tokuyasu K, Molinaro A, Kaku H, Shibuya N. 2014. Chitin-induced activation of immune signaling by the rice receptor CEBiP relies on a unique sandwich-type dimerization. Proc Natl Acad Sci U S A 111:E404–E413. doi:10.1073/pnas.1312099111. 24395781 PMC3903257

[3] Liu T, Liu Z, Song C, Hu Y, Han Z, She J, Fan F, Wang J, Jin C, Chang J, Zhou JM, Chai J. 2012. Chitin-induced dimerization activates a plant immune receptor. Science 336:1160–1164. doi:10.1126/science.1218867. 22654057

[4] Gubaeva E, Gubaev A, Melcher RLJ, Cord-Landwehr S, Singh R, El Gueddari NE, Moerschbacher BM. 2018. 'Slipped sandwich' model for chitin and chitosan perception in arabidopsis. Mol Plant Microbe Interact 31:1145–1153. doi:10.1094/MPMI-04-18-0098-R. 29787346

[5] Fujikawa T, Kuga Y, Yano S, Yoshimi A, Tachiki T, Abe K, Nishimura M. 2009. Dynamics of cell wall components of *Magnaporthe grisea* during infectious structure development. Mol Microbiol 73:553–570. doi:10.1111/j.1365-2958.2009.06786.x. 19602150

[6] Fujikawa T, Sakaguchi A, Nishizawa Y, Kouzai Y, Minami E, Yano S, Koga H, Meshi T, Nishimura M. 2012. Surface alpha-1,3-glucan facilitates fungal stealth infection by interfering with innate immunity in plants. PLoS Pathog 8:e1002882. doi:10.1371/journal.ppat.1002882. 22927818 PMC3426526

[7] Stergiopoulos I, van den Burg HA, Okmen B, Beenen HG, van Liere S, Kema GH, de Wit PJ. 2010. Tomato Cf resistance proteins mediate recognition of cognate homologous effectors from fungi pathogenic on dicots and monocots. Proc Natl Acad Sci U S A 107:7610–7615. doi:10.1073/pnas.1002910107. 20368413 PMC2867746

[8] van den Burg HA, Harrison SJ, Joosten MHAJ, Vervoort J, de Wit PJGM. 2006. *Cladosporium fulvum* avr4 protects fungal cell walls against hydrolysis by plant chitinases accumulating during infection. Mol Plant Microbe Interact 19:1420–1430. doi:10.1094/MPMI-19-1420. 17153926

[9] van Esse HP, Bolton MD, Stergiopoulos I, de Wit PJGM, Thomma BPHJ. 2007. The chitin-binding *Cladosporium fulvum* effector protein Avr4 is a virulence factor. Mol Plant Microbe Interact 20:1092–1101. doi:10.1094/MPMI-20-9-1092. 17849712

[10] de Jonge R, van Esse HP, Kombrink A, Shinya T, Desaki Y, Bours R, van der Krol S, Shibuya N, Joosten MH, Thomma BP. 2010. Conserved fungal LysM effector Ecp6 prevents chitin-triggered immunity in plants. Science 329:953–955. doi:10.1126/science.1190859. 20724636

[11] Marshall R, Kombrink A, Motteram J, Loza-Reyes E, Lucas J, Hammond-Kosack KE, Thomma BP, Rudd JJ. 2011. Analysis of two in planta expressed LysM effector homologs from the fungus *Mycosphaerella graminicola* reveals novel functional properties and varying contributions to virulence on wheat. Plant Physiol 156:756–769. doi:10.1104/pp.111.176347. 21467214 PMC3177273

[12] Mentlak TA, Kombrink A, Shinya T, Ryder LS, Otomo I, Saitoh H, Terauchi R, Nishizawa Y, Shibuya N, Thomma BPHJ, Talbot NJ. 2012. Effector-mediated suppression of chitin-triggered immunity by *Magnaporthe oryzae* is necessary for rice blast disease. Plant Cell 24:322–335. doi:10.1105/tpc.111.092957. 22267486 PMC3289562

[13] Sanchez-Vallet A, Mesters JR, Thomma BP. 2015. The battle for chitin recognition in plant-microbe interactions. FEMS Microbiol Rev 39:171–183. doi:10.1093/femsre/fuu003. 25725011

[14] Gao F, Zhang BS, Zhao JH, Huang JF, Jia PS, Wang S, Zhang J, Zhou JM, Guo HS. 2019. Deacetylation of chitin oligomers increases virulence in soil-borne fungal pathogens. Nat Plants 5:1167–1176. doi:10.1038/s41477-019-0527-4. 31636399

[15] Kaku H, Shibuya N. 2016. Molecular mechanisms of chitin recognition and immune signaling by LysM-receptors. Physiol Mol Plant Pathol 95:60–65. doi:10.1016/j.pmpp.2016.02.003.

[16] Ride JP, Barber MS. 1990. Purification and characterization of multiple forms of endochitinase from wheat leaves. Plant Sci 71:185–197. doi:10.1016/0168-9452(90)90008-C.

[17] Cord-Landwehr S, Melcher RL, Kolkenbrock S, Moerschbacher BM. 2016. A chitin deacetylase from the endophytic fungus *Pestalotiopsis* sp. efficiently inactivates the elicitor activity of chitin oligomers in rice cells. Sci Rep 6:38018. doi:10.1038/srep38018. 27901067 PMC5128826

[18] Cord-Landwehr S, Richter C, Wattjes J, Sreekumar S, Singh R, Basa S, El Gueddari NE, Moerschbacher BM. 2020. Patterns matter part 2: chitosan oligomers with defined patterns of acetylation. React Funct Polym 151:104577. doi:10.1016/j.reactfunctpolym.2020.104577.

[19] Wattjes J, Sreekumar S, Richter C, Cord-Landwehr S, Singh R, El Gueddari NE, Moerschbacher BM. 2020. Patterns matter part 1: chitosan polymers with non-random patterns of acetylation. React Funct Polym 151:104583. doi:10.1016/j.reactfunctpolym.2020.104583.

[20] Grifoll-Romero L, Pascual S, Aragunde H, Biarnes X, Planas A. 2018. Chitin deacetylases: structures, specificities, and biotech applications. Polymers (Basel) 10:352. doi:10.3390/polym10040352. PMC6415152 30966387

[21] El Gueddari NE, Rauchhaus U, Moerschbacher BM, Deising HB. 2002. Developmentally regulated conversion of surface-exposed chitin to chitosan in cell walls of plant pathogenic fungi. New Phytol 156:103–112. doi:10.1046/j.1469-8137.2002.00487.x.

[22] Geoghegan IA, Gurr SJ. 2016. Chitosan mediates germling adhesion in *Magnaporthe oryzae* and is required for surface sensing and germling morphogenesis. PLoS Pathog 12:e1005703. doi:10.1371/journal.ppat.1005703. 27315248 PMC4912089

[23] Kuroki M, Okauchi K, Yoshida S, Ohno Y, Murata S, Nakajima Y, Nozaka A, Tanaka N, Nakajima M, Taguchi H, Saitoh K-i, Teraoka T, Narukawa M, Kamakura T. 2017. Chitin-deacetylase activity induces appressorium differentiation in the rice blast fungus *Magnaporthe oryzae*. Sci Rep 7:9697. doi:10.1038/s41598-017-10322-0. 28852173 PMC5575296

[24] Kappel L, Münsterkötter M, Sipos G, Escobar Rodriguez C, Gruber S. 2020. Chitin and chitosan remodeling defines vegetative development and *Trichoderma* biocontrol. PLoS Pathog 16:e1008320. doi:10.1371/journal.ppat.1008320. 32078661 PMC7053769

[25] Xu Q, Wang J, Zhao J, Xu J, Sun S, Zhang H, Wu J, Tang C, Kang Z, Wang X. 2020. A polysaccharide deacetylase from *Puccinia striiformis* f. sp. *tritici* is an important pathogenicity gene that suppresses plant immunity. Plant Biotechnol J 18:1830–1842. doi:10.1111/pbi.13345. 31981296 PMC7336287

[26] Baker LG, Specht CA, Lodge JK. 2011. Cell wall chitosan is necessary for virulence in the opportunistic pathogen *Cryptococcus neoformans*. Eukaryot Cell 10:1264–1268. doi:10.1128/EC.05138-11. 21784998 PMC3187048

[27] Hembach L, Bonin M, Gorzelanny C, Moerschbacher BM. 2020. Unique subsite specificity and potential natural function of a chitosan deacetylase from the human pathogen *Cryptococcus neoformans*. Proc Natl Acad Sci U S A 117:3551–3559. doi:10.1073/pnas.1915798117. 32015121 PMC7035615

[28] Fisher MC, Henk DA, Briggs CJ, Brownstein JS, Madoff LC, McCraw SL, Gurr SJ. 2012. Emerging fungal threats to animal, plant and ecosystem health. Nature 484:186–194. doi:10.1038/nature10947. 22498624 PMC3821985

[29] Lanver D, Muller AN, Happel P, Schweizer G, Haas FB, Franitza M, Pellegrin C, Reissmann S, Altmuller J, Rensing SA, Kahmann R. 2018. The biotrophic development of *Ustilago maydis* studied by RNA-Seq analysis. Plant Cell 30:300–323. doi:10.1105/tpc.17.00764. 29371439 PMC5868686

[30] Kamper J, Kahmann R, Bolker M, Ma LJ, Brefort T, Saville BJ, Banuett F, Kronstad JW, Gold SE, Muller O, Perlin MH, Wosten HA, de Vries R, Ruiz-Herrera J, Reynaga-Pena CG, Snetselaar K, McCann M, Perez-Martin J, Feldbrugge M, Basse CW, Steinberg G, Ibeas JI, Holloman W, Guzman P, Farman M, Stajich JE, Sentandreu R, Gonzalez-Prieto JM, Kennell JC, Molina L, Schirawski J, Mendoza-Mendoza A, Greilinger D, Munch K, Rossel N, Scherer M, Vranes M, Ladendorf O, Vincon V, Fuchs U, Sandrock B, Meng S, Ho EC, Cahill MJ, Boyce KJ, Klose J, Klosterman SJ, Deelstra HJ, Ortiz-Castellanos L, Li W, . 2006. Insights from the genome of the biotrophic fungal plant pathogen *Ustilago maydis*. Nature 444:97–101. doi:10.1038/nature05248. 17080091

[31] Nampally M, Moerschbacher BM, Kolkenbrock S. 2012. Fusion of a novel genetically engineered chitosan affinity protein and green fluorescent protein for specific detection of chitosan *in vitro* and *in situ*. Appl Environ Microbiol 78:3114–3119. doi:10.1128/AEM.07506-11. 22367086 PMC3346462

[32] Blair DE, Hekmat O, Schüttelkopf AW, Shrestha B, Tokuyasu K, Withers SG, van Aalten DMF. 2006. Structure and mechanism of chitin deacetylase from the fungal pathogen *Colletotrichum lindemuthianum*. Biochemistry 45:9416–9426. doi:10.1021/bi0606694. 16878976

[33] Hekmat O, Tokuyasu K, Withers SG. 2003. Subsite structure of the endo-type chitin deacetylase from a Deuteromycete, *Colletotrichum lindemuthianum*: an investigation using steady-state kinetic analysis and MS. Biochem J 374:369–380. doi:10.1042/BJ20030204. 12775215 PMC1223603

[34] Ramazzina I, Cendron L, Folli C, Berni R, Monteverdi D, Zanotti G, Percudani R. 2008. Logical identification of an allantoinase analog (puuE) recruited from polysaccharide deacetylases. J Biol Chem 283:23295–23304. doi:10.1074/jbc.M801195200. 18550550

[35] Schuster M, Schweizer G, Kahmann R. 2018. Comparative analyses of secreted proteins in plant pathogenic smut fungi and related basidiomycetes. Fungal Genet Biol 112:21–30. doi:10.1016/j.fgb.2016.12.003. 28089076

[36] Kamper J. 2004. A PCR-based system for highly efficient generation of gene replacement mutants in *Ustilago maydis*. Mol Genet Genomics 271:103–110. doi:10.1007/s00438-003-0962-8. 14673645

[37] Bottin A, Kamper J, Kahmann R. 1996. Isolation of a carbon source-regulated gene from *Ustilago maydis*. Mol Gen Genet 253:342–352. doi:10.1007/pl00008601. 9003321

[38] Matsuo Y, Tanaka K, Matsuda H, Kawamukai M. 2005. *cdal*^+^, encoding chitin deacetylase is required for proper spore formation in *Schizosaccharomyces pombe*. FEBS Lett 579:2737–2743. doi:10.1016/j.febslet.2005.04.008. 15862318

[39] Christodoulidou A, Bouriotis V, Thireos G. 1996. Two sporulation-specific chitin deacetylase-encoding genes are required for the ascospore wall rigidity of *Saccharomyces cerevisiae*. J Biol Chem 271:31420–31425. doi:10.1074/jbc.271.49.31420. 8940152

[40] Pammer M, Briza P, Ellinger A, Schuster T, Stucka R, Feldmann H, Breitenbach M. 1992. DIT101 (CSD2, CAL1), a cell cycle-regulated yeast gene required for synthesis of chitin in cell walls and chitosan in spore walls. Yeast 8:1089–1099. doi:10.1002/yea.320081211. 1293886

[41] Baker LG, Specht CA, Donlin MJ, Lodge JK. 2007. Chitosan, the deacetylated form of chitin, is necessary for cell wall integrity in *Cryptococcus neoformans*. Eukaryot Cell 6:855–867. doi:10.1128/EC.00399-06. 17400891 PMC1899242

[42] Mouyna I, Delliere S, Beauvais A, Gravelat F, Snarr B, Lehoux M, Zacharias C, Sun Y, de Jesus Carrion S, Pearlman E, Sheppard DC, Latge JP. 2020. What are the functions of chitin deacetylases in *Aspergillus fumigatus*? Front Cell Infect Microbiol 10:28. doi:10.3389/fcimb.2020.00028. 32117802 PMC7016196

[43] Doehlemann G, Wahl R, Horst RJ, Voll LM, Usadel B, Poree F, Stitt M, Pons-Kuhnemann J, Sonnewald U, Kahmann R, Kamper J. 2008. Reprogramming a maize plant: transcriptional and metabolic changes induced by the fungal biotroph *Ustilago maydis*. Plant J 56:181–195. doi:10.1111/j.1365-313X.2008.03590.x. 18564380

[44] Doering TL. 2009. How sweet it is! Cell wall biogenesis and polysaccharide capsule formation in *Cryptococcus neoformans*. Annu Rev Microbiol 63:223–247. doi:10.1146/annurev.micro.62.081307.162753. 19575556 PMC2880894

[45] Holliday R. 1974. *Ustilago maydis*, p 575–595. *In* King RC (ed), Bacteria, bacteriophages, and fungi: volume 1. Springer, Boston, MA. doi:10.1007/978-1-4899-1710-2_31.

[46] Krombach S, Reissmann S, Kreibich S, Bochen F, Kahmann R. 2018. Virulence function of the *Ustilago maydis* sterol carrier protein 2. New Phytol 220:553–566. doi:10.1111/nph.15268. 29897130

[47] Brachmann A, Konig J, Julius C, Feldbrugge M. 2004. A reverse genetic approach for generating gene replacement mutants in *Ustilago maydis*. Mol Genet Genomics 272:216–226. doi:10.1007/s00438-004-1047-z. 15316769

[48] Loubradou G, Brachmann A, Feldbrugge M, Kahmann R. 2001. A homologue of the transcriptional repressor Ssn6p antagonizes cAMP signalling in *Ustilago maydis*. Mol Microbiol 40:719–730. doi:10.1046/j.1365-2958.2001.02424.x. 11359577

[49] Heigwer F, Kerr G, Boutros M. 2014. E-CRISP: fast CRISPR target site identification. Nat Methods 11:122–123. doi:10.1038/nmeth.2812. 24481216

[50] Bradford MM. 1976. A rapid and sensitive method for the quantitation of microgram quantities of protein utilizing the principle of protein-dye binding. Anal Biochem 72:248–254. doi:10.1006/abio.1976.9999. 942051

[51] Studier FW. 2005. Protein production by auto-induction in high-density shaking cultures. Protein Expr Purif 41:207–234. doi:10.1016/j.pep.2005.01.016. 15915565

[52] Hamer SN, Cord-Landwehr S, Biarnés X, Planas A, Waegeman H, Moerschbacher BM, Kolkenbrock S. 2015. Enzymatic production of defined chitosan oligomers with a specific pattern of acetylation using a combination of chitin oligosaccharide deacetylases. Sci Rep 5:8716. doi:10.1038/srep08716. 25732514 PMC4346795

[53] Gold SE, Brogdon SM, Mayorga ME, Kronstad JW. 1997. The *Ustilago maydis* regulatory subunit of a cAMP-dependent protein kinase is required for gall formation in maize. Plant Cell 9:1585–1594. doi:10.1105/tpc.9.9.1585. 9338961 PMC157035

