# Supplementary material for: Chitosan and Chitin Deacetylase Activity Are Necessary for Development and Virulence of Ustilago maydis
Source: mBio. 2021 Mar 2;12(2):e03419-20. doi: 10.1128/mBio.03419-20 (PMC8092297; doi:10.1128/mBio.03419-20)
Supplement: FIG S1 [file mBio.03419-20-sf001.pdf]

A

Staining	non-dividing cells		dividing cells			
			division zone		pole mother cell	pole daughter cell
	1 pole	2 poles	cells with small bud	cells with big bud		
WGA-AF594	75% ± 9%	29% ± 13%	72% ± 3%	86% ± 14%	40% ± 7%	23% ± 8%
CAP-sfGFP	79% ± 7%	10%± 8%	73% ± 17%	93% ± 19%	39% ± 8%	19% ± 15%
Both stains	73% ± 12%	6% ± 5%	62% ± 10%	83% ± 17%	32% ± 7%	7% ± 5%

B

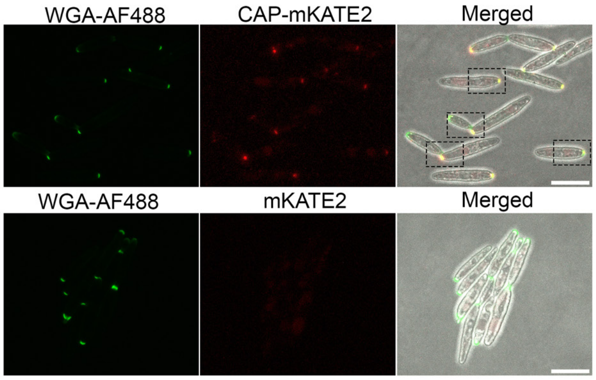

C

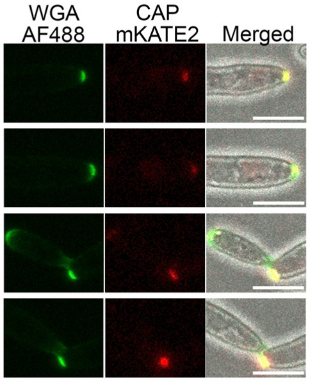

D

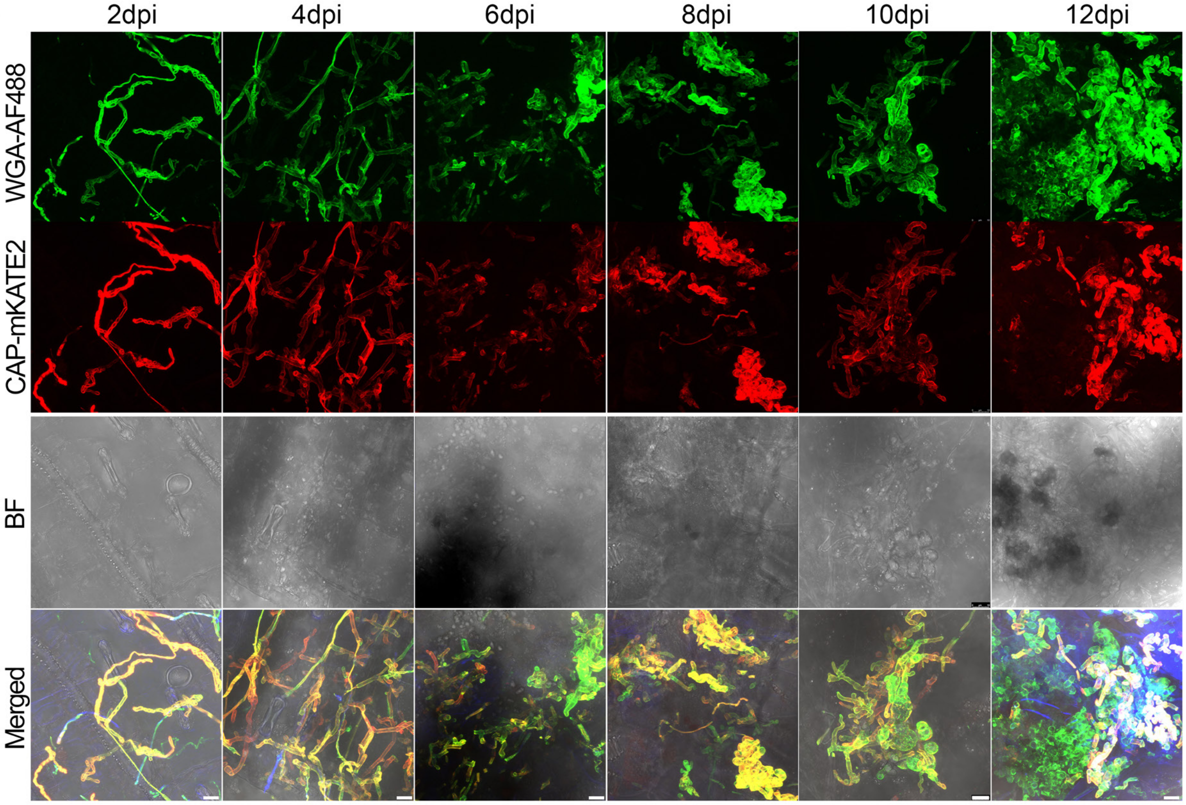
2dpi
4dpi
6dpi
8dpi
10dpi
12dpi
WGA-AF488
CAP-mKATE2
BF
Merged

E

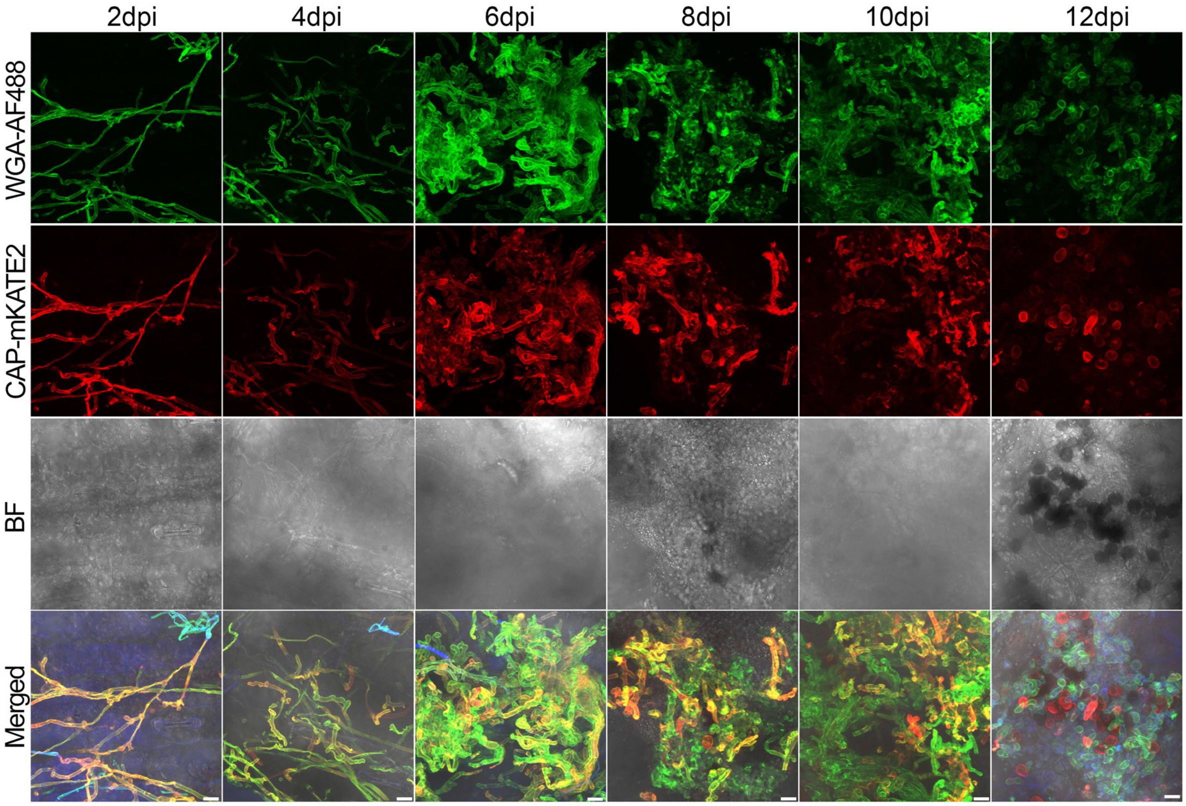
2dpi
4dpi
6dpi
8dpi
10dpi
12dpi
WGA-AF488
CAP-mKATE2
BF
Merged

FIG S1

Staining of chitin and chitosan in budding cells and biotrophic hyphae with WGA and CAP. (A) Quantification of presence of chitin and chitosan staining in budding cells of SG200. Mean values ± SD of 6 independent biological replicates. (B) Budding cells of SG200 were stained with WGA-AF488 (green) to detect chitin and CAP-mKATE2 (red) to detect chitosan (upper panel), or with mKATE2 as control (lower panel). Cells were observed by confocal microscopy. The images are projections of multiple Z-stacks. Scale bar: 10 µm. (C) Enlargements of the stippled boxes marked in A. Scale bar 10 µm. (D-E) Chitosan and chitin accessibility in the cell wall of *U. maydis* during plant infection. Leaf samples infected with the solopathogenic strain SG200 (D) or with FB1xFB2 (E) were collected at 2, 4, 6, 8, 10 and 12 dpi and stained with calcofluor (blue) for hyphae on the surface of the plant leaves. After digestion of the plant tissue, biotrophic hyphae were stained with WGA-AF488 for chitin (green) and with CAP-mKATE2 for chitosan (red). In addition, bright field (BF) and merging of the four channels are shown. The samples were observed by confocal microscopy and all images are projections of multiple Z-stacks. Scale bar: 10 µm.
