# Supplementary material for: Chitosan and Chitin Deacetylase Activity Are Necessary for Development and Virulence of Ustilago maydis
Source: mBio. 2021 Mar 2;12(2):e03419-20. doi: 10.1128/mBio.03419-20 (PMC8092297; doi:10.1128/mBio.03419-20)
Supplement: FIG S2 [file mBio.03419-20-sf002.pdf]

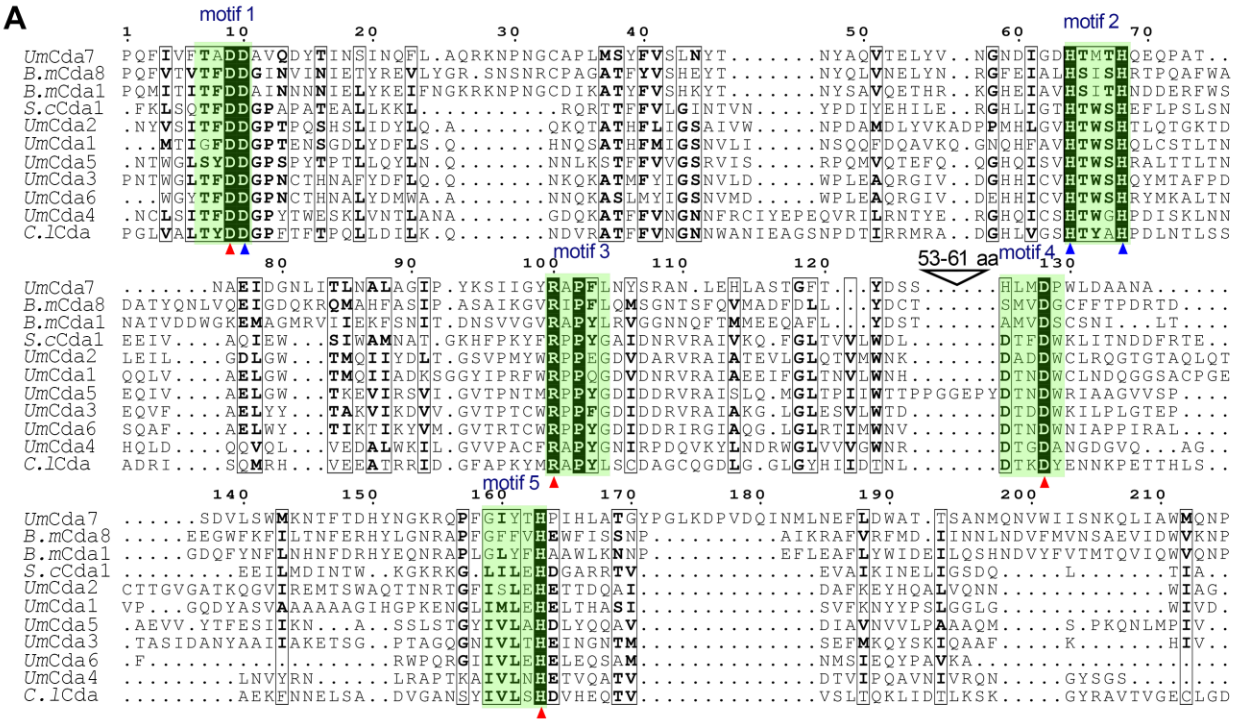

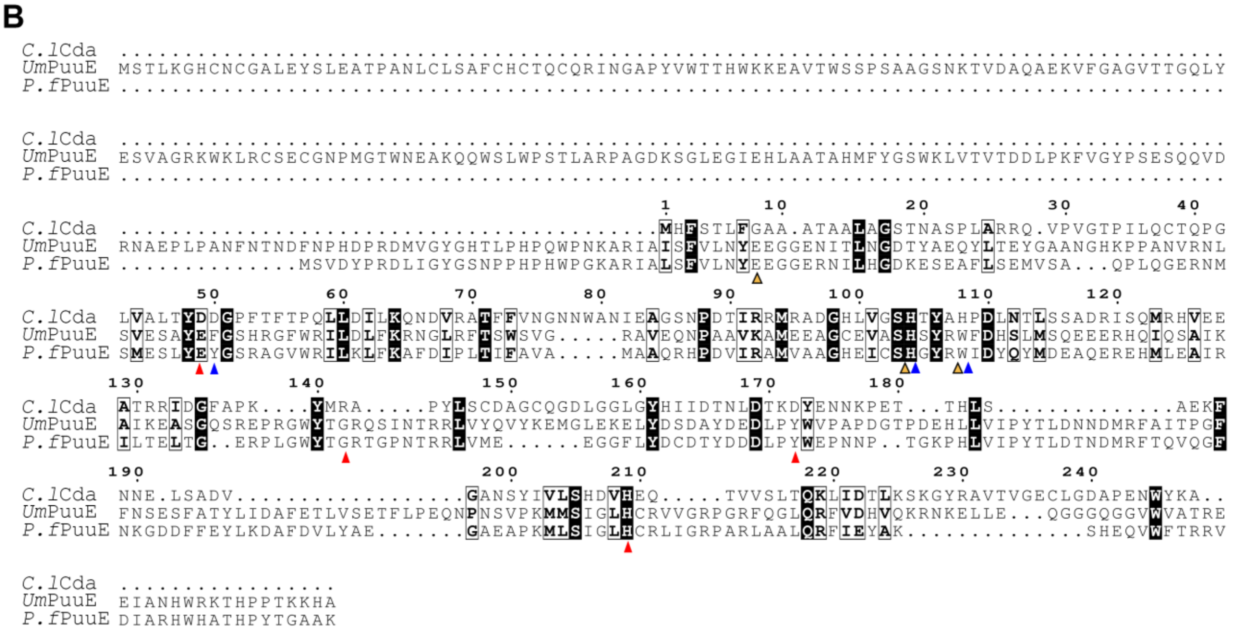

C

	Cda1	Cda2	Cda3	Cda4	Cda5	Cda6	Cda7
**Cda1**	-	36.60[a]	28.12	22.10	27.30	28.23	17.49
**Cda2**		-	29.68	23.21	26.74	27.38	20.97
**Cda3**			-	25.35	29.23	46.59	18.96
**Cda4**				-	25.82	24.64	15.87
**Cda5**					-	31.23	17.95
**Cda6**						-	17.41
**Cda7**							-

D

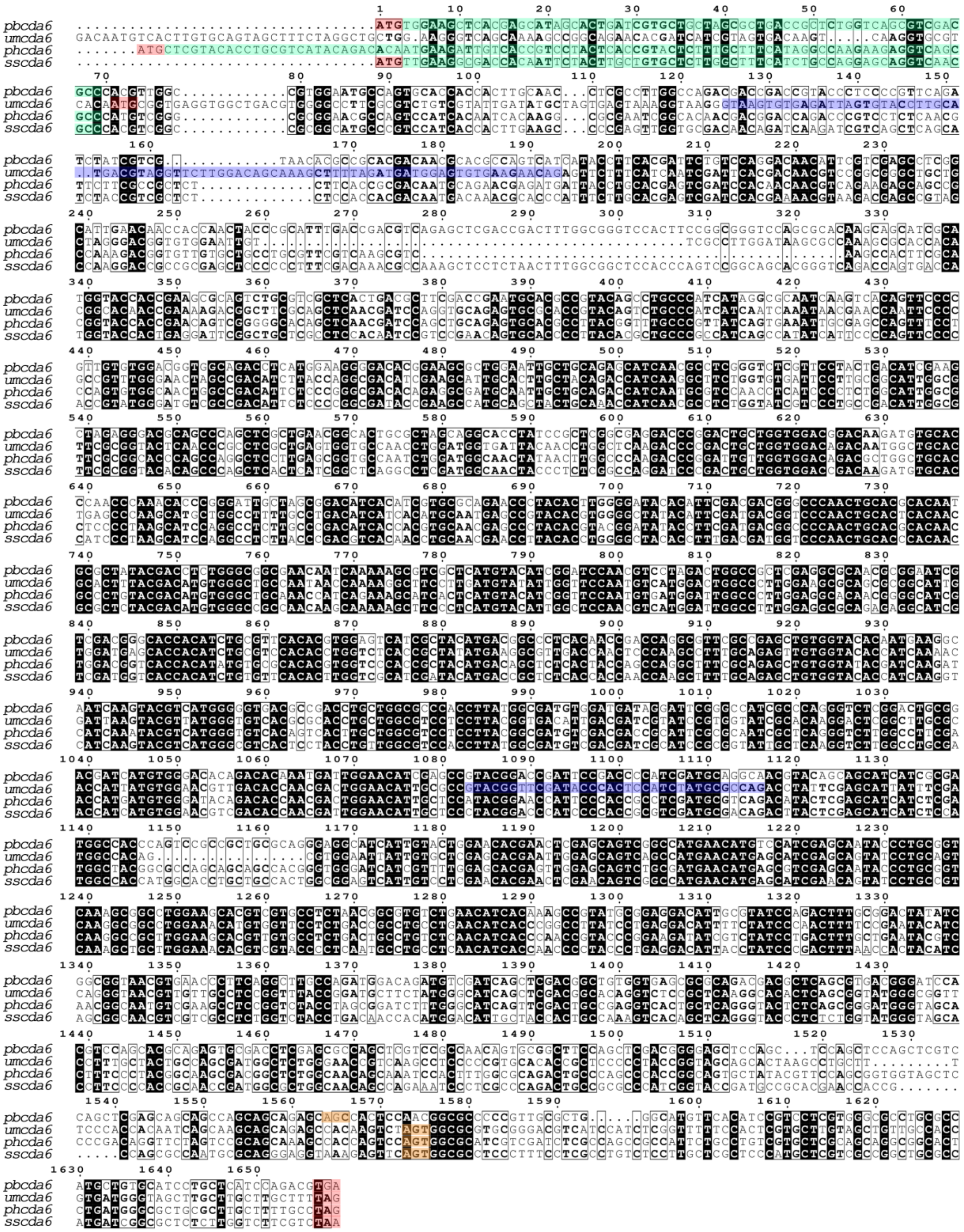

E

gac aat gtc act tgt gca gta gct ttc tag gct gct gga agg gtc agc aaa agc cgg cag
D N V T C A V A F - A A G R V S K S R Q
aac acg atc atc gta gtg aca agt caa ggt gcg tca caa tgc ggt gag gtg gct gac gtg
N T I I V V T S Q G A S Q C G E V A D V
ggg cct tcg cgt ctg tcg tat tga tat gct agt gag taa agg taa ggg taa gtg tga gat
G P S R L S Y - Y A S E - R - G - V - D
tag tgt acc ttg cat gac gta ggt tct tgg aca gca aag ctt tta gat gat gga gtg tga
- C T L H D V G S W T A K L L D D G V -
aga aca gag ttc ttt cat caa tcg att cac gac aac gtc cgg cgg gct gct gct agg gac
R T E F F H Q S I H D N V R R A A A R D
ggt gtg gaa ttg ttc gcc ttg gat aag cgc caa agc gca cca cac ggc aca acc gaa aag
G V E L F A L D K R Q S A P H G T T E K
acg gct tcg cag ctc aac gat cca ggt gca gag tgc gca ccg tac agt ctg ccc atc atc
T A S Q L N D P G A E C A P Y S L P I I
aat caa ata acg aac caa ttc ccc gcc gtt tgg gaa cta gcc gac atc tta cca ggc gac
N Q I T N Q F P A V W E L A D I L P G D
atc gaa gca ttg cac ttg cta cag acc atc aag gct tct ggt gtg att cct tgc ggc att
I E A L H L L Q T I K A S G V I P C G I
ggc gtt cgc ggt act caa ccc gcc tcg ctg agt ggt gcc aac ctg gat ggt gat tac aac
G V R G T Q P A S L S G A N L D G D Y N
ctg gct caa gac ccc gac tgc tgg tgg aca gac aat ggc tgc act gag ccc aag cat gct
L A Q D P D C W W T D N G C T E P K H A
ggc ctt ttg cct gac atc atc aca tgc aat gag ccg tac acg tgg ggc tat aca ttc gat
G L L P D I I T C N E P Y T W G Y T F D
gac ggt ccc aac tgc act cac aac gca ctt tac gac atg tgg gct gcc aat aac caa aag
D G P N C T H N A L Y D M W A A N N Q K
gct tcc ttg atg tat att ggt tcc aat gtc atg gac tgg ccc ttg gaa gcg cag cgc ggc
A S L M Y I G S N V M D W P L E A Q R G
att gtg gat gag cac cac atc tgc gtc cac acc tgg tct cac cgc tat atg aag gcg ttg
I V D E H H I C V H T W S H R Y M K A L
acc aac tcc caa gcc ttt gca gag ttg tgg tac acc atc aaa acg att aag tac gtt atg
T N S Q A F A E L W Y T I K T I K Y V M
ggt gtc acg cgc acc tgc tgg cgt cct cct tac ggt gac att gac gat cgt atc cgt ggt
G V T R T C W R P P Y G D I D D R I R G
atc gca caa gga ctc ggc ttg cgc acc att atg tgg aac gtt gac acc aac gac tgg aac
I A Q G L G L R T I M W N V D T N D W N
att gcg ccg tac ggt tcg ata ccc act cca tct atg cgc cag acc tat tcg agc att att
I A P Y G S I P T P S M R Q T Y S S I I
tcg atg gcc aca gcg tgg aat tat tgt gct cga gca cga att gga gca gtc agc cat gaa
S M A T A W N Y C A R A R I G A V S H E
cat gag cat cga gca gta tcc tgc agt caa ggc ggc ctg gaa aca tgt ggt tcc tct gac
H E H R A V S C S Q G G L E T C G S S D
cgc ctg cct gaa cat cac ccg gcc tta tcc tga gga cat ttt cta tcc caa ctt ttc cga
R L P E H H P A L S - G H F L S Q L F R
ata cat cca ggg taa cgt tgt tgc ctc cgg ttt acc gga tgc ttc tat ggg cat cag ctc
I H P G - R C C L R F T G C F Y G H Q L
gac ggc aca ggt ctc cgc tca agg cac act cag cgg tat ggg cgg ttc ctt tgc tac tgc
D G T G L R S R H T Q R Y G R F L C Y C
cag cga tgg ctc tgg gaa ccg tca agc ctc ccc cgt gca cac cgc tcc ccc tac cgg tag
Q R W L W E P S S L P R A H R S P Y R -
cag cac taa gcc tgc ttt tcc cac cac aat cag caa gca gca gag cca caa gtc tag tgg
Q H - A C F S H H N Q Q A A E P Q V - W
cgc gtg cgg gac gtc atc cat ctc ggt ttt tcc act cgt gct tgt agc tgt tgc cac cgt
R V R D V I H L G F S T R A C S C C H R
gat ggg tag ctt gct tgc ttg ctt tta
D G - L A C L L L

F

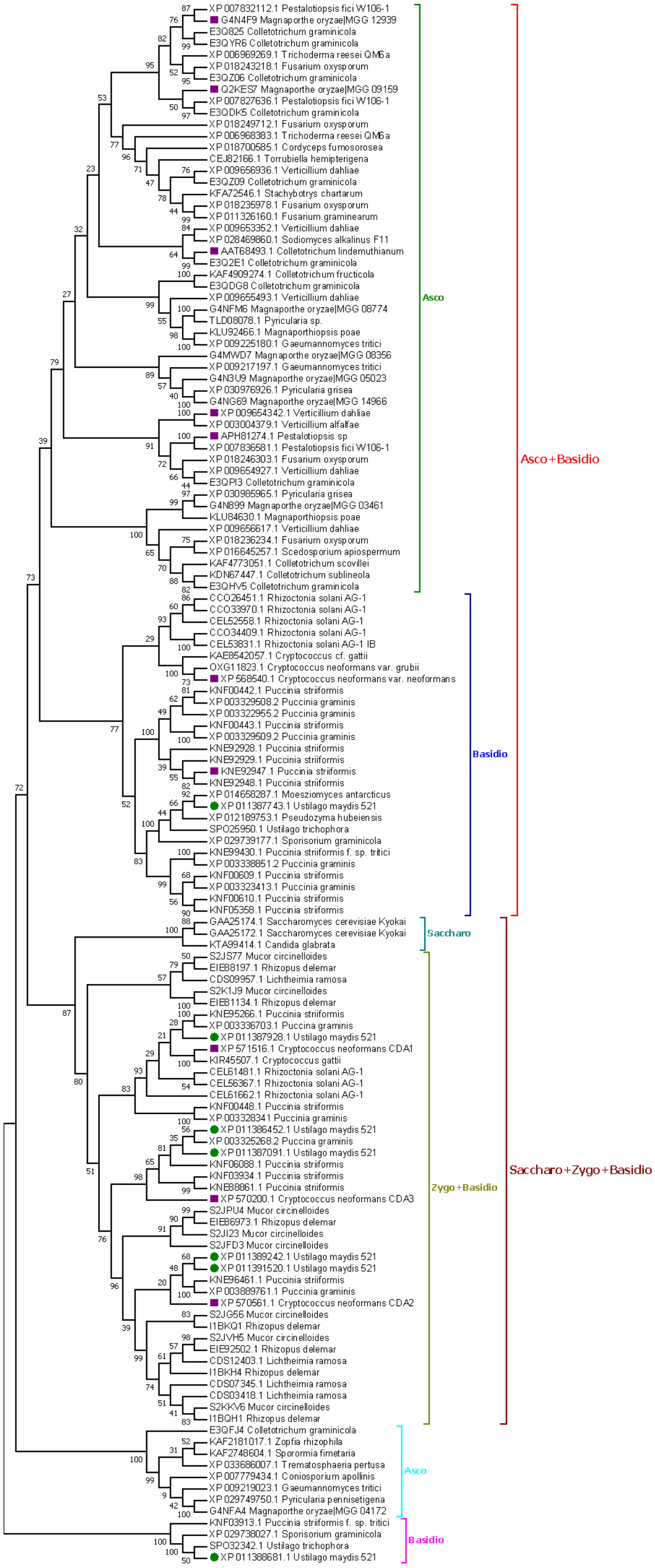

XP 007832112.1 Pestalotiopsis fici W106-1
G4N4F9 Magnaporthe oryzae|MGG 12939
E3Q825 Colletotrichum graminicola
E3QYR6 Colletotrichum graminicola
XP 006969269.1 Trichoderma reesei QM6a
XP 018243218.1 Fusarium oxysporum
E3QZ06 Colletotrichum graminicola
Q2KES7 Magnaporthe oryzae|MGG 09159
XP 007827636.1 Pestalotiopsis fici W106-1
E3QDK5 Colletotrichum graminicola
XP 018249712.1 Fusarium oxysporum
XP 006968383.1 Trichoderma reesei QM6a
XP 018700585.1 Cordyceps fumosorosea
CEJ82166.1 Torrubiella hemipterigena
XP 009656936.1 Verticillium dahliae
E3QZ09 Colletotrichum graminicola
KFA72546.1 Stachybotrys chartarum
XP 018235978.1 Fusarium oxysporum
XP 011326160.1 Fusarium graminearum
XP 009653352.1 Verticillium dahliae
XP 028469860.1 Sodiomyces alkalinus F11
AAT68493.1 Colletotrichum lindemuthianum
E3Q2E1 Colletotrichum graminicola
KAF4909274.1 Colletotrichum fructicola
E3QDG8 Colletotrichum graminicola
XP 009655493.1 Verticillium dahliae
G4NFM6 Magnaporthe oryzae|MGG 08774
TLD08078.1 Pyricularia sp.
KLU92466.1 Magnaporthiopsis poae
XP 009225180.1 Gaeumannomyces tritici
G4MWD7 Magnaporthe oryzae|MGG 08356
XP 009217197.1 Gaeumannomyces tritici
G4N3U9 Magnaporthe oryzae|MGG 05023
XP 030976926.1 Pyricularia grisea
G4NG69 Magnaporthe oryzae|MGG 14966
XP 009654342.1 Verticillium dahliae
XP 003004379.1 Verticillium alfalfae
APH81274.1 Pestalotiopsis sp
XP 007836581.1 Pestalotiopsis fici W106-1
XP 018246303.1 Fusarium oxysporum
XP 009654927.1 Verticillium dahliae
E3QPI3 Colletotrichum graminicola
XP 030985965.1 Pyricularia grisea
G4N899 Magnaporthe oryzae|MGG 03461
KLU84630.1 Magnaporthiopsis poae
XP 009656617.1 Verticillium dahliae
XP 018236234.1 Fusarium oxysporum
XP 016645257.1 Scedosporium apiospermum
KAF4773051.1 Colletotrichum scovillei
KDN67447.1 Colletotrichum sublineola
E3QHV5 Colletotrichum graminicola
CCO26451.1 Rhizoctonia solani AG-1
CCO33970.1 Rhizoctonia solani AG-1
CEL52558.1 Rhizoctonia solani AG-1
CCO34409.1 Rhizoctonia solani AG-1
CEL53831.1 Rhizoctonia solani AG-1 IB
KAE8542057.1 Cryptococcus cf. gattii
OXG11823.1 Cryptococcus neoformans var. grubii
XP 568540.1 Cryptococcus neoformans var. neoformans
KNF00442.1 Puccinia striiformis
XP 003329508.2 Puccinia graminis
XP 003322955.2 Puccinia graminis
KNF00443.1 Puccinia striiformis
XP 003329509.2 Puccinia graminis
KNE92928.1 Puccinia striiformis
KNE92929.1 Puccinia striiformis
KNE92947.1 Puccinia striiformis
KNE92948.1 Puccinia striiformis
XP 014658287.1 Moesziomyces antarcticus
XP 011387743.1 Ustilago maydis 521
XP 012189753.1 Pseudozyma hubeiensis
SPO25950.1 Ustilago trichophora
XP 029739177.1 Sporisorium graminicola
KNE99430.1 Puccinia striiformis f. sp. tritici
XP 003338851.2 Puccinia graminis
KNF00609.1 Puccinia striiformis
XP 003323413.1 Puccinia graminis
KNF00610.1 Puccinia striiformis
KNF05358.1 Puccinia striiformis
GAA25174.1 Saccharomyces cerevisiae Kyokai
GAA25172.1 Saccharomyces cerevisiae Kyokai
KTA99414.1 Candida glabrata
S2JS77 Mucor circinelloides
EIE88197.1 Rhizopus delemar
CDS09957.1 Lichtheimia ramosa
S2K1J9 Mucor circinelloides
EIE81134.1 Rhizopus delemar
KNE95266.1 Puccinia striiformis
XP 003336703.1 Puccina graminis
XP 011387928.1 Ustilago maydis 521
XP 571516.1 Cryptococcus neoformans CDA1
KIR45507.1 Cryptococcus gattii
CEL61481.1 Rhizoctonia solani AG-1
CEL56367.1 Rhizoctonia solani AG-1
CEL61662.1 Rhizoctonia solani AG-1
KNF00448.1 Puccinia striiformis
XP 003328341 Puccinia graminis
XP 011386452.1 Ustilago maydis 521
XP 003325268.2 Puccina graminis
XP 011387091.1 Ustilago maydis 521
KNF06088.1 Puccinia striiformis
KNF03934.1 Puccinia striiformis
KNE88861.1 Puccinia striiformis
XP 570200.1 Cryptococcus neoformans CDA3
S2JPU4 Mucor circinelloides
EIE86973.1 Rhizopus delemar
S2JI23 Mucor circinelloides
S2JFD3 Mucor circinelloides
XP 011389242.1 Ustilago maydis 521
XP 011391520.1 Ustilago maydis 521
KNE96461.1 Puccinia striiformis
XP 003889761.1 Puccinia graminis
XP 570561.1 Cryptococcus neoformans CDA2
S2JG56 Mucor circinelloides
I1BKQ1 Rhizopus delemar
S2JVH5 Mucor circinelloides
EIE92502.1 Rhizopus delemar
CDS12403.1 Lichtheimia ramosa
I1BKH4 Rhizopus delemar
CDS07345.1 Lichtheimia ramosa
CDS03418.1 Lichtheimia ramosa
S2KKV6 Mucor circinelloides
I1BQH1 Rhizopus delemar
E3QFJ4 Colletotrichum graminicola
KAF2181017.1 Zopfia rhizophila
KAF2748604.1 Sporormia fimetaria
XP 033686007.1 Trematosphaeria pertusa
XP 007779434.1 Coniosporium apollinis
XP 009219023.1 Gaeumannomyces tritici
XP 029749750.1 Pyricularia pennisetigena
G4NFA4 Magnaporthe oryzae|MGG 04172
KNF03913.1 Puccinia striiformis f. sp. tritici
XP 029738027.1 Sporisorium graminicola
SPO32342.1 Ustilago trichophora
XP 011388681.1 Ustilago maydis 521
Asco
Basidio
Asco+Basidio
Saccharo
Zygo+Basidio
Saccharo+Zygo+Basidio
Asco
Basidio

FIG S2.

Analysis of sequences of proteins containing a NodB domain from *U. maydis*. (A) Alignment of the NodB domains (defined by InterPro) of CDAs from *U. maydis* (*Um*Cda1, *Um*Cda2, *Um*Cda3, *Um*Cda4, *Um*Cda5, *Um*Cda6, and *Um*Cda7), and of the CDA protein from *C. lindemuthianum* (*C.l*Cda), Cda1 from *S. cerevisiae* (*Sc*Cda1) and *B. mori* Cda1 and Cda8 (*B.m*Cda1 and *B.m*Cda8), using Clustal Omega 12.1 (Madeira F, Park YM, Lee J, Buso N, Gur T, Madhusoodanan N, Basutkar P, Tivey ARN, Potter SC, Finn RD, Lopez R. Nucleic Acids Res 47:W636-W641, 2019). Accession numbers of the respective genes are listed in **Table S1E**. The conserved motifs for CDA catalytic activity are highlighted in green, and conserved active site residues within these motifs are marked with red arrows. Conserved residues for zinc binding are marked with blue arrows. *Um*Cda7, *B.m*Cda1, and *B.m*Cda8 contain an insertion between motif 3 and motif 4 and this was removed to improve the alignment. The insert in *Um*Cda7 is STASVPVTDPNTDAFWPYTLDNGMANDCNSVANICGGQPKLPGFWEIPMYAIFDERGA AGA. In *B.m*Cda1, the insert is ITAPLSNPRLCPYTMYFRMPHRCHGNLQSCPTRSHAVWEMVMNELDRREDPSNDEYLP GC and in *B.m*Cda8, the insert is DCTWPTTALTNPGLWPYTLHHESIQDCIIPPCPTASIPGPWVLPMISWRDLNNFPC. (B) Alignment of the amino acid sequences of the full length of putative PuuE protein of *U. maydis* (*Um*PuuE), CDA protein from *C. lindemuthianum*, and PuuE protein from *Pseudomonas fluorescence* (*P.f*PuuE) using Clustal Omega 12.1. The conserved residues for CDA activity in *C.l*Cda, marked as in A. Characteristic modification of the zinc-binding triad of CDA DHH to EHW in PuuE proteins are marked with yellow arrows. (C) Percentage of identity between Cdas from *U. maydis*. Determined with Clustal Omega12.1. (D) Alignment of the nucleotide sequence of cda6 from *U. maydis* (*umcda6*), and the ORF of the orthologous genes (identified by Ortho DB v10 and by manual checking of synteny) from, *S. Sporisorium scitamineum* (*SSCI30930.1*, *sscda6*), *Pseudozyma hubeiensis* (*PHSY_002331*, *phcda6*), and *Pseudozyma brasiliensis* (*PSEUBRA_SCAF18g04655*, *pbcda6*) performed with Clustal Omega 12.1. The predicted ATGs and stop codons are highlighted in red, the sequence of the predicted signal peptides are marked in green, and the nucleotides that encode for the putative omega site of the GPI anchor are marked in orange. The regions annotated as introns in umcda6 are highlighted in blue. (E) Nucleotide sequence and translated amino acid sequence of the central part of the ORF of *cda6* from *U. maydis* by ExPASy Translate (http://web.expasy.org/translate/) containing four of the five conserved domains and extended manually toward the 5´and 3´end of the respective gene without assuming

introns. As an in frame ATG would be located 3´to the first motif, this would reduce the size of Cda6 to 178 amino acids, eliminate the first conserved motif critical for CDA activity, and eliminate the putative GPI anchor. Conserved motifs for CDA activity are highlighted in green, regions annotated as introns are highlighted in blue, an in frame ATG is marked in magenta, and stop codons are given in red. (F) Phylogenetic tree of fungal CDAs. Amino acid sequences of putative or known fungal chitin deacetylases were retrieved from the NCBI, Ensembl, Uniprot, and JGI genome database portals and aligned with the online program MAFFT version 7 (Katoh K, Rozewicki J, Yamada KD. Brief Bioinform 20:1160-1166, 2019). The evolutionary analysis was carried out in MEGA7.0 (Kumar S, Stecher G, Tamura K. Mol Biol Evol 33:1870-4, 2016). The phylogenetic tree was constructed using the neighbor joining method and by implying Poisson correction substitution model with uniform rates among the sites. In the tree, each enzyme is labeled with its unique id followed by the name of the organism; enzymes highlighted with a green circle are from the *U. maydis* strain 521, and proteins highlighted with a purple square are CDAs that were either biochemically characterized or studied by knock-out mutants, as mentioned in the text.
