# Supplementary material for: Chitosan and Chitin Deacetylase Activity Are Necessary for Development and Virulence of Ustilago maydis
Source: mBio. 2021 Mar 2;12(2):e03419-20. doi: 10.1128/mBio.03419-20 (PMC8092297; doi:10.1128/mBio.03419-20)
Supplement: FIG S3 [file mBio.03419-20-sf003.pdf]

A

Gene	axenic	12 hpi	24 hpi	2 dpi	4 dpi	6 dpi	8 dpi	12 dpi
cda1	961 ± 7472[a,b]	306 ± 71	269 ± 20	205 ± 29	39 ± 16	34 ± 9	13 ± 2	167 ± 49
cda2	1169 ± 248	630 ± 159	902 ± 223	2053 ± 68	432 ± 75	358 ± 24	213 ± 35	283 ± 62
cda3	31 ± 34	990 ± 151	828 ± 51	867 ± 49	548 ± 51	403 ± 27	442 ± 25	554 ± 149
cda4	138 ± 98	214 ± 90	913 ± 318	3928 ± 181	1247 ± 308	477 ± 112	201 ± 112	178 ± 64
cda5	174 ± 41	173 ± 92	287 ± 111	238 ± 26	139 ± 29	132 ± 14	129 ± 36	200 ± 16
cda6	0	4 ± 5	7 ± 4	0	2 ± 2	1 ± 0	2 ± 1	1 ± 0
cda7	265 ± 46	405 ± 23	593 ± 50	870 ± 87	735 ± 29	757 ± 94	868 ± 37	869 ± 30

B

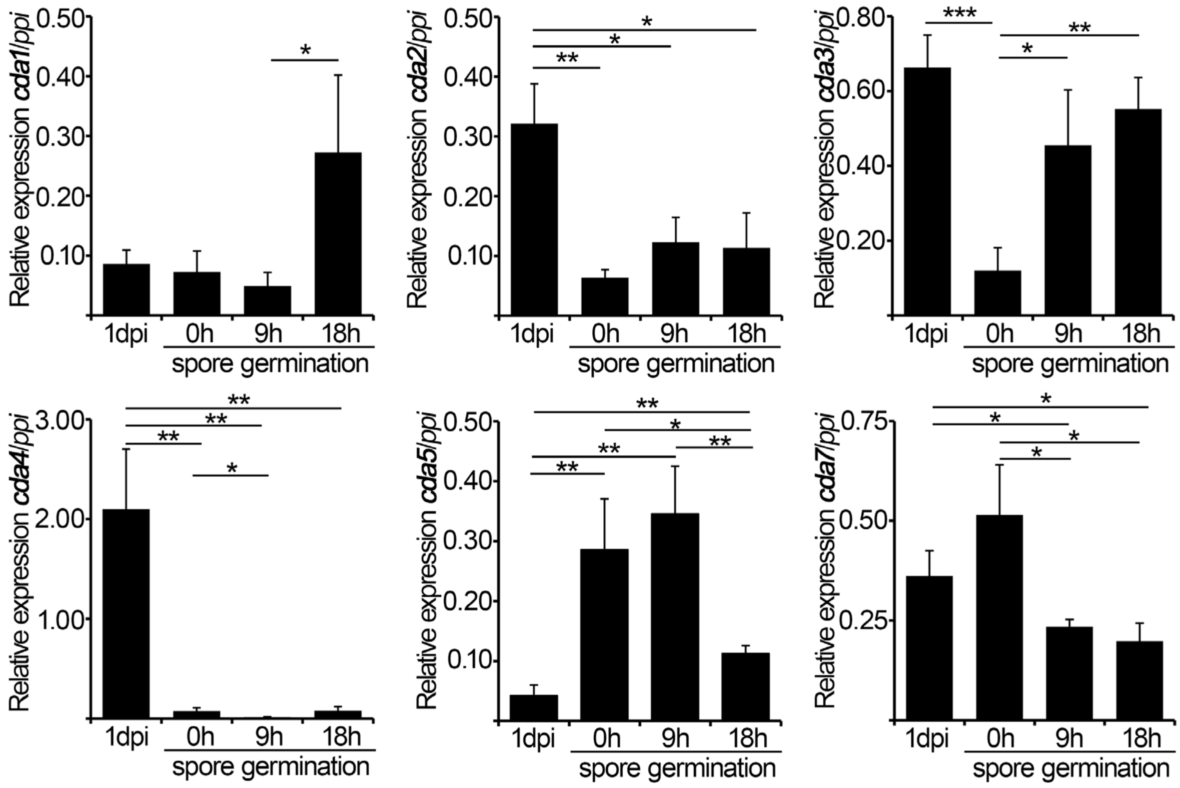

FIG S3.

Expesssion of *cda* genes during development of *U maydis*. (A) DESeq2-normalized read counts of *cda* genes at different time points of *Z. mays* infection with *U. maydis* FB1xFB2. Original data from **Fig 2B**. Data is obtained from (Lanver D, Muller AN, Happel P, Schweizer G, Haas FB, Franitza M, Pellegrin C, Reissmann S, Altmuller J, Rensing SA, Kahmann R. Plant Cell 30:300-323, 2018). Mean_perkb ± SD_perkb average of three independent replicates. (B) Expression of

cda genes from *U. maydis* in spores and during spore germination. qRT-PCR was used to determine which *cda* genes are expressed in spores and during spore germination because these stages were not represented in Lanver et al. (2018) data set. RT-qPCR was performed with RNA extracted from leaves after 1 dpi with FB1xFB2 as reference and from spores collected from cobs infected with FB1xFB2 as well as from spores after 9 and 18 h of germination. Expression of the *cda* genes was determined relative to the constitutively expressed *ppi* gene. Averages of three biological replicates are shown. Error bars indicate ± SD. Significant differences determined by two-side unpaired Student´s t-test (*, $P \leq 0.05$; **, $P \leq 0.01$; ***, $P \leq 0.001$).
