# Supplementary material for: Chitosan and Chitin Deacetylase Activity Are Necessary for Development and Virulence of Ustilago maydis
Source: mBio. 2021 Mar 2;12(2):e03419-20. doi: 10.1128/mBio.03419-20 (PMC8092297; doi:10.1128/mBio.03419-20)
Supplement: FIG S4 [file mBio.03419-20-sf004.pdf]

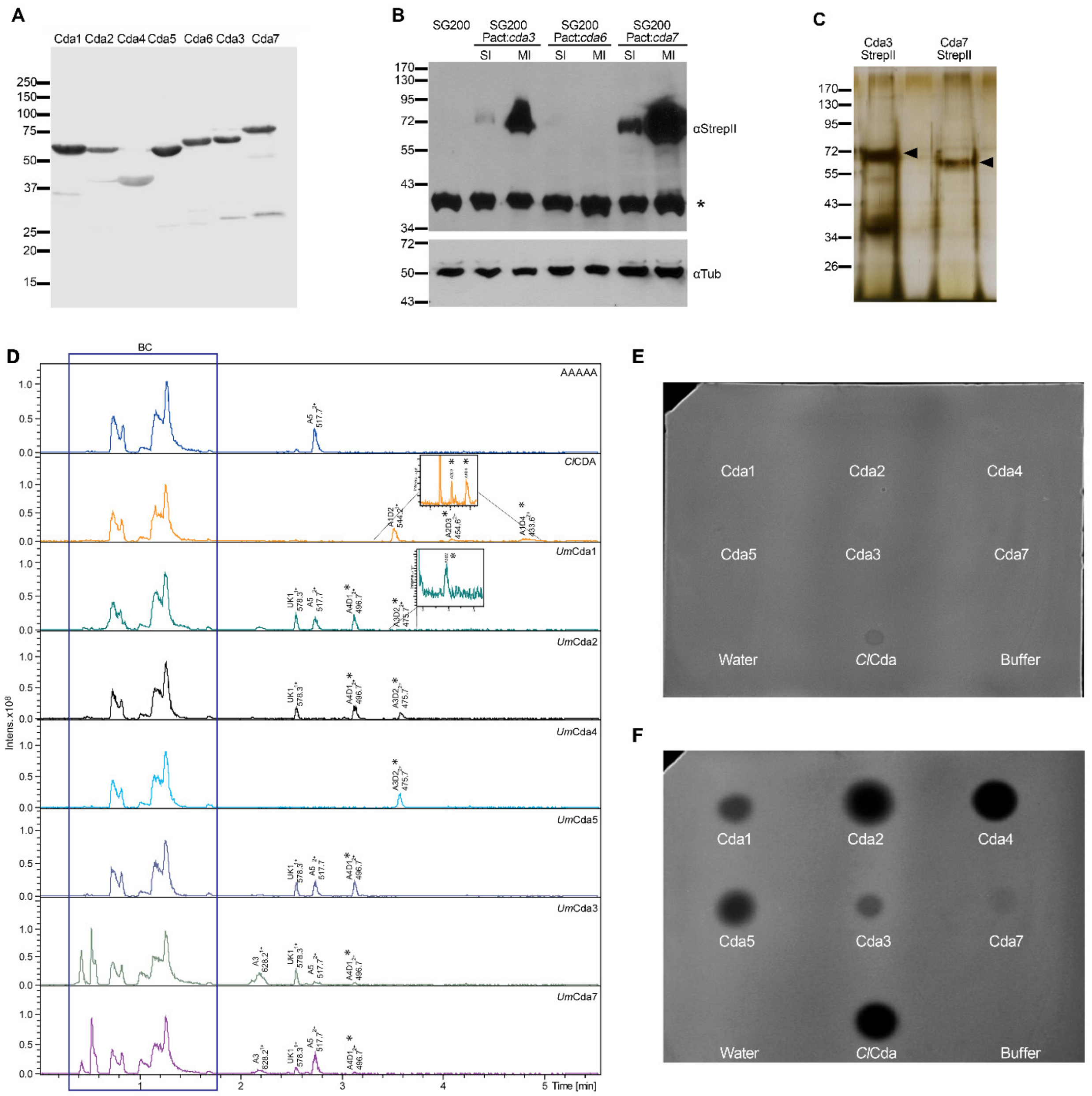

FIG S4.

Purification and activity of *U maydis* CDA proteins. (A) Western blot analysis of purified codon optimised StrepII tagged *U. maydis* CDAs after heterologous expression in *E. coli* BL21 (DE3). StrepII tagged proteins were visualized after immunoblotting using horseradish peroxide (HRP)-conjugated Strep-Tactin and the chemioluminescent substrate luminol. Expected molecular weights for StrepII tagged CDAs is 56 kDa for Cda1, 60 kDa for Cda2, 60 kDa for Cda3, 46 kDa for Cda4, 59 kDa for Cda5, 63 kDa for Cda6 and 69 kDa for Cda7. (B) Cda3, Cda6, and Cda7 were expressed constitutively in SG200 from the Pactin promoter after replacing the GPI anchor

sequence with a StrepII-tag. Cells containing single (SI) or multiple (MI) insertions of *P_{act}cda3-strep*, *P_{act}cda6-strep* or *P_{act}cda7-strep* were grown in YEPSL. Total cell lysates were analyzed by western blot with anti-StrepII (αStrepII) antibody (upper panel) and with anti-tubulin (αTub) antibody as a gel loading control (lower panel). Expected molecular weights of StrepII tagged proteins with signal peptide attached are 51 kDa for Cda3, 49 kDa for Cda6 and 57 kDa for Cda7. Unspecific bands are marked with an asterisk. (C) Silver staining ofpurified Cda3-StrepII (expected molecular weight 49 kDa) and Cda7-StrepII (expected molecular weight 55 kDa) after enrichment from the supernatants of strains analyzed in (B). Proteins representing the purified secreted proteins are labeled with arrowtips. (D) Reaction products of *U. maydis* CDAs. UHPLC-ESI-MS1 base peak chromatograms of the products of *U. maydis* CDAs (Cda1, Cda2, Cda3, Cda4, Cda5, and Cda7) and *C. lindemuthianum* CDA (*Cl*CDA) on the pentaacetyl-chitopentaose substrate, A5 (AAAAA). BC marks peaks from buffer components carried along during the purification. A5-derived reaction products are marked with asterisks. UK1, A1D2, and A3 with monoisotopic values (m/z) of 578.3, 544.2 and 628.2 respectively, likely correspond to degradation products. Inserts represent enlargements of relevant peak areas. In the *Um*CDA4 products, trace amounts of A4D1 and A2D3 (ca. 5 and 7%, respectively) were also detected. (E-F) Dot activity gel of purified *U. maydis* CDA proteins. The in-gel assay was performed by incorporating glycol-chitin into an acrylamide gel and adding 5 µl-drops of recombinant *Um*CDAs or *Cl*Cda as a positive control (Cda1, Cda2, Cda4, Cda5, and *Cl*Cda: 500 ng, Cda3: 615 ng, Cda7: 545 ng) in buffer (50 mM TEA, pH 7), followed by incubation overnight at 37°C, then washing the gel with water. The absence of dark spots upon chitin staining using calcofluor indicated the absence of chitinase activity (E), while the occurrence of dark spots after HNO_2 depolymerisation of chitosan indicated the presence of CDA activity (F).
