# Supplementary material for: Chitosan and Chitin Deacetylase Activity Are Necessary for Development and Virulence of Ustilago maydis
Source: mBio. 2021 Mar 2;12(2):e03419-20. doi: 10.1128/mBio.03419-20 (PMC8092297; doi:10.1128/mBio.03419-20)
Supplement: FIG S5 [file mBio.03419-20-sf005.pdf]

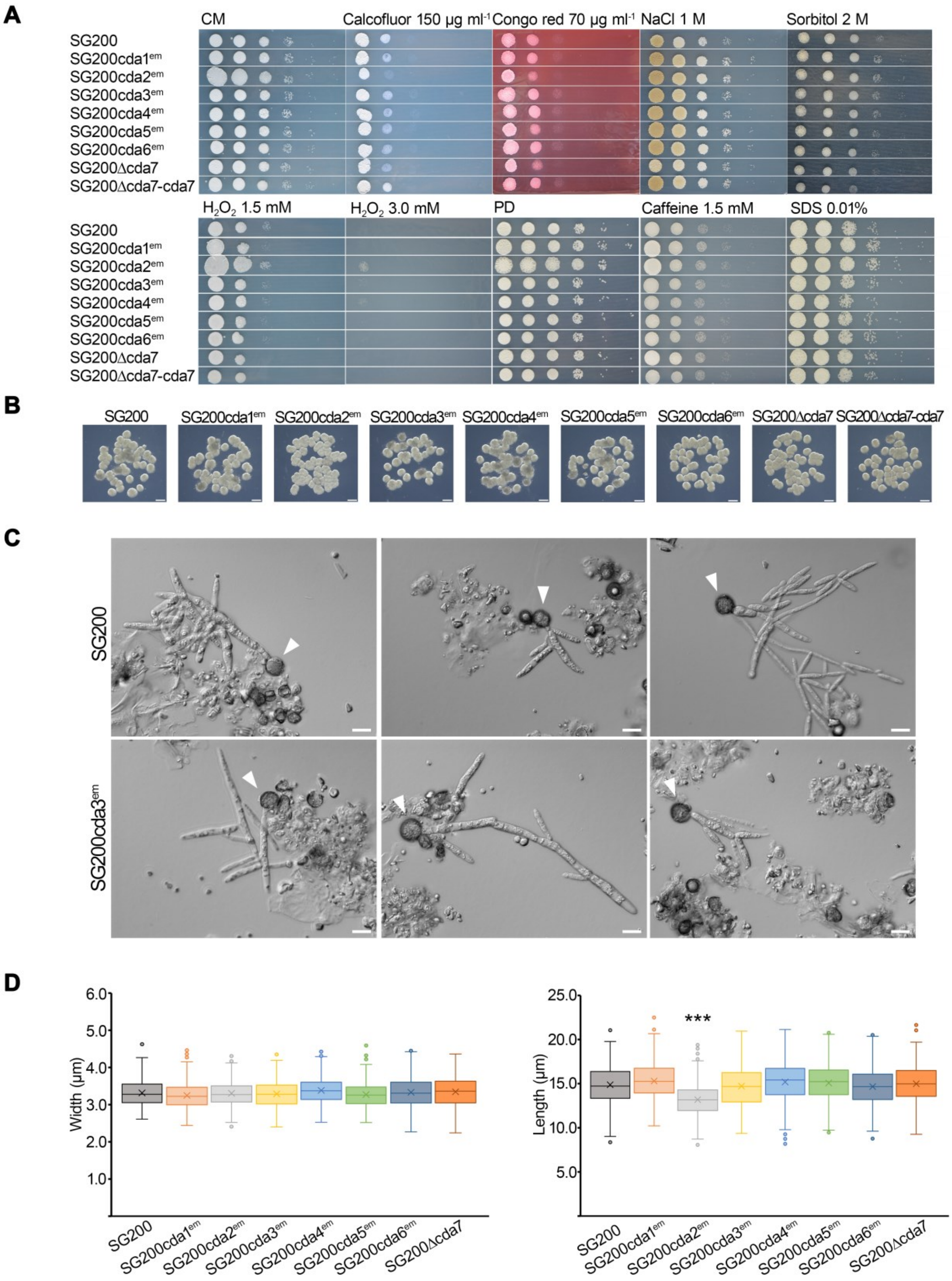

A
CM
Calcofluor 150 μg ml-1
Congo red 70 μg ml-1
NaCl 1 M
Sorbitol 2 M
SG200
SG200cda1em
SG200cda2em
SG200cda3em
SG200cda4em
SG200cda5em
SG200cda6em
SG200Δcda7
SG200Δcda7-cda7
H2O2 1.5 mM
H2O2 3.0 mM
PD
Caffeine 1.5 mM
SDS 0.01%
B
C
SG200
SG200cda3em
D
Width (μm)
Length (μm)
***

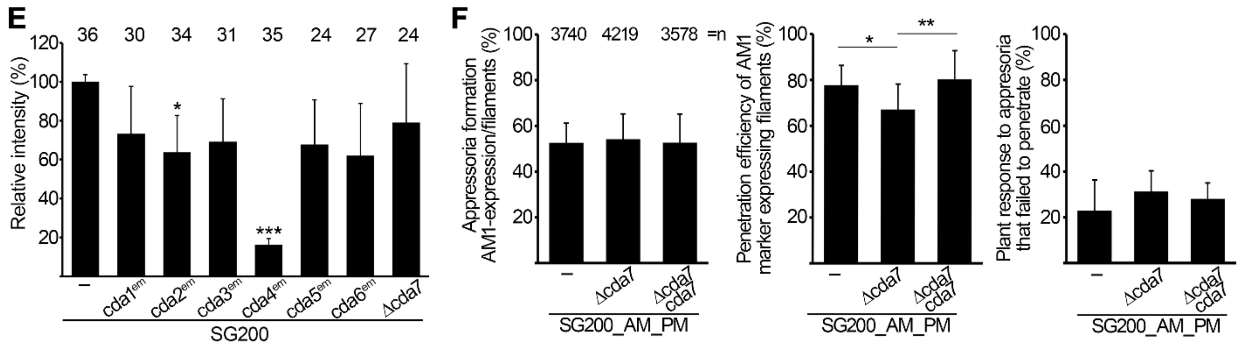

FIG S5.

Characterization of mutant strains lacking single *cda* genes. (A) Stress sensitivity of SG200, strains lacking single *cda* genes and the complemented strain SG200Δcda7-cda7. Serial 10-fold dilutions of cultures adjusted to OD_{600} of 1.0 were spotted on complete medium supplemented with 1% glucose in the absence of stressors (CM) or in the presence of stressors calcofluor, congo red, NaCl, sorbitol, H_2O_2 or in PD-agar in the absence (PD) or presence of the stressors caffeine or SDS. The plates were incubated at 28°C and pictures were taken after 2 d for CM, and PD, caffeine and SDS, 3 d for the plates containing calcofluor, congo red, or 1.5 mM H_2O_2, and 4 d for the plates containing NaCl, sorbitol, or 3 mM H_2O_2. (B) Morphology of colonies of single *cda* mutant strains. Microscopic pictures of single colonies of the indicated strains all grown on the same CM plate. Scale bar: 1 mm. (C) Spore germination of SG200 and SG200cda3^{em}. Microscopy was performed 48 hrs after spotting the spores on a PD-agar layer on microscopy slides and incubation at 28°C. Representative pictures are shown. Germinated spores are marked with arrowtips. Scale bar: 10 µm. (D) Width (right graphic) and length (left graphic) of budding cells of mutant strains lacking single *cda* genes. Three independent replicates each comprising 100 cell measurements were performed and are displayed by box-plot representation, the mean value is represented with an “x” inside of the box. Significant differences were determined with respect to SG200 by One-way ANOVA and Duncan multiple range test, (*, $P \leq 0.001$). (E) Quantification of relative fluorescence intensity of CAP-mKATE2 chitosan staining of biotrophic hyphae of SG200 and strains lacking single *cda* genes at 2 dpi. The average fluorescence intensity in a cross-section 5 µm from the hyphae tip was measured and normalized to the average fluorescence intensity of SG200 in the same experiment, which was set to 100. The average of four biological replicates are

shown. Numbers indicate the total number of hyphae analyzed per strain. Error bars represent ± SD. Significant differences were determined by two-side unpaired Student´s t-test compared to SG200 (*, $P \leq 0.05$; ***, $P \leq 0.001$). (F) Quantification of appressorium formation and successful penetration in the indicated strains with the help of promoters induced in appressoria (AM1 marker) and during penetration (PM marker) (Krombach S. Philipps University, Marburg, Hesse, Germany. 2016. doi:https://doi.org/10.17192/z2017.0051). For the quantification, infected maize seedlings were analyzed at 16 h post infection by confocal microscopy. Appressorium formation was determined as the number of filaments expressing the AM1 marker relative to the total number of filaments stained with calcofluor (left side panel), and penetration efficiency as number of filaments expressing the PM marker relative to the number of filaments expressing the AM1 marker (middle panel). The percentage of appressoria impaired in penetration associated to a plant defense response was determined as the number of filaments expressing the AM1 marker but failing in expression of the PM marker that were associated with calcofluor staining underneath the appresoria relative to the total number of filaments expressing the AM1 marker but not the PM marker (right side panel). In each biological replicate, 15 leaf areas from three leaves were evaluated per strain. Averages of six biological replicates are presented. Error bars indicate ± SD. Significant differences were determined by two-side unpaired Student´s t-test compared to SG200 (*, $P \leq 0.05$; **, $P \leq 0.01$).
