# Supplementary material for: Chitosan and Chitin Deacetylase Activity Are Necessary for Development and Virulence of Ustilago maydis
Source: mBio. 2021 Mar 2;12(2):e03419-20. doi: 10.1128/mBio.03419-20 (PMC8092297; doi:10.1128/mBio.03419-20)
Supplement: FIG S6 [file mBio.03419-20-sf006.pdf]

cda							Filamentation	Budding cells	Sensitivity to calcofluor	Chitosan content budding cells	Chitin content budding cells	Chitosan content biotrophic hyphae	Virulence	Adhesion	Appressoria formation	Penetration
1	2	3	4	5	6	7										
							normal	normal	normal	normal	normal	normal	normal	normal	normal	normal
1^{em}							normal	normal	normal	normal	normal	normal	normal	*n.a*	*n.a*	*n.a*
	2^{em}						normal	shorter	increased	decreased	increased	decreased	normal	*n.a*	*n.a*	*n.a*
		3^{em}					normal	normal	normal	normal	normal	normal	normal	*n.a*	*n.a*	*n.a*
			4^{em}				normal	normal	normal	decreased	increased	decreased	normal	*n.a*	*n.a*	*n.a*
				5^{em}			normal	normal	normal	normal	normal	normal	normal	*n.a*	*n.a*	*n.a*
					6^{em}		normal	normal	normal	normal	normal	normal	normal	*n.a*	*n.a*	*n.a*
						$\Delta 7$	decreased	normal	normal	normal	normal	normal	decreased	*n.a*	normal	decreased
1^{em}	2^{em}						normal	shorter wider	increased	decreased	increased	normal	normal	*n.a*	*n.a*	*n.a*
		3^{em}	4^{em}				normal	normal	normal	decreased	increased	decreased	normal	*n.a*	*n.a*	*n.a*
	2^{em}	3^{em}	4^{em}				decreased	shorter	increased	decreased	increased	decreased	decreased	*n.a*	*n.a*	*n.a*
	2^{em}	3^{em}	4^{em}	5^{em}	6^{em}		decreased	shorter wider	increased	decreased	increased	decreased	decreased	decreased	decreased	decreased
	2^{em}	3^{em}	4^{em}	5^{em}	6^{em}	$\Delta 7$	decreased	shorter wider	increased	decreased	increased	decreased	decreased	*n.a*	*n.a*	*n.a*
		3^{em}	4^{em}	5^{em}	6^{em}		normal	normal	normal	decreased	increased	decreased	normal	*n.a*	*n.a*	*n.a*
1^{em}		3^{em}	4^{em}	5^{em}	6^{em}		normal	normal	normal	decreased	increased	decreased	decreased	*n.a*	*n.a*	*n.a*
1^{em}		3^{em}	4^{em}	5^{em}	6^{em}	$\Delta 7$	decreased	normal	normal	decreased	increased	decreased	decreased	*n.a*	*n.a*	*n.a*

FIG S6.

Scheme listing the phenotype of the different *U. maydis cda* mutants generated for this study. Blue color intensity indicates the degree of reduction in mutants compared to wild type, intensity of orange indicates the degree by which a certain phenotype is increased in certain mutants and *n.a*. are conditions not analyzed.
