# Supplementary material for: Chitosan and Chitin Deacetylase Activity Are Necessary for Development and Virulence of Ustilago maydis
Source: mBio. 2021 Mar 2;12(2):e03419-20. doi: 10.1128/mBio.03419-20 (PMC8092297; doi:10.1128/mBio.03419-20)
Supplement: FIG S7 [file mBio.03419-20-sf007.pdf]

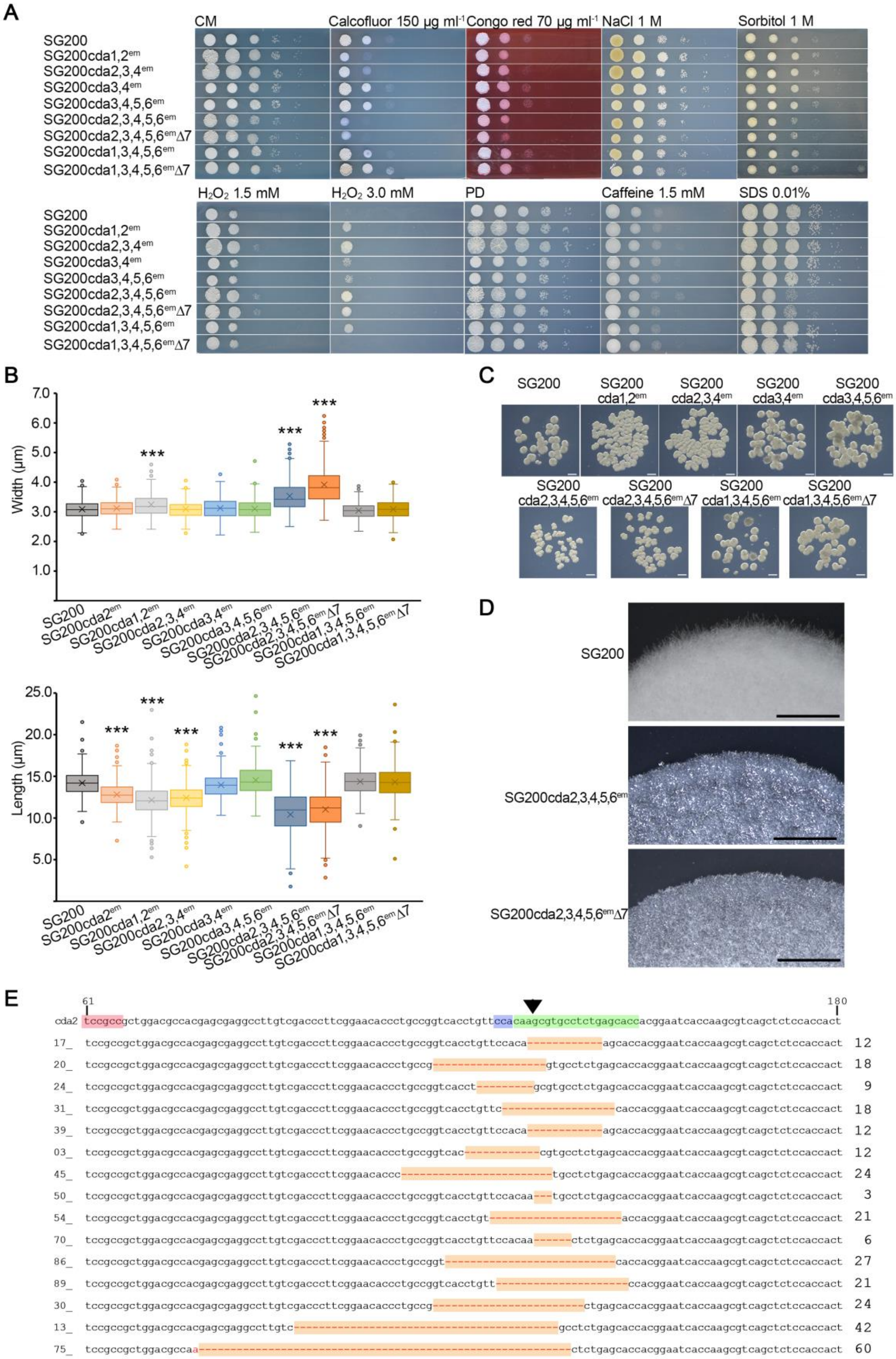
A
CM
Calcofluor 150 μg ml-1
Congo red 70 μg ml-1
NaCl 1 M
Sorbitol 1 M
SG200
SG200cda1,2em
SG200cda2,3,4em
SG200cda3,4em
SG200cda3,4,5,6em
SG200cda2,3,4,5,6em
SG200cda2,3,4,5,6emΔ7
SG200cda1,3,4,5,6em
SG200cda1,3,4,5,6emΔ7
H2O2 1.5 mM
H2O2 3.0 mM
PD
Caffeine 1.5 mM
SDS 0.01%
B
Width (μm)
Length (μm)
***
SG200
SG200cda2em
SG200cda1,2em
SG200cda2,3,4em
SG200cda3,4em
SG200cda3,4,5,6em
SG200cda2,3,4,5,6em
SG200cda2,3,4,5,6emΔ7
SG200cda1,3,4,5,6em
SG200cda1,3,4,5,6emΔ7
C
D
SG200
SG200cda2,3,4,5,6em
SG200cda2,3,4,5,6emΔ7
E
cda2
61
180

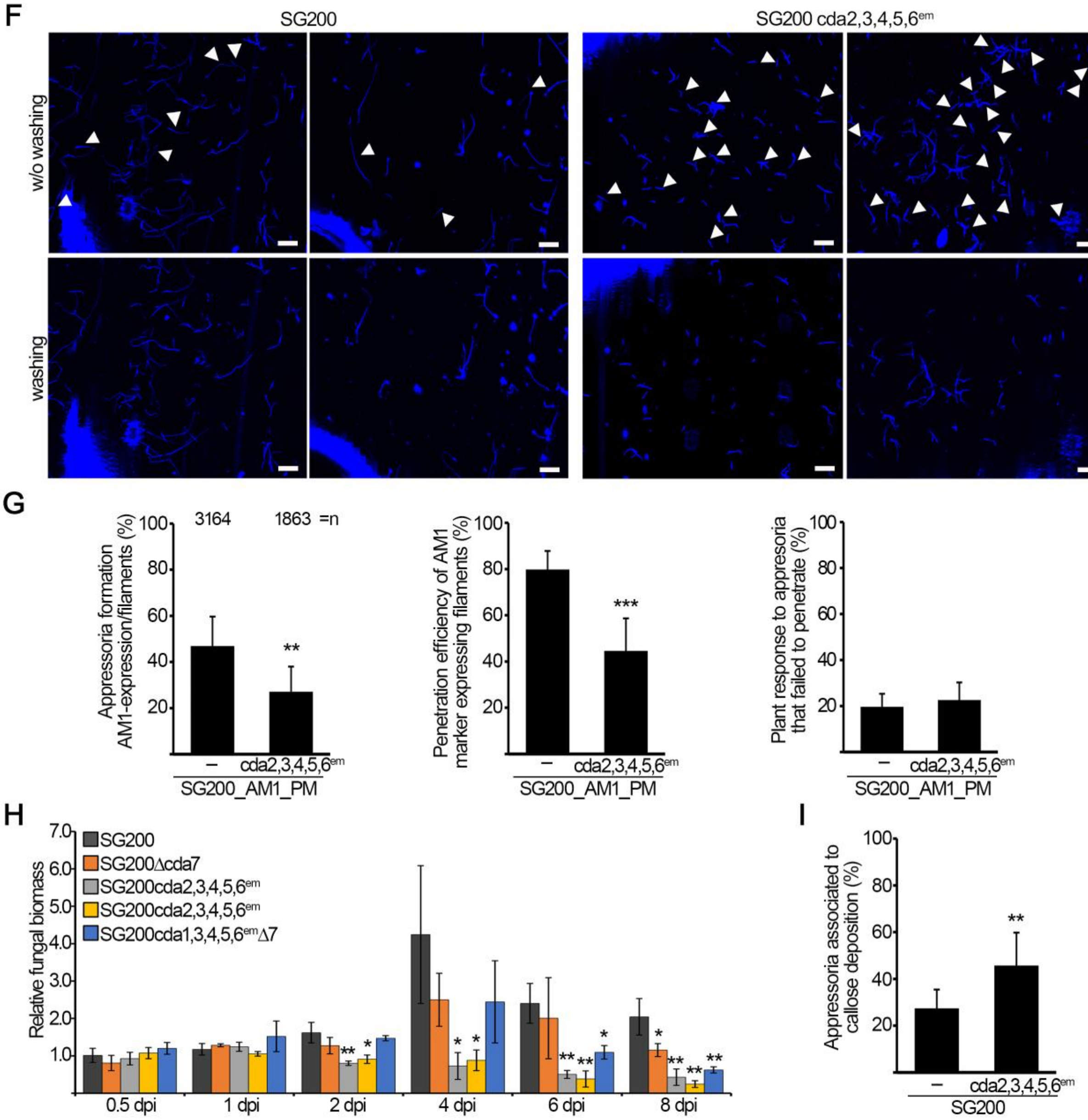
F
SG200
SG200 cda2,3,4,5,6em
w/o washing
washing
G
3164
1863 =n
Appressoria formation AM1-expression/filaments (%)
**
–
cda2,3,4,5,6em
SG200_AM1_PM
Penetration efficiency of AM1 marker expressing filaments (%)
***
Plant response to appresoria that failed to penetrate (%)
H
Relative fungal biomass
SG200
SG200Δcda7
SG200cda2,3,4,5,6em
SG200cda2,3,4,5,6em
SG200cda1,3,4,5,6emΔ7
0.5 dpi
1 dpi
2 dpi
4 dpi
6 dpi
8 dpi
I
Appressoria associated to callose deposition (%)
SG200

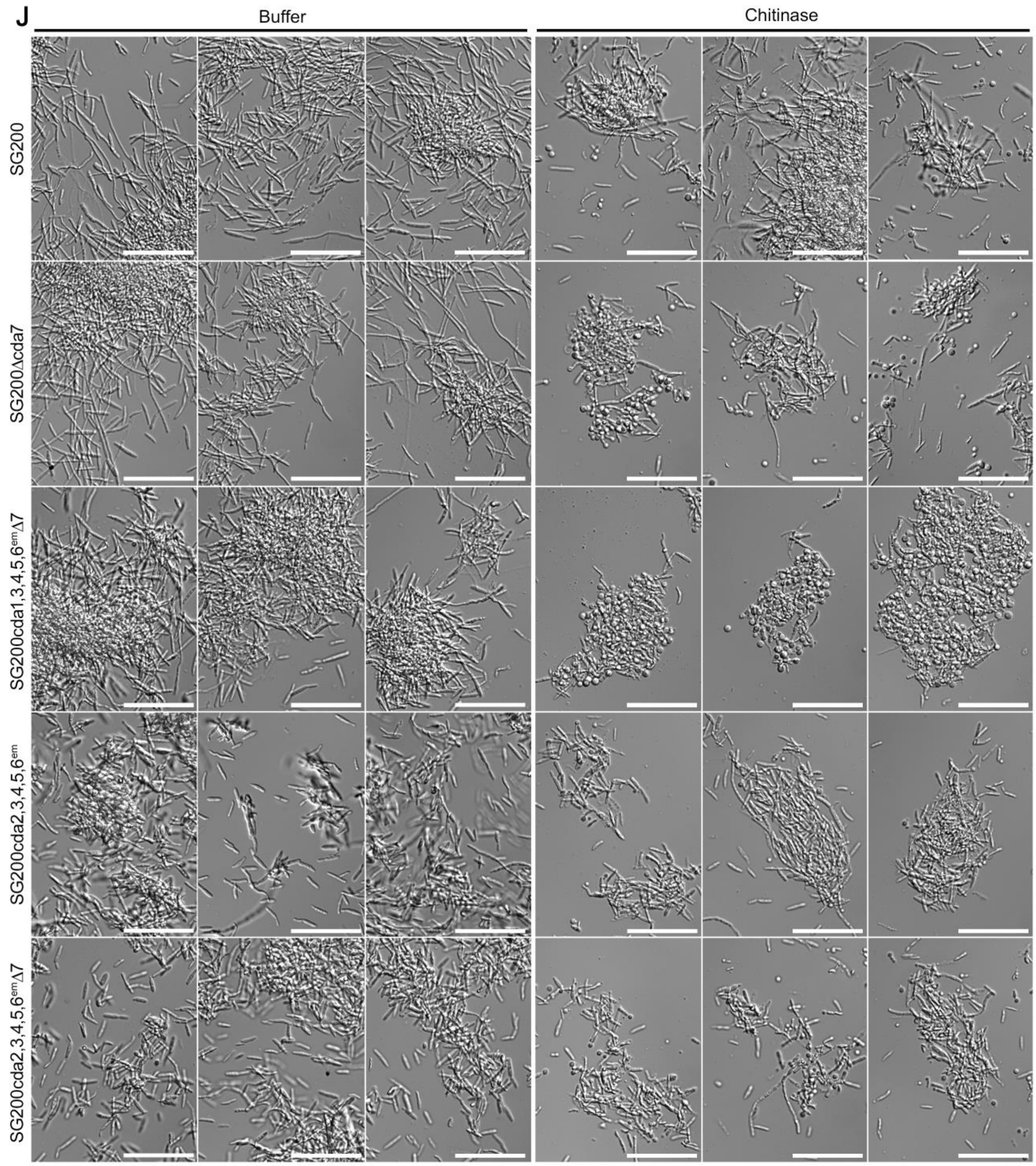

FIG S7.

Characterization of mutant strains lacking multiple *cda* genes. (A) Stress sensitivity of the indicated strains. Serial 10-fold dilutions of cultures adjusted to OD_{600} of 1.0 were spotted on complete medium supplemented with 1% glucose in the absence of stressors (CM) or in the presence of stressors calcofluor, congo red, NaCl, sorbitol, H_2O_2 or in PD-agar in the absence (PD)

or presence of the stressors caffeine or SDS. The plates were incubated at 28°C and pictures were taken after 2 d for CM, PD, caffeine and SDS, 3 d for the plate containing calcofluor, congo red, or 1.5 mM H_2O_2, and 4 d for the plates containing NaCl, sorbitol, or 3 mM H_2O_2. (B) Width (upper graphic) and length (lower graphic) of strains lacking multiple cda genes. Three independent replicates each comprising 100 cell measurements were performed and are displayed by box-plot representation, the mean value is represented with an "x" inside of the box. Significant differences were determined with respect to SG200 by One-way ANOVA and Duncan multiple range test, (*, $P \leq 0.001$). (C) Morphology of colonies of multiple *cda* mutant strains. Microscopic pictures of single colonies of the indicated strains all grown on the same CM plate. Scale bar: 1 mm. (D) Microscopic pictures of edges of colonies of SG200, SG200cda2,3,4,5,6^{em}; and SG200cda2,3,4,5,6^{em}Δ7; 10 µl of the cultures adjusted to OD_{600} of 1.0 were spotted on a PD-charcoal plate and photographed after 2 d of incubation. (E) Inactivation of *cda2* in SG200cda1,3,4,5,6^{em}Δ7 leads to in frame mutations. The top row shows the nucleotide sequence of *cda2* between nucleotides 61 and 180. The target for the sgRNA is indicated in green, protospacer-adjacent motif (PAM) sequence is indicated in blue and the expected cleavage site is indicated by an arrowhead. The 3´end of the sequence encoding the signal peptide is indicated in pink. In total, 96 mutants were analyzed and 15 representative sequences are show. The deleted parts are given in orange. Numbers to the right indicate how many nucleotides were deleted. (F) Adherence of the hyphae to the leaf surface. Adherence was analyzed in leaves infected with the indicated strains 12 h post infection. The leaf samples were stained with calcofluor and observed by confocal microscopy either before washing or after washing in water containing 0.1% tween 20. Cells which have disappeared after the washing step are marked with white arrow tips. Scale bar: 50 µm. (G) Quantification of appressorium formation and successful penetration in the indicated strains expressing the appressorial marker AM1 and the penetration marker PM. For the quantification, infected maize seedlings were analyzed at 16 h post infection by confocal microscopy. Appressorium formation was determined as the number of filaments expressing the AM1 marker relative to the total number of filaments stained with calcofluor (left side panel) and penetration efficiency as number of filament expressing the PM marker relative to the number of filaments expressing the AM1 marker (middle panel). Defense responses associated with calcofluor staining underneath the appressoria were determined by identifying appressoria impaired in penetration (filaments expressing the AM1 marker but not expressing the PM marker)

and relating this in % to the total number of appressoria impaired in penetration (right side panel). In each biological replicate, 15 leaf areas from three leaves were evaluated per strain. Averages of four biological replicates are presented. Error bars indicate ± SD. Significant differences were determined by two-side unpaired Student´s t-test compared to SG200. (**, $P \leq 0.01$, ***, $P \leq 0.001$). (H) Relative fungal biomass was determined by qPCR. For this, genomic DNA was prepared at 0.5, 1, 2, 4, 6, and 8 dpi from maize leaves infected with the indicated strains. The fungal gene *ppi* and the plant gene *gapdh* were used for estimating relative fungal biomass. SG200 biomass at 0.5 dpi was set to 1. Average values of three biological replicates are shown. Error bars indicate ± SD. Significance of differences between mutant strains and SG200 at each time point was calculated by Student's t-test (*, $P \leq 0.05$; **, $P \leq 0.01$). (I) Percentage of appressoria eliciting callose deposition. At 2 dpi the appressoria on the leaf surface of the indicated strains were identified after calcofluor staining and callose deposition was visualized by co-staining with aniline blue. The percentage of appressoria that induced callose accumulation was determined. Five leaf areas from two leaves per strain were analyzed and between 92 and 283 appressoria for SG200 and between 20 and 58 appressoria for SG200cda2,3,4,5,6^{em} were studied. Average values of five biological replicates are shown. Error bars indicate ± SD. Significance of difference was calculated by Student's t-test (**, $P \leq 0.01$). (J) Chitinase treatment on filaments of selected *cda* mutants. The strains indicated on the left were grown for 24 h on PD-charcoal plates to induce filamentation and subsequently removed, treated for 1 h with chitinase and observed by microscopy. Representative pictures are shown and the experiment was repeated three times with similar results. Scale bar: 50 µm.
