# Supplementary material for: Chitosan and Chitin Deacetylase Activity Are Necessary for Development and Virulence of Ustilago maydis
Source: mBio. 2021 Mar 2;12(2):e03419-20. doi: 10.1128/mBio.03419-20 (PMC8092297; doi:10.1128/mBio.03419-20)
Supplement: FIG S8 [file mBio.03419-20-sf008.pdf]

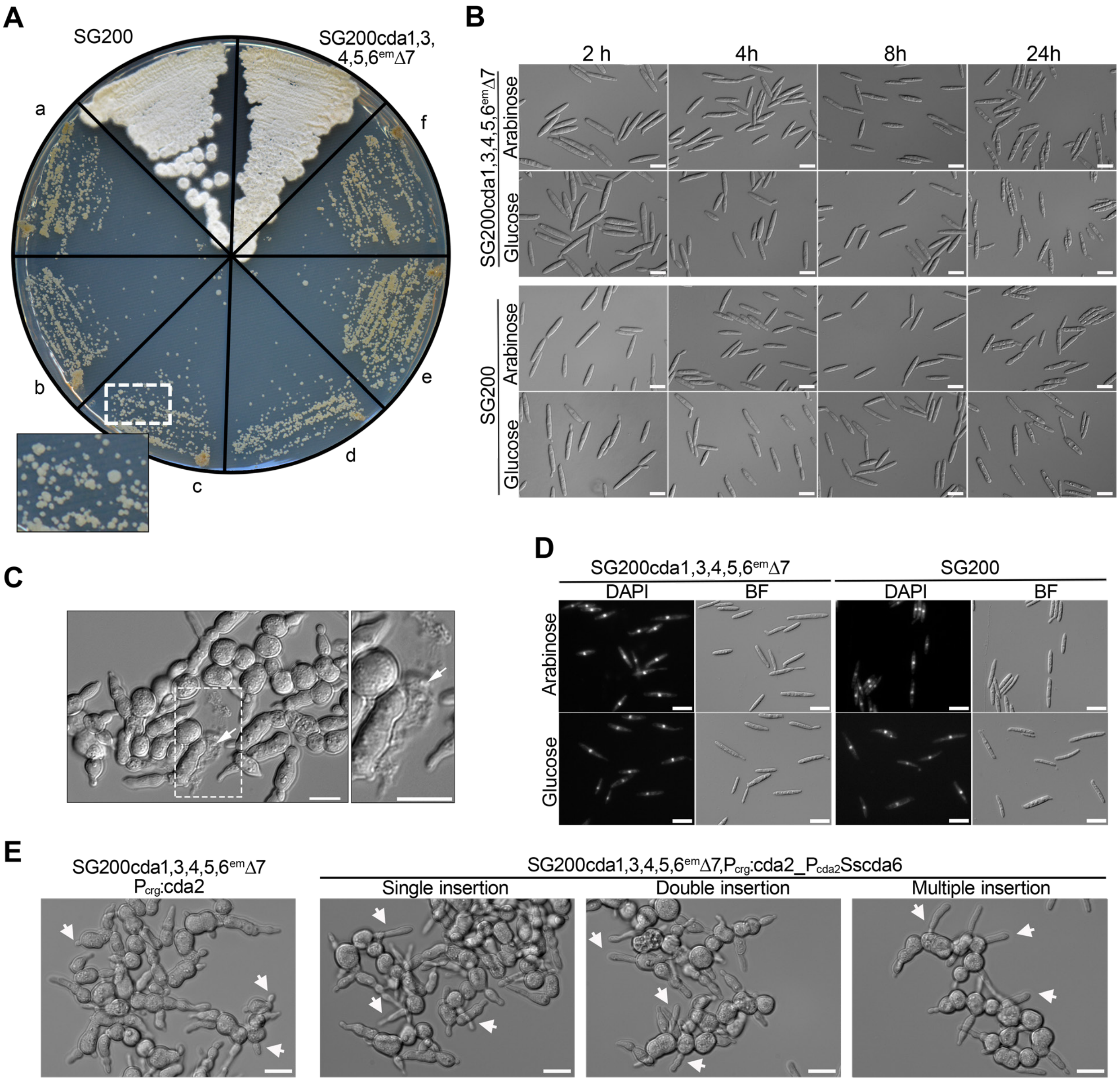

FIG S8.

Viability of *U. maydis* strains lacking all *cda* genes. (A) Six single colonies labeled (a) to (f) of SG200cda1,3,4,5,6^{em}Δ7,P$_{crg}$:cda2 from the CM-medium plate containing 1% glucose shown in **Fig. 7A** were re-streaked on CM medium containing 1% glucose and incubated for 4 d. Colonies of SG200 and SG200cda1,3,4,5,6^{em}Δ7 were streaked as controls. The enlargement from (c) shows colony heterogeneity indicative of the emergence of suppressor mutations. (B) Cell disintegration in SG200cda1,3,4,5,6^{em}Δ7_P$_{crg}$:cda2. After 24 h in glucose-containing medium, disintegrating cells can be observed (white arrow). (C) Cell morphology of SG200 and SG200cda1,3,4,5,6^{em}Δ7.

Indicated strains were initially grown in CM-liquid medium containing 1% arabinose, and after adjusting the OD_{600} to 0.2 shifted CM-liquid containing 1% arabinose as control (left column). The cultures were observed microscopically at 2, 4, 8, and 24 h post shift. This assay serves as control for Figure 7B. (D) Nuclei in SG200cda1,3,4,5,$6^{em}\Delta7$ which served as precursor for the depletion of Cda2. DAPI staining of the indicated strains 24 h after the shift to CM-liquid medium containing 1% arabinose or CM-medium containing 1% glucose. This figure serves as control for Figure 7C. (E) Cell morphology of SG200cda1,3,4,5,$6^{em}\Delta7$,P_{crg}:cda2 containing P_{cda2}*Sscda6* in single or multiple copies after 24 h of growth in CM-liquid medium containing 1% glucose. Emerging cigar-shaped cells whose length is increasing with copy number of P_{cda2}*Sscda6* are indicated (white arrows). SG200cda1,3,4,5,$6^{em}\Delta7$,P_{crg}:cda2 grown under the same conditions is shown as control.
