# Supplementary material for: Chitosan and Chitin Deacetylase Activity Are Necessary for Development and Virulence of Ustilago maydis
Source: mBio. 2021 Mar 2;12(2):e03419-20. doi: 10.1128/mBio.03419-20 (PMC8092297; doi:10.1128/mBio.03419-20)
Supplement: TEXT S1 [file mBio.03419-20-s0001.pdf]

Supplementary Information for:

Chitosan and chitin deacetylase activity are necessary for development and virulence of *Ustilago maydis*

Yanina S. Rizzi[1], Petra Happel[1], Sandra Lenz[1], Mounashree J. Urs[2], Martin Bonin[2], Stefan Cord-Landwehr[2], Ratna Singh[2], Bruno M. Moerschbacher[2] and Regine Kahmann[1#]

[1]Max Planck Institute for Terrestrial Microbiology, Dept. Organismic Interactions, Karl-von-Frisch Straße 10, 35043 Marburg, Germany

[2]Westfälische Wilhelms-Universität Münster, Institut für Biologie und Biotechnologie der Pflanzen, Schlossplatz 8, 48143 Münster, Germany

#Corresponding author

Text S1. Supplementary Materials and Methods

Media and buffers

dYT medium: 1.6% tryptone, 1% yeast extract, 0.5% NaCl.

LB medium: 1.0% Tryptone, 0.5% yeast extract, 1.0% NaCl.

YEPSL medium: 0.4% yeast extract, 0.4% peptone, 2% sucrose.

Solid-PD medium: 3.9 % PD agar, 10 mM Tris-HCl, pH 8.0.

PBS buffer: 137 mM NaCl, 2.7 mM KCl, 10 mM Na_2HPO_4, 1.8 mM KH_2PO_4, pH 7.4.

Media M: 1.25 M NaH_2PO_4, 1.25 M KH_2PO_4, 2.5 M NH_4Cl, 0.25 M Na_2SO_4.

Media 5052: 25% glycerol, 10% α-lactose monohydrate, 2.5% D-glucose.

FPLC-washing buffer: 20 mM triethanolamine (TEA), 400 mM NaCl, pH 8.0.

High salt buffer: 1 M TEA, 1 M NaCl, pH 8.5.

Digestion solution (Ma LS, Wang L, Trippel C, Mendoza-Mendoza A, Ullmann S, Moretti M, Carsten A, Kahnt J, Reissmann S, Zechmann B, Bange G, Kahmann R. Nat Commun 9:1711, 2018): 10 mM MES pH 5.7, 1.5% cellulase Onozuka R-10 from *Trichoderma viride* (SERVA GmbH), 0.3% macerozyme R-10 from *Rhizopus* sp. (SERVA GmbH), 0.6 M mannitol, 1 mM $CaCl_2$, 0.1% BSA.

Growth, filamentation and stress assay

For growth, filamentation and stress assays of *U. maydis*, strains were grown to an OD_{600}=1 in YEPSL following (Krombach S, Reissmann S, Kreibich S, Bochen F, Kahmann R. New Phytol 220:553-566. 2018). The cells were harvested by centrifugation and diluted in H_2O to a final OD_{600}=1.0. 10 µl of the suspension were spotted on PD-plates to assay for growth, or on PD-charcoal plates (PD-agar containing 1% activated charcoal) to assay the formation of aerial hyphae (Day PR, Anagnostakis SL, Puhalla JE. Proc Natl Acad Sci U S A 68:533-5, 1971). For stress assays, serial dilutions of cultures adjusted to an OD_{600}=1.0 were spotted on CM-agar supplemented with 1% glucose, with or without the addition of 150 µg ml^{-1} calcofluor, 70 µg ml^{-1} congo red, 1 M NaCl, 1 M sorbitol or H_2O_2 (1.5 mM or 3.0 mM), or PD-plates with or without the addition of 0.01% SDS or 1.5 mM caffeine.

UHPLC-ESI-MS analysis

The resulting products of *U maydis* CDAs activity assays were analysed through UHPLC-ESI-MS[1] using Dionex Ultimate 3000RS UHPLC system (Thermo Fisher Scientific) coupled to amaZon speed ESI-MS^n detector (Bruker Daltonik GmbH) as previously was performed by (Hamer SN, Cord-Landwehr S, Biarnés X, Planas A, Waegeman H, Moerschbacher BM, Kolkenbrock S. Sci Rep 5:8716. 2015). The reaction products were separated by VanGuared precolumn (1.7 µm, 2.1 × 5 mm) and Acquity UHPLC BEH amide column (1.7 µm, 2.1 × 150 mm; Waters GmbH) through hydrophilic interaction chromatography (HILIC) with solvent A (80% acetonitrile and 20% water) and solvent B (20% acetonitrile and 80% water), both containing 10 mM NH_4HCO_2 and 0.1% formic acid. Solvent B gradient is increased from 0% to 70% (0.8 – 3.3 min), followed by 70% solvent B (3.3 – 4.3 min), and then a decrease from 70% to 0% (4.3 – 4.5 min), finally reaching 100% of solvent A. The separation was performed at a column temperature of 75°C and a flow rate of 0.8 ml/min. MS detection was performed in positive mode and mass spectra were acquired over a scan range of m/z of 50 to 2,000 in enhanced resolution scan mode.

CDA and chitinase activity on polymeric substrate

A dot activity gel assay was performed to determine the enzyme activity of the CDAs on a soluble chitin derivative, glycol-chitin, as described previously (Govinda Rajulu MB, Thirunavukkarasu N, Suryanarayanan TS, Ravishankar JP, El Gueddari NE, Moerschbacher BM. Fungal Diversity

47:43-53, 2011). Briefly, a glycol-chitin solution (1%) was homogeneously incorporated into an acrylamide gel and 5 µl droplets of the purified enzymes (Cda1, 2, 4, 5: 500 ng; Cda3: 615 ng; Cda7: 545 ng) in 50 mM TEA buffer at pH 7, using recombinant *Cl*CDA (500 ng) as a positive control, and water as well as the TEA buffer at pH 7 buffer as negative control, were placed on the solidified gel surface and incubated overnight in a humid chamber at 37°C. To detect potential chitinase activity, the gel was stained for 10 min using 0.01 % calcofluor visualized under UV transillumination (312 nm). Chitinase activity becomes visible as dark spots on the gel. To detect CDA activity, the glycol-chitosan formed in the gel by CDA-catalyzed deacetylation was depolymerized using a mixture containing sulfuric acid and sodium nitrite (500 µl concentrated sulfuric acid added to 20 ml H_2O containing 7.59 g $NaNO_2$)and visualized under UV transillumination (312 nm). The CDA activity became apparent by the formation of dark spots.

***cda* gene expression in teliospores**

To collect teliospores, cobs of 35-days-old plants of the variety Gaspé Flint were infected with 3 ml of a 1:1 mix of FB1 and FB2 grown in YEPSL medium to an OD_{600}=0.8-1.2 and resuspended in water to an OD_{600}=2. At 15 dpi, cobs that had developed tumors were collected, cut in small pieces, and dried at 37°C. After suspension in water, the material was dispersed with a mixer (Polytron PT 1300, Lucerne, Switzerland). The suspension was filtered using a cell strainer of Nylon 40 µm pore (Corning). The spore suspension was washed eight to ten times with sterile water. Spore germination in PD liquid medium followed (Donaldson ME, Ostrowski LA, Goulet KM, Saville BJ. 2017. BMC Genomics 18:340. 2017).

To determine *cda* gene expression in spores and germinating spores, total RNA was isolated from 200 mg of spores and germinated spores collected as described above, following (Zahiri AR, Babu MR, Saville BJ. Molecular Genetics and Genomics 273:394-403. 2005). RNA isolated from infected leaf tissue following (Lanver D, Muller AN, Happel P, Schweizer G, Haas FB, Franitza M, Pellegrin C, Reissmann S, Altmuller J, Rensing SA, Kahmann R. Plant Cell 30:300-323. 2018) served as control. qRT-PCR was performed as described (Brefort T, Tanaka S, Neidig N, Doehlemann G, Vincon V, Kahmann R. PLoS Pathog 10:e1003866. 2014) using primers described in **Table S1C**. The peptidylprolylisomerase (*ppi, UMAG_03726*) amplified with primers SR135 and SR136 served as control see **Table S1C**. Expression of the *cda* genes relative to *ppi* was determined by the $2^{-\Delta Ct}$ method (Livak KJ, Schmittgen TD. Methods 25:402-8 2001).

Teliospore germination and viability

To enrich spores from infected tissue, developing cobs of the maize variety Gaspe Flint were infected with SG200 and SG200cda3^{em} to obtain sufficient spore numbers. Infected cobs were harvested 15 days post infection, cut into small pieces and dried at 28°C. 100 mg of dried tumor material was disintegrated in a mortar to release spores, resuspended in water and filtered through a 40 µm Nylon filter to remove large pieces of cell debris. The flow through was washed four times with water and centrifugations at 1000 rpm. The enriched spores were treated with 1.5 % $CuSO_4$ for 15 min to kill remaining hyphae followed by washing three times with water. The spores were suspended in water containing tetracycline (25 µg/ml) and ampicillin (100 µg/ml). For microscopy, this suspension was spotted on microscopy slides covered with PD-agar and kept in a wet chamber at 28°C for 48h. To determine spore viability, the spore concentration of the suspension was determined microscopically prior to plating on PD plates containing ampicillin (100 µg/ml), kanamycin (40 µg/ml) and chloramphenicol (34 µg/ml) and incubated at 28°C for 72 hrs. Three independent infections were done and the percentage of spores forming colonies was determined. Significant differences were determined by two-side unpaired Student´s t-test.

Fungal biomass determination

To determine fungal biomass after infection, 7-day-old maize seedlings were inoculated with SG200, SG200Δcda7, SG200cda2,3,4,5,6^{em}; SG200cda2,3,4,5,6^{em}Δ7; or SG200cda1,3,4,5,6^{em}Δ7. Sections from the third leaf 1 cm below the injection holes, extending and including the leaf sheath up to the ligula of the second leaf, were collected from 15 plants for time points 0.5, 1, and 2 dpi, and from ten plants for 4, 6, and 8 dpi. Immediately after collection, leaves were rinsed in water, dried with paper, and frozen in liquid nitrogen. Genomic DNA extraction and the quantification of relative fungal biomass in infected maize leaves was performed as described (Brefort T, Tanaka S, Neidig N, Doehlemann G, Vincon V, Kahmann R. PLoS Pathog 10:e1003866. 2014) using the *ppi*-specific primers SR135 and SR136 to quantify *U. maydis* biomass and as reference for normalization the maize glyceraldehyde dehydrogenase (*gapdh*) amplified with primers SR137 and SR138 (**Table S1C**). Relative fungal biomass values were calculated with the $2^{-\Delta\Delta Ct}$ method (Livak KJ, Schmittgen TD. Methods 25:402-8 2001).

Quantification of chitosan staining in biotrophic hyphae

To quantify the intensity of CAP staining in leaves infected with SG200 and strains inactivated in single *cda* genes, the samples were processed for CAP staining, and microscopic pictures of hyphae completely released from the plant tissue were selected, and conditions between samples were kept constant. The intensity of the fluorescence signal was measured in a cross-section at a distance of 5 µm from the tip using ImageJ. 5 to 11 hyphae were measured per strain per replicate. Four independent replicates were performed.

Appressoria formation and penetration efficiency

To quantify appressoria formation and penetration efficiency, strains were used which express the appressorial marker AM1 (Mendoza-Mendoza A, Berndt P, Djamei A, Weise C, Linne U, Marahiel M, Vranes M, Kamper J, Kahmann R. Mol Microbiol 71:895-911. 2009) as well as the penetration marker PM (Krombach S. Philipps-University, Marburg, Hesse, Germany. 2016 https://doi.org/10.17192/z2017.0051). To this end third leaves were harvested 16 h after plant inoculation, stained in 10 µg ml^{-1} calcofluor white, and mounted on a microscopic slide. For quantification, five pictures of three independent leaves were taken for each strain. First, the total number of filaments stained with calcofluor were counted per photographed area. Second, the number of filaments expressing the AM1 marker (GFP fluorescence) as indicator for appressoria formation were counted, and third, the filaments expressing the PM marker (mCherry fluorescence) as marker of successful penetration were counted. Appressoria formation was determined as the ratio between filaments expressing GFP and total filaments. Penetration efficiency was determined as the ratio of filaments expressing mCherry relative to filaments expressing GFP. To determine whether appressoria which failed to penetrate elicited plant defense responses, appressoria expressing the AM1 appressorial marker but failed to express the PM penetration marker were first identified and in a second step it was determined which percentage of these hyphae elicit calcofluor staining underneath the appressoria as sign of defense responses.

Adherence of fungal hyphae to the leaf surface

To determine adherence, samples of the third leaf of infected plants were collected at 0.5 dpi, and 2 cm sections 1 cm below the injection holes were excised. Leaf segments were placed on a cover slip to which 100 µl of 5 µg ml^{-1} calcofluor white had been applied, followed by placing the

microscopy slide on top. After visualization by confocal microscopy, the leaf samples were washed in 20 ml of water containing 0.1% tween 20, for 1 min with vortexing. Then, the sample was remounted and observed again by confocal microscopy.

Measurement of width and length of budding cells

Measurement of width and length of budding cells was performed as described in (Elias-Villalobos A, Fernandez-Alvarez A, Moreno-Sanchez I, Helmlinger D, Ibeas JI. PLoS Pathog 11:e1005134. 2015).

Callose deposition

Callose deposition detected with aniline blue staining followed (Herrberger C, Zechmann B, Hillmer M, Doehlemann G. PLoS Pathog 8:e1002684. 2012). Samples were co-stained with calcofluor and observed by confocal microscopy. The total number of appressoria was determined after calcofluor staining and identifying hyphae that show the characteristic swelling at hyphal tips associated with curving of the hypha behind (Snetselaar KM, Mims CW. Phytopathology 83:843-850. 1993). The percentage of appressoria which had induced callose deposition was determined in merged pictures.

Chitinase treatment

To analyze the susceptibility to chitinase, cultures of the different strains were grown to an OD_{600} of about 1.0 in YEPSL, the OD_{600} was adjusted to 1.0 in H_2O and then 5 µl of the suspension were spotted on PD-charcoal plates. After 24 h of growth, three filamentous colonies were transferred to a tube, washed with water and resuspended in 100 µl 20 mM sodium citrate, pH 5.8, containing 1 M sorbitol. Samples were split in two and either the same volume of buffer was added or buffer containing 0.21 µg ml^{-1} of chitinase from *Trichoderma viridae* (≥600 units g^{-1}, Sigma-Aldrich) was added. After 1 h of treatment at room temperature, the samples were analyzed by microscopy.
