# Supplementary material for: Chitosan and Chitin Deacetylase Activity Are Necessary for Development and Virulence of Ustilago maydis
Source: mBio. 2021 Mar 2;12(2):e03419-20. doi: 10.1128/mBio.03419-20 (PMC8092297; doi:10.1128/mBio.03419-20)
Supplement: TABLE S1 [file mBio.03419-20-st001.pdf]

**Table S1** Strains, plasmids, oligonucleotides, double stranded DNA fragments and accession numbers

**Table S1A** Strains used in the study

| **Name** | **Number** | ***Genotype*** | **Progenitor** | **Resistance**[a] | **Plasmid name** | **Comments** |
|---|---|---|---|---|---|---|
| SG200 | | *a1 mfa2 bW2 bE1* | | P | | Kamper J, Kahmann R, Bolker M, Ma LJ, et al. Nature 444:97-101. 2006. |
| FB1 | | *a1b1* | | | | Banuett F, Herskowitz I. Proc Natl Acad Sci U S A 86:5878-82.1989. |
| FB2 | | *a2b2* | | | | Banuett F, Herskowitz I. Proc Natl Acad Sci U S A 86:5878-82.1989. |
| SG200cda1[em] | PH249 | *a1 mfa2 bW2 bE1, ble; cda1Δ112-121* | SG200 | P | pSL5 | This study |
| SG200cda2[em] | PH285 | *a1 mfa2 bW2 bE1, ble; cda2Δ123-133* | SG200 | P | pSL6 | This study |
| SG200cda3[em] | PH257 | *a1 mfa2 bW2 bE1, ble; cda3Δ23-53* | SG200 | P | pSL3 | This study |
| SG200cda4[em] | PH336 | *a1 mfa2 bW2 bE1, ble; cda4Δ152-153* | SG200 | P | pPH40 | This study, in addition to the 2 bp deletion the mutant carries *cda4* base substitutions (wild type sequence actacagcttta is changed to ac--tagggtta) |
| SG200cda5[em] | PH390 | *a1 mfa2 bW2 bE1, ble; cda5Δ104-107* | SG200 | P | pPH42 | This study |
| SG200cda6[em] | PH375 | *a1 mfa2 bW2 bE1, ble; cda6Δ58-284* | SG200 | P | pPH41 | This study |

| | | | | | | |
|---|---|---|---|---|---|---|
| SG200Δcda7 | YR472 | *a1 mfa2 bW2 bE1, ble; cda7::hyg* | SG200 | P, HY | pYR103 | This study |
| SG200Δcda7,cda7 | YR506 | *a1 mfa2 bW2 bE1, ble; cda7::hyg ipR [Pcda7:cda7]ipS* | SG200 | P, HY, C | pYR122 | This study |
| SG200cda1,2$^{em}$ | PH355 | *a1 mfa2 bW2 bE1, ble; cda1Δ112-121; cda2Δ122-134* | PH249 | P | pSL6 | This study |
| SG200cda2,3,4$^{em}$ | SL7 | *a1mfa2 bW2bE1 cda2in+1; cda3Δ22 cda4Δ145-158* | SG200 | P | pSL3 | This study, the 1 bp insertion in *cda2* is A inserted between positions 129 and 130 |
| SG200cda3,4$^{em}$ | PH280 | *a1 mfa2 bW2 bE1, ble; cda3Δ21-28; cda4Δ146-155* | SG200 | P | pSL3 | This study, in addition to the 10 bp deletion in *cda4*, the nucleotide A at position 144 is exchanged to G |
| SG200cda3,4,5,6$^{em}$ | PH369 | *a1 mfa2 bW2 bE1, ble; cda3Δ21-28; cda4Δ146-155; cda5 Δ101-113; cda6 Δ74-83* | PH280 | P | pPH39 | This study, in addition to the 10 bp deletion in *cda4*, the nucleotide A at position 144 is exchanged to G |
| SG200cda1,3,4,5,6$^{em}$ | PH422 | *a1 mfa2 bW2 bE1, ble; cda1Δ121-127; cda3Δ21-28; cda4Δ146-155; cda5Δ101-113; cda6Δ74-83* | PH369 | P | pPH38 | This study, in addition to the 10 bp deletion in *cda4*, the nucleotide A at position 144 is exchanged to G |
| SG200cda1,3,4,5,6$^{em}$Δ7 | YR457 | *a1 mfa2 bW2 bE1, ble; cda1Δ121-127; cda3Δ21-28; cda4Δ146-155; cda5Δ101-113; cda6Δ74-83; cda7::hyg* | PH422 | P, H | pYR103 | This study, in addition to the 10 bp deletion in *cda4*, the nucleotide A at position 144 is exchanged to G |

| | | | | | | |
|---|---|---|---|---|---|---|
| SG200cda2,3,4,5,$6^{em}$ | PH418 | *a1 mfa2 bW2 bE1, ble; cda2Δ121-134; cda3Δ21-28; cda4Δ146-155; cda5 Δ101-113; cda6 Δ74-83* | PH369 | P | pPH43 | This study, in addition to the 10 bp deletion in *cda4*, the nucleotide A at position 144 is exchanged to G |
| SG200cda2,3,4,5,$6^{em}\Delta7$ | YR464 | *a1 mfa2 bW2 bE1, ble; cda2Δ121-134; cda3Δ21-28; cda4Δ146-155; cda5 Δ101-113; cda6 Δ74-83;cda7::hyg* | PH418 | P, H | pYR103 | This study, in addition to the 10 bp deletion in *cda4*, the nucleotide A at position 144 is exchanged to G |
| SG200cda1,3,4,5,$6^{em}\Delta7,P_{crg}$:cda2 | PH464 | *a1 mfa2 bW2 bE1, ble; cda1Δ121-127; cda3Δ21-28; cda4Δ146-155; cda5Δ101-113; cda6Δ74-83; cda7::hyg; Pcda2(D-1533 to -1) :: Pcrg:cda2* | YR457 | P, H | pPH45 + pSL6 | This study, in addition to the 10 bp deletion in *cda4*, the nucleotide A at position 144 is exchanged to G, and in this strain the promoter of *cda2* is substituted by the *U. maydis crg1* promoter |
| SG200cda1,3,4,5,$6^{em}\Delta7,P_{crg}$:cda2,$P_{cda6}$:cda6 | PH493 | *a1 mfa2 bW2 bE1, ble; cda1Δ121-127; cda3Δ21-28; cda4Δ146-155; cda5Δ101-113; cda6Δ74-83; cda7::hyg; Pcda2(D-1533* to *-1) :: Pcrg:cda2; ipR[Pcda6:cda6:Tnos]ipS* | PH464 | P, C | pPH49 | This study, in addition to the 10 bp deletion in cda4, the nucleotide A at position 144 is exchanged to G, and in this strain the promoter of *cda2* is substituted by the *U. maydis crg1* promoter. *cda6*is inserted in double copy |

| | | | | | | |
|---|---|---|---|---|---|---|
| SG200cda1,3,4,5,6$^{em}$Δ7,$P_{crg}$:cda2, $P_{cda2}$:sscda6 | PH502 | *a1 mfa2 bW2 bE1, ble; cda1Δ121-127; cda3Δ21-28; cda4Δ146-155; cda5Δ101-113; cda6Δ74-83; cda7::hyg; Pcda2(D-1533 to -1) :: Pcrg:cda2; ipR[Pcda2:sscda6:Tnos]ipS* | PH464 | P, C | pPH50 | This study, in addition to the 10 bp deletion in *cda4*, the nucleotide A at position 144 is exchanged to G, and in this strain the promoter of *cda2* is substituted by the *U. maydis crg1* promoter. *sscda6* is inserted in single copy |
| SG200cda1,3,4,5,6$^{em}$Δ7,$P_{crg}$:cda2, $P_{cda2}$:sscda6 | PH504 | *a1 mfa2 bW2 bE1, ble; cda1Δ121-127; cda3Δ21-28; cda4Δ146-155; cda5Δ101-113; cda6Δ74-83; cda7::hyg; Pcda2(D-1533 to -1) :: Pcrg:cda2; ipR[Pcda2:sscda6:Tnos]ipS* | PH464 | P, C | pPH50 | This study, in addition to the 10 bp deletion in *cda4*, the nucleotide A at position 144 is exchanged to G, and in this strain the promoter of *cda2* is substituted by the *U. maydis crg1* promoter. *sscda6* is inserted in double copy |
| SG200cda1,3,4,5,6$^{em}$Δ7,$P_{crg}$:cda2, $P_{cda2}$:sscda6 | PH507 | *a1 mfa2 bW2 bE1, ble; cda1Δ121-127; cda3Δ21-28; cda4Δ146-155; cda5Δ101-113; cda6Δ74-83; cda7::hyg; Pcda2(D-1533 to -1) :: Pcrg:cda2; ipR[Pcda2:sscda6:Tnos]ipS* | PH464 | P, C | pPH50 | This study, in addition to the 10 bp deletion in *cda4*, the nucleotide A at position 144 is exchanged to G, and in this strain the promoter of *cda2* is substituted by the *U. maydis crg1* promoter. *sscda6* is inserted in multiple copy |
| SG200_$P_{act}$cda3_StrepII-tag | YR531 | *a1mfa2 bW2bE1 ipR[Pact_cda3(w/o-GPI):StrepII-* | SG200 | P, C | pYR124 | This study, *cda3* is inserted in single copy |

| | | | | | | |
|---|---|---|---|---|---|---|
| | | *tag]ipS* | | | | |
| SG200_$P_{act}$cda3_ StrepII-tag | YR532 | *a1mfa2 bW2bE1 ipR[Pact_cda 3(w/o-GPI):StrepII-tag]ipS* | SG200 | P, C | pYR124 | This study, *cda3* is inserted in multiple copy |
| SG200_$P_{act}$cda6_ StrepII-tag | YR537 | *a1mfa2 bW2bE1 ipR[Pact_cda 6(w/o-GPI):StrepII-tag]ipS* | SG200 | P, C | pYR125 | This study, *cda6* is inserted in single copy |
| SG200_$P_{act}$cda6_ StrepII-tag | YR539 | *a1mfa2 bW2bE1 ipR[Pact_cda 6(w/o-GPI):StrepII-tag]ipS* | SG200 | P, C | pYR125 | This study, *cda6* is inserted in multiple copy |
| SG200_$P_{act}$cda7_ StrepII-tag | YR546 | *a1mfa2 bW2bE1 ipR[Pact_cda 7(w/o-GPI):StrepII-tag]ipS* | SG200 | P,C | pYR126 | This study, *cda7* is inserted in single copy |
| SG200_$P_{act}$cda7_ StrepII-tag | YR548 | *a1mfa2 bW2bE1 ipR[Pact_cda 7(w/o-GPI):StrepII-tag]ipS* | SG200 | P,C | pYR126 | This study, *cda7* is inserted in multiple copy |
| SG200_AM_PM | SR1326 | *a1 mfa2 bW2 bE1, ble; ipR[Pam1-GFP (AM)_Prsp3-mCherry (PM)]ipS* | SG200 | P,C | pSR422 | Krombach S. University, Marburg, Hesse, Germany. 2016 https://doi.org/10.17192/z2017.0051. AM and PM are inserted in single copy |
| SG200Δcda7_AM _PM | PH470 | *a1 mfa2 bW2 bE1, ble; cda7::hyg, ipR[Pam1-GFP (AM)_Prsp3-mCherry (PM)]ipS* | YR472 | P,H,C | pSR422 | This study, AM and PM are inserted in singel copy |

| | | | | | | |
|---|---|---|---|---|---|---|
| SG200 Δcda7,cda7_AM_ PM | PH476 | *a1 mfa2 bW2 bE1, ble; cda7::hyg, ipR[Pam1-GFP (AM)_Prsp3-mCherry (PM) / Pcda7_ cda7_Tcda7]i pS* | YR506 | P,H,C | pPH48 | This study, AM and PM are inserted in singel copy |
| Sporisorium scitamineum strain SscI8 | | | | | | Dutheil JY, Mannhaupt G, Schweizer G, C MKS, Munsterkotter M, Guldener U, Schirawski J, Kahmann R. Smut Fungi. Genome Biol Evol 8:681-704. 2016 |

[a] phleomycin (P), hygromycin (HY), carboxin (C).

**Table S1B** Plasmids used in the study

| **Name** | **Alternative name** | **Description** | **Reference/Construction** |
|---|---|---|---|
| p123 | | Contains the *gfp* gene under control of the *otef*-promoter and *nos* terminator as well as the *U. maydis*carboxin resistant *ip* allele ($ip^R$). p123 served as backbone to insert constructs into the *U. maydis ip* locus | Loubradou G, Brachmann A, Feldbrugge M, Kahmann R. Mol Microbiol 40:719-30. 2001 |
| pBS Hyg (-) | pMF1-h | Plasmid containing the hygromycin resistance cassette ($Hyg^R$) as SfiI-SfiI-fragment in pBlueScript | Brachmann A, Konig J, Julius C, Feldbrugge M. Mol Genet Genomics 272:216-26. 2004 |
| pJET1 stuffer | | Plasmid-derived from pJET1 (Fermentas, St. Leon-Rot). Contains a 0.6 kb stuffer sequence flanked by EcoRV sites disrupting the coding region of the eco47IR - Type-2 restriction enzyme | Kindly provided by K.O. Schink and M. Bölker |
| pET22b_StrepII_TrxA_ PaCDA_cd_ StrepII | | Plasmid used as a backbone for fusing genes with an N-terminal StrepII-TrxA tag and a C-terminal Strep-tag for heterologous expression in *E. coli* | Kindly provided by M. Bonin and B. M. Moerschbacher |

| | | | |
|---|---|---|---|
| pNEBUC_P$_{hsp70}$Cas9-tP$_{U6}$ | pMS73 | Self-replicating plasmid for *U. maydis* for Cas9 gene expression and for expression of several sgRNAs for CRISPR-Cas multiplexing | Schuster M, Schweizer G, Kahmann R. Fungal Genet Biol 112:21-30. 2018 |
| p123_P$_{crg}$Yps1 | pMM20 | Plasmid derived from p123 containing the Pcrg promoter | Kindly provided by M. Moretti |
| pP$_{tef}$Rab5aGn-CbxR | pUMa1712 | Plasmid containing the the geneticin resistance cassette, and designed for insertion in the *ip* locus | Kindly provided by M. Feldbrügge |
| pET22b_strepII-CSN-sfGFP-His6-E122Q | | Plasmid containing the probe CAP (chitosanase CSN with mutation in Glu122 replazed for Gln) tagged in N-terminal with StrepII tag and in C-terminal fused to sfGFP and His tag | Nampally M, Moerschbacher BM, Kolkenbrock S. Appl Environ Microbiol 78:3114-9. 2012 |
| pET22b_strepII-CSN-mKATE2-His6-E122Q | | Plasmid expressing CAP (chitosanase CSN with substitution of Glu122 by Gln) N-terminally tagged with StrepII tag and in C-terminally tagged with mKATE2 and His tag | Kindly provided by B. M. Moerschbacher |
| pET22b_strepII-sfGFP-His6-E122Q | pYR121 | Plasmid expressing sfGFP N-terminally tagged with StrepII tag and in C-terminally fused to a His tag | The sequence of *sfgfp* was amplified from pET22b_strepII-CSN-sfGFP-His6-E122Q with primers oYR330 and oYR254. The resulting the fragment was integrated via Gibson assembly into pET22b_strepII-sfGFP-His6-E122Q cut with NdeI/XhoI |
| pET22b_strepII-mKATE2-His6-E122Q | pYR99 | Plasmid expressing mKATE2 N-terminally tagged with a StrepII tag and C-terminally fused to a His tag | The sequence of *mkate2*was amplified from pET22b_strepII-CSN-mKATE2-His6-E122Q with primers oYR253 and oYR254.The resulting fragment was integrated via Gibson assembly into pET22b_strepII-sfGFP-His6-E122Q cut with NdeI/XhoI |
| p123_P$_{crg1}$cda2 (PAM mutated) | pYR100 | Plasmid-derived from pMM20 containing the sequence of *cda2* under the *crg1*-promotor and *nos* terminator. In *cda2* sequence the PAM sequence is modified prevent Cas9 cleavage | The region between the ATG and the Cas9 target sequence of *cda2*was amplified with primers oYR155/oYR157. The region between the Cas9 traget sequence and the stop codon was amplified with primers oYR158/oYR156. oYR157 and oYR158 introduce a modified PAM sequence to avoid cutting with Cas9. The fragments were integrated by Gibson assembly into pMM20 cut with BamHI and NotI |
| pJET_Δcda7 | pYR103 | pJET1-derived plasmid containing the construct for the deletion of *cda7*consisting of a hygromycin resistance cassette flanked by the left and right borders of the *cda7*gene | The left and right borders of *cda7*were PCR amplified from SG200 gDNA with primers oYR281/oYR282 and oYR283/oYR284. The hygromycin resistance cassette was obtained from SfiI digestion of pBS-Hyg (-). The three fragments were integrated via Gibson assembly into the EcoRV cut pJET1 backbone |

| | | | |
|---|---|---|---|
| p123_P$_{cda7}$cda7_T$_{cda7}$ | pYR122 | p123-derived plasmid containing the *cda7*gene under control of native promoter and terminator sequences | The *cda7* gene including promoter and terminator were PCR amplified from SG200 gDNA with primers oYR331/oYR332. The resulting PCR product was integrated via Gibson assembly into the backbone of p123 digested with HindIII/NotI |
| pET22b_Strep-Tag_TrxA_cda1_StrepII | pYR113 | pET22b_Strep_TrxA_PaCDA_cd_Strep-derived plasmid containing the dicodon optimized *cda1* gene without signal peptide and GPI anchor sequences for expression in *E. coli.*The N-terminus is fused to a StrepII-TrxA tag, the C-terminus carries a StrepII tag | The *cda1* gene dicodon optimized for expression in *E. coli* without the signal peptide and GPI anchor and carrying a 5′ linker and a 3′ StrepII tag respectively, was synthesized by IDT (Integrated DNA Technologies). This sequence was amplified with primers oYR315/oYR316. One fragment containing the sequence of a StrepII tag, Trx and a linker was amplified with primers oYR289/oYR290 form plasmid pET22b_StrepII_TrxA_PaCDA_cd_StrepII. The two fragments were integrated via Gibson assembly into the XbaI/NotI digested pET22b_StrepII_TrxA_PaCDA_cd_StrepII |
| pET22b_StrepII_TrxA_cda2_StrepII | pYR114 | pET22b_StrepII_TrxA_PaCDA_cd_StrepII-derived plasmid containing the dicodon optimized *cda2* gene without signal peptide and GPI anchor sequences for expression in *E. coli*. The N-terminus is fused to a StrepII-TrxA tag, the C-terminus carries a StrepII tag | The *cda2* gene dicodon optimized for expression in *E. coli*without the signal peptide and GPI anchor and carrying a 5′ linker and a 3′ StrepII tag respectively, was synthesized by IDT (Integrated DNA Technologies). This sequence was amplified with primers oYR315/oYR316. One fragment containing the sequence of a StrepII tag, Trx and a linker was amplified with primers oYR289/oYR290 form plasmid pET22b_StrepII_TrxA_PaCDA_cd_StrepII. The two fragments were integrated via Gibson assembly into the XbaI/NotI digested pET22b_StrepII_TrxA_PaCDA_cd_StrepII |
| pET22b_StrepII_TrxA_cda4_StrepII | pYR115 | pET22b_StrepII_TrxA_PaCDA_cd_StrepII-derived plasmid containing the dicodon optimized *cda4* gene without signal peptide sequence for expression in *E. coli*. The N-terminus is fused to a StrepII-TrxA tag, the C-terminus carries a StrepII tag | The *cda4* gene dicodon optimized for expression in *E. coli*without the signal peptide and carrying a 5′ linker and a 3′ StrepII tag respectively, was synthesized by IDT (Integrated DNA Technologies). This sequence was amplified with primers oYR315/oYR316. One fragment containing the sequence of a StrepII tag, Trx and a linker was amplified with primers oYR289/oYR290 form plasmid pET22b_StrepII_TrxA_PaCDA_cd_StrepII. The two fragments were integrated via Gibson assembly into the XbaI/NotI digested pET22b_StrepII_TrxA_PaCDA_cd_StrepII |
| pET22b_StrepII_TrxA_cda5_StrepII | pYR116 | pET22b_StrepII_TrxA_PaCDA_cd_StrepII-derived plasmid containing the dicodon optimized *cda5* gene without signal peptide and GPI anchor sequences for expression in *E. coli*. The N-terminus is fused to a StrepII-TrxA tag, the C-terminus carries a StrepII tag | The *cda5* gene dicodon optimized for expression in *E. coli*without the signal peptide and GPI anchor and carrying a 5′ linker and a 3′ StrepII tag respectively, was synthesized by IDT (Integrated DNA Technologies). This sequence was amplified with primers oYR315/oYR316. One fragment containing the sequence of Strep-tag, Trx and a linker was amplified with primers oYR289/oYR290 form plasmid pET22b_Strep_TrxA_PaCDA_cd_Strep. The two fragments were integrated via Gibson |

| | | | |
|---|---|---|---|
| | | | assembly into the XbaI/NotI digested pET22b_Strep_TrxA_PaCDA_cd_Strep. |
| pET22b_StrepII_TrxA_cda6_StrepII | pYR117 | pET22b_StrepII_TrxA_PaCDA_cd_StrepII-derived plasmid containing the dicodon optimized *cda6* gene without GPI anchor sequences for expression in *E. coli*. The N-terminus is fused to a StrepII-TrxA tag, the C-terminus carries a StrepII tag | The *cda6* gene dicodon optimized for expression in *E. coli*without the GPI anchor and carrying a 5´ linker and a 3´ StrepII tag respectively, was synthesized by IDT (Integrated DNA Technologies). This sequence was amplified with primers oYR315/oYR316. One fragment containing the sequence of a StrepII tag, Trx and a linker was amplified with primers oYR289/oYR290 form plasmid pET22b_StrepII_TrxA_PaCDA_cd_StrepII. The two fragments were integrated via Gibson assembly into the XbaI/NotI digested pET22b_StrepII_TrxA_PaCDA_cd_StrepII |
| pET22b_StrepII_TrxA_cda3_StrepII | pYR118 | pET22b_StrepII_TrxA_PaCDA_cd_StrepII-derived plasmid containing the dicodon optimized *cda3* gene without signal peptide and GPI anchor sequences for expression in *E. coli*. The N-terminus is fused to a StrepII-TrxA tag, the C-terminus carries a StrepII tag | The *cda3*gene dicodon optimized for expression in *E. coli*without the signal peptide and GPI anchor and carrying a 5´ linker and a 3´ StrepII tag respectively, was synthesized by IDT (Integrated DNA Technologies). This sequence was amplified with primers oYR315/oYR316. One fragment containing the sequence of a StrepII tag, Trx and a linker was amplified with primers oYR289/oYR290 form plasmid pET22b_StrepII_TrxA_PaCDA_cd_StrepII. The two fragments were integrated via Gibson assembly into the XbaI/NotI digested pET22b_StrepII_TrxA_PaCDA_cd_StrepII |
| pET22b_StrepII_TrxA_cda7_StrepII | pYR119 | pET22b_StrepII_TrxA_PaCDA_cd_StrepII-derived plasmid containing the dicodon optimized *cda7* gene without signal peptide and GPI anchor sequences for expression in *E. coli*. The N-terminus is fused to a StrepII-TrxA tag, the C-terminus carries a StrepII tag | The *cda7* gene dicodon optimized for expression in *E. coli*without the signal peptide and GPI anchor and carrying a 5´ linker and a 3´ StrepII tag respectively, was synthesized by IDT (Integrated DNA Technologies). This sequence was amplified with primers oYR315/oYR316. One fragment containing the sequence of a StrepII tag, Trx and a linker was amplified with primers oYR289/oYR290 form plasmid pET22b_StrepII_TrxA_PaCDA_cd_StrepII. The two fragments were integrated via Gibson assembly into the XbaI/NotI digested pET22b_StrepII_TrxA_PaCDA_cd_StrepII |

| | | | |
|---|---|---|---|
| p123_P$_{am1}$GFP_P$_{rsp3}$mCherry-HA | pSR422 | p123-derived plasmid containing the *gfp* gene under the control of *am1*(*umag_01779)*promoter, that is specifically expressed during appressorium formation and a *mcherry* gene under the control of the native *rsp3*(*umag_03274*) promoter that is induced upon penetration. This plasmid allows the visualization of appresoria formation and successful penetration events | Krombach S. University, Marburg, Hesse, Germany. 2016 ttps://doi.org/10.17192/z2017.0051. |
| p123_P$_{actin}$mCherry | pDL252 | p123-derived plasmid containing the *mcherry* gene under under the control of the *actin* promoter. | Lanver D, Muller AN, Happel P, Schweizer G, Haas FB, Franitza M, Pellegrin C, Reissmann S, Altmuller J, Rensing SA, Kahmann R. Plant Cell 30:300-323. 2018 |
| p123_P$_{actin}$cda3-StrepII_T$_{nos}$ | pYR124 | p123-derived plasmid containing the *cda3*gene under control of the *actin* promoter and the *nos* terminator. The sequence encoding the GPI anchor is replaced by a StrepII tag | The gene sequence of *cda3* without GPI anchor sequence was PCR amplified from SG200 gDNA with primers oYR341/oYR342, the reverse primer (oYR342) includes the sequence of StrepII tag. The resulting PCR product was integrated via Gibson assembly into the p123-P$_{actin}$mcherry digested with NcoI/AscI. |
| p123_P$_{actin}$cda6-StrepII_T$_{nos}$ | pYR125 | p123-derived plasmid containing the *cda6*gene under control of the *actin* promoter and the *nos* terminator. The sequence encoding the GPI anchor is replaced by a StrepII tag | The gene sequence of *cda5* from the ATG (predicted in NCBI, ID: 23565582) to the 3´end without GPI anchor sequence was PCR amplified from SG200 gDNA with primers oYR343/oYR344, the reverse primer (oYR344) includes the sequence of StrepII tag. The resulting PCR product was integrated via Gibson assembly into the p123-P$_{actin}$mcherry digested with NcoI/AscI |
| p123_P$_{actin}$cda7-StrepII_T$_{nos}$ | pYR126 | p123-derived plasmid containing the *cda7*gene under control of the *actin* promoter and the *nos* terminator. The sequence encoding the GPI anchor is replaced by a StrepII tag | The gene sequence of *cda3* without GPI anchor sequence was PCR amplified from SG200 gDNA with primers oYR345/oYR346 the reverse primer (oYR346) includes the sequence of StrepII tag. The resulting PCR product was integrated via Gibson assembly into the p123-P$_{actin}$mcherry digested with NcoI/AscI |
| pNEBUC_P$_{hsp70}$Cas9_cda3_cda2_cda4 | pSL3 | pMS73_derived plasmid encoding sgRNAs under the control of *U6*, *Leu TAA tRNA* and *Gly Leu TAA tRNA* promoters, respectively for targeting *cda2*, *cda3* and *cda4* | Fragments encoding the sgRNAs for targeting *cda2*, *cda3* and *cda4* including the promoters and scaffolds were obtained from Eurofins Genomics (Ebersberg, Germany) as gBLOCKs gSL1, gSL2 and gSL7. The three gBLOCK fragments were integrated into the Acc65I linearized pMS73 backbone via Gibson assembly |

| | | | |
|---|---|---|---|
| pNEBUC_P$_{hsp70}$Cas9_cda5_cda6_cda1 | pSL5 | pMS73_derived plasmid encoding sgRNAs under the control of *U6*, *Leu TAA tRNA* and *Trp CAA tRNA* promoters, respectively for targeting *cda5*, *cda6* and *cda1* | Fragments encoding the sgRNAs for targeting *cda5*, *cda6* and *cda1* including the promoters, scaffolds and terminators, were obtained from as gBLOCKs, gSL6 (obtained from Eurofins Genomics, Ebersberg, Germany), gSL10 (PCR product with primers oSL35/oSL36 on gSL1 template) and gSL11 (PCR product with primers oSL37/oSL1 on gSL5 template). The three gBLOCK fragments were integrated into the Acc65I linearized pMS73 backbone via Gibson assembly |
| pNEBUC_P$_{hsp70}$Cas9_cda2 | pSL6 | pMS73_derived plasmid encoding sgRNAs under the control of U6 promoter for targeting *cda2* | Fragment encoding the sgRNAs for targeting *cda2* including the scaffold were obtained as the gBLOCK, gSL12 (PCR product with primers oSL54/oPH138 on gSL6 template). The gBLOCK fragment was integrated into the Acc65I linearized pMS73 backbone via Gibson assembly |
| pNEBUC_P$_{hsp70}$Cas9_cda1 | pPH38 | pMS73_derived plasmid encoding sgRNAs under the control of *U6* promoter for targeting *cda1* | Fragment encoding the sgRNAs for targeting *cda1* including the scaffold were obtained as the gBLOCK, fPH4 (PCR product with primers oPH307/oPH138 on fDL17 template). The gBLOCK fragment was integrated into the Acc65I linearized pMS73 backbone via Gibson assembly |
| pNEBUC_P$_{hsp70}$Cas9_cda6_cda5 | pPH39 | pMS73_derived plasmid encoding sgRNAs under the control of *U6* and *Leu TAA tRNA* promoters, respectively for targeting *cda6* and *cda5* | Fragments encoding the sgRNAs for targeting *cda6* and *cda5* including the promoters and scaffolds were obtained as gBLOCK fPH5 (PCR product with primers oPH308/oPH309 on fDL15 template) and fPH6 PCR product with primers oPH310/oPH311 on fDL16 template). The two gBLOCK fragments were integrated into the Acc65I linearized pMS73 backbone via Gibson assembly |
| pNEBUC_P$_{hsp70}$Cas9_cda4 | pPH40 | pMS73_derived plasmid encoding sgRNAs under the control of *U6* promoter for targeting *cda4* | Fragment encoding the sgRNAs for targeting *cda4* including the scaffold were obtained as the gBLOCK, fPH7 (PCR product with primers oPH312/oPH138 on fDL17 template). The gBLOCK fragment was integrated into the Acc65I linearized pMS73 backbone via Gibson assembly |
| pNEBUC_P$_{hsp70}$Cas9_cda6 | pPH41 | pMS73_derived plasmid encoding sgRNAs under the control of *U6* promoter for targeting *cda6* | Fragment encoding the sgRNAs for targeting *cda6* including the scaffold were obtained as the gBLOCK, fPH8 (PCR product with primers oPH313/oPH138 on gBLOCK fDL17 template). The gBLOCK fragment was integrated into the Acc65I linearized pMS73 backbone via Gibson assembly |
| pNEBUC_P$_{hsp70}$Cas9_cda5 | pPH42 | pMS73_derived plasmid encoding sgRNAs under the control of *U6* promoter for targeting *cda5* | Fragment encoding the sgRNAs for targeting *cda5* including the scaffold were obtained as the gBLOCK, fPH9 (PCR product with primers oPH314/oPH138 fDL17 template). The gBLOCK fragment was integrated into the Acc65I linearized pMS73 backbone via Gibson assembly |

| | | | |
|---|---|---|---|
| pNEBUC_P$_{hsp70}$Cas9_cda1_cda2 | pPH43 | pMS73_derived plasmid encoding sgRNAs under the control of *U6* and *Leu TAA tRNA* promoters, respectively for targeting *cda1* and *cda2* | Fragments encoding the sgRNAs for targeting *cda1* and *cda2* including the promoters and scaffolds were obtained as gBLOCK fPH10 (PCR product with using primers oPH315/oPH316 on fDL15 template) and fPH11 (PCR product with primers oPH317 and oPH311on fDL16 template. The two gBLOCK fragments were integrated into the Acc65I linearized pMS73 backbone via Gibson assembly |
| pJET1_P$_{crg1}$cda2 (PAM mutated). | pPH45 | pJET1 stuffer derived plasmid for replacing the promoter of *cda2* in locus with P$_{crg1}$*cda2*. In the construct 1.5 kb upstream of *cda2*are deleted and replaced by 1.4 kb *crg1* promoter fused to the *cda2 ORF* containing a mutated version of the PAM sequence. From this plasmid a 3.7 kb SspI fragment was excised and used as donor DNA in co-transformation with pSL6 | The region upstream of the *cda2*promoter was PCR amplified with primers oPH326/oPH327 (908 bp) on genomic DNA of SG200. The *crg1* promoter fused to the *cda2* gene containing the mutated PAM sequence was PCR amplified with primers oPH328/oPH329 (2897 bp) on pYR100 template. Both fragments were integrated into the EcoRV backbone of pJET1stuffer with Gibson assembly. |
| pUMa1712_P$_{am}$GFP_P$_{rsp3}$mCherry-HA /Geneticin resistance | pPH48 | pUMa1712 derived plasmid encoding the AM1 appressorial marker gene as well as the PM P$_{rsp3}$*mCherry* penetration marker from pRS422 | The PM and AM markers were PCR amplified with primers oPH355 and oPH356 from pSR422 and cloned with Gibson assembly into the EcoRI and HindIII sites of pUMA1712 |
| p123_P$_{cda6}$cda6_T$_{cda6}$ | pPH49 | p123-derived plasmid encoding *cda6* with native promoter (2293bp) and terminator (960bp) used for complementation | The *cda6* gene including promoter and terminator was PCR amplified with primers oPH358 and oPH359 from SG200 gDNA and cloned with Gibson Assembly into the Acc65I and EcoRV backbone of p123 |
| p123_P$_{cda2}$sscda6_T$_{nos}$ | pPH50 | p123-derived plasmid encoding the ortholog of *cda6* from *Sporisorium scitamineum* (S*sc30930.1)* under the control of the *U. maydiscda2* (2293bp) promotor and *nos* terminator | The *cda2* promoter was PCR amplified with primers oPH362/oPH363 on gDNA of SG200. The ORF of *ssc30930.1*was PCR amplified with primer oPH364/oYR386 on gDNA of *Sporisorium scitamineum* strain *SscI8.*Both fragments were cloned into Acc65I and NotI linearized p123 by Gibson assembly |

**Table S1C** Oligonucleotides used in the study

| Name | Sequence | Comment |
|---|---|---|
| oSR135_RT-qPCR/qPCR umppi | AAAGAACACCGGACTTGG | Brefort T, Tanaka S, Neidig N, Doehlemann G, Vincon V, Kahmann R. PLoS Pathog 10:e1003866. 2014 |

| | | |
|---|---|---|
| oSR136_RT-qPCR/qPCR umppi | ACATCGTCAAGGCTATCG | Brefort T, Tanaka S, Neidig N, Doehlemann G, Vincon V, Kahmann R. PLoS Pathog 10:e1003866. 2014 |
| oSR137_RT-qPCR/qPCR_Z.mays_gapdh | CTTCGGCATTGTTGAGGGTTTG | Brefort T, Tanaka S, Neidig N, Doehlemann G, Vincon V, Kahmann R. PLoS Pathog 10:e1003866. 2014 |
| oSR138_RT-qPCR/qPCR_Z.mays_gapdh | TCCTTGGCTGAGGGTCCGTC | Brefort T, Tanaka S, Neidig N, Doehlemann G, Vincon V, Kahmann R. PLoS Pathog 10:e1003866. 2014 |
| oYR261_cda2_qPCR_Fw | CAGAACGCGTGGTTCATC | This study |
| oYR262_cda2_qPCR_Rv | GGAATTCGTGGTGCTAGAAG | This study |
| oYR263_cda1_qPCR_Fw | GATCTTTGGCCTCACCAATG | This study |
| oYR264_cda1_qPCR_Rv | ACAGAAGCGTAGTCCTGTC | This study |
| oYR265_cda4_qPCR_Fw | AGCGGTCAACATTGTTCG | This study |
| oYR266_cda4_qPCR_Rv | GAATCGCGGTTCTGGTATC | This study |
| oYR267_cda5_qPCR_Fw | CAGGTGTGAACCGAAATCTG | This study |
| oYR268_cda5_qPCR_Rv | CACTGCTCCAATACCTCAAC | This study |
| oYR269_cda6_qPCR_Fw | GCGGTTCCTTTGCTACTG | This study |
| oYR270_cda6_qPCR_Rv | TCTGCTGCTTGCTGATTG | This study |
| oYR271_cda3_qPCR_Fw | TTGCCGAGCTATACTACACC | This study |
| oYR272_cda3_qPCR_Rv | TAGCAATCGCACGAACAC | This study |
| oYR337_cda7_qPCR_Fw | CCGCCTCAGAAGAACAATG | This study |
| oYR338 _cda7_qPCR_Rv | AGTCGTCAAACGCACAAG | This study |
| oYR281_LB_cda7_Fw | GGCTCGAGTTTTTCAGCAAGATAATATTCATGAGCCCTTGAGACAAGC | This study |
| oYR282_LB_cda7_Rv | ATTGTCACGCCATGGTGGCCATCTAGGCCCGTGATGGTTCACGCCAAAC | This study |
| oYR283_RB_cda7_Fw | TGCGGCCGCATTAATAGGCCTGAGTGGCCATATGCTACGTCGTATCTCG | This study |
| oYR284_RB_cda7_Rv | AGGAGATCTTCTAGAAAGATAATATTAAATGGCGCTGCTGCATGGG | This study |
| oYR289_Strep_TrxA_linker_Fw | GTGAGCGGATAACAATTCCC | This study |
| oYR290_Strep_TrxA_linker_Rv | CTTGTCATCGTCATCACCAG | This study |

| | | |
|---|---|---|
| oYR331_Pcda7_cda7_Tcda7_Fw | ACTATAGAACTCGAGCAGCTGAAGCTTATGGCTG CAATCGCATCTCC | This study |
| oYR332_Pcda7_cda7_Tcda7_Rv | TGAACGATCTGCAGCCGGGCGGCCGCAGTTTAGT AATAGTCACAGAG | This study |
| oYR315_Linker_cdacodonop_FW | GGTTCTGGTTCTGGTGATGACG | This study |
| oYR316_cdacodonop_Strep-Tag_Rv | GGTGGTGCTCGAGTGCGGCCGCAAGCTCATTTTC AAACTGCGGATG | This study |
| oYR341_Pact_cda3_Tnos_Fw | CAAAACCTAGTTCACAGTCATCCATGAAGCTTTCC TCGACAGCC | This study |
| oYR342_Pact_cda3_Tnos_Rv | ATCGCCGGGCGGCCGGCGCGCCTCATTTTTCAAA CTGCGGATGTGACCACGACTTATCTGCGGACTGA TTC | This study |
| oYR343_Pact_cda6_Tnos_Fw | CAAAACCTAGTTCACAGTCATCCATGCTAGTGAG TAAAGGTAAG | This study |
| oYR344_Pact_cda6_Tnos_RV | GATCGCCGGGCGGCCGGCGCGCCTCATTTTTCAA ACTGCGGATGTGACCAACTAGACTTGTGGCTCTG CTG | This study |
| oYR345_Pact_cda7_Tnos_Fw | CAAAACCTAGTTCACAGTCATCCATGAAGTCTAC CACGGTATTC | This study |
| oYR346_Pact_cda7_Tnos_RV | GATCGCCGGGCGGCCGGCGCGCCTCATTTTTCAA ACTGCGGATGTGACCAGCCGAACGTCCTGTAGGG GTC | This study |
| oYR253_FW_pET22b_mKate2 | TTAAGAAGGAGATATACATATGTGGTCACATCCT CAATTTGAAAAAATGGTTAGCGAGCTGATC | This study |
| oYR254_Rv_pET22b_mKate2 | AGTGGTGGTGGTGGTGGTGC | This study |
| oYR330_FW_pET22b_sfGFP | TTAAGAAGGAGATATACATATGTGGTCACATCCT CAATTTGAAAAAATGCGTAAAGGCGAAGAGCTG | This study |
| oYR255_p123_Pcrg_cda2_FW | CGAGGTTTGCGGTGAAACTCGGGATCCATGCGTC TCTCCGTCTCC | This study |
| oYR256_p123_Pcrg_cda2_RV | ACGATCTGCAGCCGGGCGGCCGCTCAGAGCAAGA GAGCAAAAGCG | This study |
| oYR257_p123_Pcrg_cda2_RV_PAMmodf | GCACGCTTGTGAAACAGGTGAC | This study |
| oYR258_p123_Pcrg_cda2_FW_PAMmodf | GTCACCTGTTTCACAAGCGTGC | This study |
| oSL11 | CAAAATTCCATTCTACAACG | This study |
| oSL14 | TCCCTACCGCAGATCAAACACTGACC | This study |
| oSL17 | GACGTTGTCGTGAATCGATTCTTGACCAGACGGG | This study |
| oSL54_cda2_f | CAAAATTCCATTCTACAACGGGTGCTCAGAGGCA CGCTTGGTTTTAGAGCTAGAAATAGC | This study |
| oSL35_gSL10-f | CAAAATTCCATTCTACAACGGAATGGAGGAAACG GAAGCGGTTTTAGAGCTAGAAATAGC | This study |
| oSL36_gSL10-r | GAACTAGCCGACATCTTACCTTGACCAGACGGGA TTCGAAC | This study |
| oSL37_gSL11-f | GGTAAGATGTCGGCTAGTTCGTTTTAGAGCTAGA AATAGC | This study |
| oPH138_Phsp70-r | GGCGTTCGACTCTTGGCAG | Lanver D, Muller AN, Happel P, Schweizer G, Haas FB, Franitza M, Pellegrin C, Reissmann S, |

| | | |
|---|---|---|
| | | Altmuller J, Rensing SA, Kahmann R. Plant Cell 30:300-323. 2018 |
| oPH307_cda1_Pu6-f | TTCAAAATTCCATTCTACAACGTGTTTGATCTGCG GTAGGGAGTTTTAGAGCTAGAAATAG | This study |
| oPH308_cda6-3-f | TTCAAAATTCCATTCTACAACGGCTGCTGCTAGGG ACGGTGGTTTTAGAGCTAGAAATAG | This study |
| oPH309_cda6-3-r | CGAGAAGCTGATCTAGAGCTTGACCAGACGGGAT TCG | This study |
| oPH310_cda5-3-f | CGAATCCCGTCTGGTCAAGCTCTAGATCAGCTTCT CGGTTTTAGAGCTAGAAATAG | This study |
| oPH311_cda5-3-r | GGCGTTCGACTCTTGGCAGGTACCAAAAAAAGCA CCGACTCGG | This study |
| oPH312_cda4_Pu6-f | TTCAAAATTCCATTCTACAACGGCGTGCATAAAG CGTGTAGTGTTTTAGAGCTAGAAATAG | This study |
| oPH313_cda6_Pu6-f | TTCAAAATTCCATTCTACAACGGCTGCTGCTAGGG ACGGTGGTTTTAGAGCTAGAAATAG | This study |
| oPH314_cda5_Pu6-f | TTCAAAATTCCATTCTACAACGCTCTAGATCAGCT TCTCGGTTTTAGAGCTAGAAATAG | This study |
| oPH315_cda1_f | TTCAAAATTCCATTCTACAACGTGTTTGATCTGCG GTAGGGAGTTTTAGAGCTAGAAATAG | This study |
| oPH316_cda1_r | CAAGCGTGCCTCTGAGCACCTTGACCAGACGGGA TTCG | This study |
| oPH317_cda2_f | CGAATCCCGTCTGGTCAAGGTGCTCAGAGGCACG CTTGGTTTTAGAGCTAGAAATAG | This study |
| oPH326_uf_cda2-f | GATGGCTCGAGTTTTCAGCAAGATAATATTGATA TGCGGGCGGGAGTC | This study |
| oPH327_uf_cda2-r | CTCTCAAACTCTTGGCCAGACCGAGCGCTACGAA AC | This study |
| oPH328_Pcrg-cda2-f | GTTTCGTAGCGCTCGGTCTGGCCAAGAGTTTGAG AG | This study |
| oPH329_orf-cda2-r | GTAGGAGATCTTCTAGAAAGATAATATTCAGAGC AAGAGAGCAAAAGC | This study |
| oPH355_BBp123-f | GACACTATAGAACTCGAGCAGC | This study |
| oPH356_BBp123-r | CTTTAATTTGCAAGCTGTAGGAGTGCGCTAGCGG GAGACCGGCAGATCTG | This study |
| oPH358_P-f-cda6 | GCCTGCAGGTCGAAATTCGAGCTCGGTACCTCTTG CTAAGCTATTATATCAAGG | This study |
| oPH359_T-r-cda6 | CTATAGGGAGACCGGCAGATCTGATATCGGTGGC ATCGCTGTAAG | This study |
| oPH362_P-f-cda2 | GCCTGCAGGTCGAAATTCGAGCTCGGTACCGATA TGCGGGCGGGAGTC | This study |
| oPH363_P-r-cda2 | GTAGAATTGTGGTCGCCTTCAACATGATATGAAA AGTCGATGAGGGG | This study |
| oPH364_orf-f-sscda6 | TCGCCCCTCATCGACTTTTCATATCATGTTGAAGG CGACCA | This study |
| oYR386_orf-r-sscda6 | GTTTGAACGATCTGCAGCCGGGCGGCCGCGGGAT TAGACGAAGACCAAG | This study |

**Table S1D** Double stranded DNA fragments used in this study

| Fragment ID | Type | Architecture (5' - 3')[a] | Sequence (5' - 3')[a] | Plasmid | Comment |
|---|---|---|---|---|---|
| gSL1 | gBLOCK (synthesized) | assembly overhang (partial u6 promoter) - *cda3* target sequence - scaffold and terminator - Leu TAA tRNA promoter (UMAG_16041) - assembly overhang (*cda2* target sequence) | CAAAATTCCATTCTACAACGGCTTTCCTCGACAGCCATCGGTTTTAGAGCTAGAAATAGCAAGTTAAAATAAGGCTAGTCCGTTATCAACTTGAAAAAGTGGCACCGAGTCGGTGCTTTTTTTTCGATGTGTGCACAAATCATCGTGCTAGACGCCAAGCGAAGCGGAAAGTTTGAGACCCGATTATAATCGGTATTGAGCAATTCTGTCTTCCAAACTTCATGACCAGTAGGCAAGTGGTTAAGCCGCGAGCTTTAAGCCCGATGTAGGCCCGATCTCAGCCCTTCGTTAACTCTCGTTTGTCGCGGGTTCGAATCCCGTCTGGTCAAGGGTGCTCAGAGGCACGCTTG | pSL3 | This study |
| gSL2 | gBLOCK (synthesized) | *cda2* target sequence - scaffold and terminator - Gly GCC tRNA promoter (UMAG_16051) - assembly overhang (*cda4* target sequence) | GGGTGCTCAGAGGCACGCTTGGTTTTAGAGCTAGAAATAGCAAGTTAAAATAAGGCTAGTCCGTTATCAACTTGAAAAAGTGGCACCGAGTCGGTGCTTTTTTACCTCAGACCAAGCGTGAACAGGCGATGCTTGTGACATAGAAATGGAACGGTCATAATATTACATATAATTACAGTGTAACAAGATATATGCACCTGAAAGCATTGGTAGTGTAGTGGTATCACGGGACGTTGCCAGCCCGCCTTATTCAAGGCTTAACCACGAATTCGTCCCGACCGGGGTTCGATTCCCCGTCGATGCAGGCGTGCATAAAGCGTGTAGT | pSL3 | This study |
| gSL7 | gBLOCK (synthesized) | *cda4* target sequence - scaffold and terminator - assembly overhang (partial hsp70 promoter) | GGCGTGCATAAAGCGTGTAGTGTTTTAGAGCTAGAAATAGCAAGTTAAAATAAGGCTAGTCCGTTATCAACTTGAAAAAGTGGCACCGAGTCGGTGCTTTTTTGTACCTGCCAAGAGTCGAACGCC | pSL3 | This study |

| | | | | | |
|---|---|---|---|---|---|
| gSL10 | PCR product amplified with primers oSL35/oSL36 on gSL9 template | assembly overhang (partial u6 promoter) - *cda5* target sequence - scaffold and terminator - Leu TAA tRNA promoter (UMAG_16041) - assembly overhang (*cda6* target sequence#2) | CAAAATTCCATTCTACAACGGAATGGAGGAAACGGAAGCGGTTTTAGAGCTAGAAATAGCAAGTTAAAATAAGGCTAGTCCGTTATCAACTTGAAAAAGTGGCACCGAGTCGGTGCTTTTTTTTCGATGTGTGCACAAATCATCGTGCTAGACGCCAAGCGAAGCGGAAAGTTTTGAGACCCGATTATAATCGGTATTGAGCAATTCTGTCTTCCAAACTTCATGACCAGTAGGCAAGTGGTTAAGCCGCGAGCTTTAAGCCCGATGTAGGCCCGATCTCAGCCCTTCGTTAACTCTCGTTTGTCGCGGGTTCGAATCCCGTCTGGTCAAGGTAAGATGTCGGCTAGTTC | pSL5 | This study |
| gSL11 | PCR product amplified with primers oSL37/oSL14 on gSL5 template | *cda6* target sequence#2 - scaffold and terminator - tRNA Trp CCA promoter - assembly overhang (*cda1* target sequence) | GGTAAGATGTCGGCTAGTTCGTTTTAGAGCTAGAAATAGCAAGTTAAAATAAGGCTAGTCCGTTATCAACTTGAAAAAGTGGCACCGAGTCGGTGCTTTTTTTCCTGCTTCCATACTGCAAGCTGTCGTTAGGTAAAGCTGCCGATGTTCAAACCTACAATTCTTCCGTATATGTATACACTTTATATTACTTAATCATAACGCGGCCTCGTAGCTCAGGGGTAGAGCGTCGGCTTCCAGCCGTACATAGGAACATTCCGAAGGCCATCCGTTCAAATCGGGTCGGGGTCAGTGTTTGATCTGCGGTAGGGA | pSL5 | This study |
| gSL6 | gBLOCK (synthesized) | *cda1* target sequence - scaffold and terminator - assembly overhang (partial hsp70 promoter) | GTGTTTGATCTGCGGTAGGGAGTTTTAGAGCTAGAAATAGCAAGTTAAAATAAGGCTAGTCCGTTATCAACTTGAAAAAGTGGCACCGAGTCGGTGCTTTTTTTGTACCTGCCAAGAGTCGAACGCC | pSL5 | This study |

| | | | | | |
|---|---|---|---|---|---|
| gSL5 | gBLOCK (synthesized) | cda6 target sequence#1 - scaffold and terminator - tRNA TrpCAA promoter - assembly overhang (cda1 target sequence) | GAATCGATTCACGACA ACGTCGTTTTAGAGCTA GAAATAGCAAGTTAAA ATAAGGCTAGTCCGTTA TCAACTTGAAAAAGTG GCACCGAGTCGGTGCTT TTTTTCCTGCTTCCATAC TGCAAGCTGTCGTTAGG TAAAGCTGCCGATGTTC AAACCTACAATTCTTCC GTATATGTATACACTTT ATATTACTTAATCATAA CGCGGCCTCGTAGCTCA GGGGTAGAGCGTCGGC TTCCAGCCGTACATAGG AACATTCCGAAGGCCAT CCGTTCAAATCGGGTCG GGGTCAGTGTTTGATCT GCGGTAGGGA | gBLOCK used as PCR template | This study |
| gSL12 | PCR product amplified with primers oSL54/oPH138 on gSL6 template | assembly overhang (partial u6 promoter) - *cda2* target sequence - scaffold and terminator - assembly overhang (partial hsp70 promoter) | CAAAATTCCATTCTACA ACGGGTGCTCAGAGGC ACGCTTGGTTTTAGAGC TAGAAATAGCAAGTTA AAATAAGGCTAGTCCGT TATCAACTTGAAAAAGT GGCACCGAGTCGGTGCT TTTTTTGTACCTGCCAA GAGTCGAACGCC | pSL6 | This study |
| fDL15 | gBLOCK (synthesized) | assembly overhang (partial u6 promoter) - UMAG_02625 target sequence#1 - scaffold and terminator - Leu TAA tRNA promoter (UMAG_16041) - assembly overhang (UMAG_06253 target sequence#1) | TTCAAAATTCCATTCTA CAACGGTGCACACCTAT CTCGAGCGTTTTAGAGC TAGAAATAGCAAGTTA AAATAAGGCTAGTCCGT TATCAACTTGAAAAAGT GGCACCGAGTCGGTGCT TTTTTTTCGATGTGTGC ACAAATCATCGTGCTAG ACGCCAAGCGAAGCGG AAAGTTTTGAGACCCGA TTATAATCGGTATTGAG CAATTCTGTCTTCCAAA CTTCATGACCAGTAGGC AAGTGGTTAAGCCGCG AGCTTTAAGCCCGATGT AGGCCCGATCTCAGCCC TTCGTTAACTCTCGTTT GTCGCGGGTTCGAATCC CGTCTGGTCAACAATGC TGAAACCGTAGCAG | | Lanver D, Muller AN, Happel P, Schweizer G, Haas FB, Franitza M, Pellegrin C, Reissmann S, Altmuller J, Rensing SA, Kahmann R. Plant Cell 30:300-323. 2018 |

| | | | | | |
|---|---|---|---|---|---|
| fDL16 | gBLOCK (synthesized) | UMAG_06253 target sequence#1 - scaffold and terminator - Gly GCC tRNA promoter (UMAG_16051) - assembly overhang (UMAG_04577 target sequence) | CAATGCTGAAACCGTAGCAGGTTTTAGAGCTAGAAATAGCAAGTTAAAATAAGGCTAGTCCGTTATCAACTTGAAAAAGTGGCACCGAGTCGGTGCTTTTTTTACCTCAGACCAAGCGTGAACAGGCGATGCTTGTGACATAGAAATGGAACGGTCATAATATTACATATAATTACAGTGTAACAAGATATATGCACCTGAAAGCATTGGTAGTGTAGTGGTATCACGGGACGTTGCCAGCCCGCCTTATTCAAGGCTTAACCACGAATTCGTCCCGACCGGGGTTCGATTCCCCGTCGATGCAGATAATGATCAAAAGTCCCA | | Lanver D, Muller AN, Happel P, Schweizer G, Haas FB, Franitza M, Pellegrin C, Reissmann S, Altmuller J, Rensing SA, Kahmann R. Plant Cell 30:300-323. 2018 |
| fDL17 | gBLOCK (synthesized) | UMAG_04577 target sequence - scaffold and terminator - assembly overhang (partial hsp70 promoter) | GATAATGATCAAAAGTCCCAGTTTTAGAGCTAGAAATAGCAAGTTAAAATAAGGCTAGTCCGTTATCAACTTGAAAAAGTGGCACCGAGTCGGTGCTTTTTTTGGTACCTGCCAAGAGTCGAACGCC | | Lanver D, Muller AN, Happel P, Schweizer G, Haas FB, Franitza M, Pellegrin C, Reissmann S, Altmuller J, Rensing SA, Kahmann R. Plant Cell 30:300-323. 2018 |
| fPH4 | PCR product amplified with primers oPH307/oPH138 on fDL17 template | assembly overhang (partial u6 promoter) - *cda1* target sequence - scaffold and terminator - assembly overhang (partial hsp70 promoter) | TTCAAAATTCCATTCTACAACGTGTTTGATCTGCGGTAGGGAGTTTTAGAGCTAGAAATAGCAAGTTAAAATAAGGCTAGTCCGTTATCAACTTGAAAAAGTGGCACCGAGTCGGTGCTTTTTTTGGTACCTGCCAAGAGTCGAACGCC | pPH38 | This study |

| | | | | | |
|---|---|---|---|---|---|
| fPH5 | PCR product amplified with primers oPH308/oPH309 on fDL15 template | assembly overhang (partial u6 promoter) - *cda6* target sequence#3 - scaffold and terminator -Leu TAA tRNA promoter (UMAG_16041) - *assembly overhang (cda5 target sequence#3)* | TTCAAAATTCCATTCTACAACGGCTGCTGCTAGGGACGGTGGTTTTAGAGCTAGAAATAGCAAGTTAAAATAAGGCTAGTCCGTTATCAACTTGAAAAAGTGGCACCGAGTCGGTGCTTTTTTTTCGATGTGTGCACAAATCATCGTGCTAGACGCCAAGGAACGGAAAGTTTTGAGACCCGATTATAATCGGTATTGAGCAATTCTGTCTTCCAAACTTCATGACCAGTAGGCAAGTGGTTAAGCCGCGAGCTTTAAGCCCGATGTAGGCCCGATCTCAGCCCTTCGTTAACTCTCGTTTGTCGCGGGTTCGAATCCCGTCTGGTCAAGCTCTAGATCAGCTTCTCG | pPH39 | This study |
| fPH6 | PCR product amplified with primers oPH310/oPH311 on fDL16 template | assembly overhang (partial Leu TAA tRNA promoter (UMAG_16041) -*cda5* target sequence#3 - scaffold and terminator -assembly overhang (partial hsp70 promoter) | CGAATCCCGTCTGGTCAAGCTCTAGATCAGCTTCTCGGTTTTAGAGCTAGAAATAGCAAGTTAAAATAAGGCTAGTCCGTTATCAACTTGAAAAAGTGGCACCGAGTCGGTGCTTTTTTTGGTACCTGCCAAGAGTCGAACGCC | pPH39 | This study |
| fPH7 | PCR product amplified with primers oPH312/oPH138 on fDL17 template | assembly overhang (partial u6 promoter) - *cda4* target sequence - scaffold and terminator - assembly overhang (partial hsp70 promoter) | TTCAAAATTCCATTCTACAACGGCGTGCATAAAGCGTGTAGTGTTTTAGAGCTAGAAATAGCAAGTTAAAATAAGGCTAGTCCGTTATCAACTTGAAAAAGTGGCACCGAGTCGGTGCTTTTTTTGGTACCTGCCAAGAGTCGAACGCC | pPH40 | This study |
| fPH8 | PCR product amplified with primers oPH313/oPH138 on fDL17 template | assembly overhang (partial u6 promoter) - *cda6*target sequence - scaffold and terminator - assembly overhang (partial hsp70 promoter) | TTCAAAATTCCATTCTACAACGGCTGCTGCTAGGGACGGTGGTTTTAGAGCTAGAAATAGCAAGTTAAAATAAGGCTAGTCCGTTATCAACTTGAAAAAGTGGCACCGAGTCGGTGCTTTTTTGGTACCTGCCAAGAGTCGAACGCC | pPH41 | This study |

| | | | | | |
|---|---|---|---|---|---|
| fPH9 | PCR product amplified with primers oPH314/oPH138 on fDL17 template | assembly overhang (partial u6 promoter) - *cda5*target sequence - scaffold and terminator - assembly overhang (partial hsp70 promoter) | TTCAAAATTCCATTCTACAACGCTCTAGATCAGCTTCTCGGTTTTAGAGCTAGAAATAGCAAGTTAAAATAAGGCTAGTCCGTTATCAACTTGAAAAAGTGGCACCGAGTCGGTGCTTTTTTTGGTACCTGCCAAGAGTCGAACGCC | pPH42 | This study |
| fPH10 | PCR product amplified with primers oPH315/oPH316 on fDL15 template | assembly overhang (partial u6 promoter) - *cda1*target sequence - scaffold and terminator -Leu TAA tRNA promoter (UMAG_16041) - *assembly overhang (cda2 target sequence)* | TTCAAAATTCCATTCTACAACGTGTTTGATCTGCGGTAGGGAGTTTTAGAGCTAGAAATAGCAAGTTAAAATAAGGCTAGTCCGTTATCAACTTGAAAAAGTGGCACCGAGTCGGTGCTTTTTTTCGATGTGTGCACAAATCATCGTGCTAGACGCCAACGAAGCGGAAAGTTTGAGACCCGATTATAATCGGTATTGACAATTCTGTCTTCCAAACTTCATGACCAGTAGGCAAGTGGTTAAGCCGCGAGCTTTAAGCCCGATGTAGGCCCGATCTCAGCCCTTCGTTAACTCTCGTTTGTCGCGGGTTCGAATCCCGTCTGGTCAAGGTGCTCAGAGGCACGCTTG | pPH43 | This study |
| fPH11 | PCR product amplified with primers oPH317/oPH311 on fDL16 template | assembly overhang (partial Leu TAA tRNA promoter (UMAG_16041) - *cda2*target sequence - scaffold and terminator -assembly overhang (partial hsp70 promoter) | CGAATCCCGTCTGGTCAAGGTGCTCAGAGGCACGCTTGGTTTTAGAGCTAGAAATAGCAAGTTAAAATAAGGCTAGTCCGTTATCAACTTGAAAAAGTGGCACCGAGTCGGTGCTTTTTTGGTACCTGCCAAGAGTCGAACGCC | pPH43 | This study |

[a] sequences are colored in grey (assembly overhangs), red (Cas9 target sequence), blue (scaffold and terminator), green (promoter)

**Table S1E** Accession numbers

| Organism | Name | Accession number | |
|---|---|---|---|
| | | Gene | Protein |
| *Ustilago maydis* | Cda1 | UMAG_00638 | XP_011386452.1 |
| *Ustilago maydis* | Cda2 | UMAG_01143 | XP_011387091.1 |
| *Ustilago maydis* | Cda3 | UMAG_11922 | XP_011389242.1 |
| *Ustilago maydis* | Cda4 | UMAG_01788 | XP_011387743.1 |
| *Ustilago maydis* | Cda5 | UMAG_02019 | XP_011387928.1 |

| | | | |
|---|---|---|---|
| *Ustilago maydis* | Cda6 | UMAG_05792 | XP_011391520.1 |
| *Ustilago maydis* | Cda7 | UMAG_02381 | XP_011388681.1 |
| *Ustilago maydis* | *Um*PuuE | UMAG_00126 | XP_011386084.1 |
| *Ustilago maydis* | *Um*Ppi | UMAG_03726 | XP_011390187.1 |
| *Ustilago maydis* | AM1 | UMAG_01779 | XP_011387736.1 |
| *Ustilago maydis* | PM / Rsp3 | UMAG_03274 | XP_011389687.1 |
| *Ustilago maydis* | Actin | UMAG_11232 | XP_011392631.1 |
| *Colletotrichum lindemuthianum* | *Cl*Cda | AY633657 | AAT68493.1 |
| *Saccharomyces cerevisiae* | *S.c*Cda | 851016 | NP_013410.1 |
| *Pseudomonas fluorescence* | *P.f*PuuE | EU293536.1 | ACA50280.1 |
| *Pseudozyma brasiliensis* | *Pb*Cda6 | PSEUBRA_SCAF18g04655 | XP_016293102.1 |
| *Pseudozyma hubeiensis* | *Ph*Cda6 | PHSY_002331 | XP_012188345.1 |
| *Sporisorium scitamineum* | *Ss*Cda6 | SSCI30930.1 in contig CCFA01001702.1 | CDW97409.1 |
| *Bombyx mori* | *Bm*Cda1 | LOC101740647 | XP_004929283.1 |
| *Bombyx mori* | *Bm*Cda8 | LOC101735686 | XP_004923455.1 |
| *Zea mays* | *Zm*GAPDH | GRMZM2G046804 | NP_001105413.1 |
